# Spatially Characterized Aortic Proteome Reveals Novel Regional Signatures and Glucocorticoid Receptor/Dipeptidase 1 Axis in Diabetic Vasculopathy

**DOI:** 10.1002/mco2.70714

**Published:** 2026-03-30

**Authors:** Chak Kwong Cheng, Shuhui Meng, Teng Li, Huanyu Ding, Minchun Jiang, Zizhao Tian, Chi‐Fai Ng, Yin Xia, Stefan Offermanns, Yu Huang

**Affiliations:** ^1^ Department of Pharmacology Max Planck Institute for Heart and Lung Research Bad Nauheim Germany; ^2^ Department of Biomedical Sciences and Tung Biomedical Sciences Centre City University of Hong Kong Hong Kong China; ^3^ Department of Cell Biology & Institute of Biomedicine College of Life Science and Technology Jinan University Guangzhou China; ^4^ School of Biomedical Sciences Faculty of Medicine The Chinese University of Hong Kong Hong Kong China; ^5^ Department of Endocrinology Guangdong Provincial People's Hospital (Guangdong Academy of Medical Sciences) Southern Medical University Guangzhou China; ^6^ S.H. Ho Urology Centre Department of Surgery The Chinese University of Hong Kong Hong Kong China

**Keywords:** diabetes, endothelial function, inflammation, proteomics, shear stress, vasculopathy

## Abstract

Diabetes mellitus poses a major global health burden and is intricately linked to cardiovascular complications, yet the spatial molecular landscape of diabetic vasculopathy remains poorly defined. Since thoracic and abdominal aortas differ in embryological origin and hemodynamic microenvironments, we applied laser‐capture microdissection to map their spatial proteomes in health and diabetes, using a multidimensional framework across longitudinal (region) and transverse (disease) axes. This approach uncovered region‐specific protein and pathway signatures obscured by conventional bulk analyses, highlighting spatial heterogeneity in transcriptional regulators and flow‐sensitive proteins. We identified dipeptidase 1 (DPEP1), a membrane‐bound zinc metalloprotease, as selectively upregulated in the diabetic thoracic aorta and inducible by diabetic conditions and shear stress. Mechanistically, laminar shear stress promoted glucocorticoid receptor (GR) nuclear translocation to drive a GR/DPEP1 axis, potentially explaining region‐specific DPEP1 induction and its synergy with diabetic conditions. Functionally, chronic Dpep1 inhibition by cilastatin and endothelium‐specific Dpep1 knockdown attenuated neutrophilic vascular inflammation and rescued endothelial dysfunction in diabetic mice. Furthermore, the corticosteroid dexamethasone activated the shear stress‐responsive GR/DPEP1 axis in vivo, yet exerted time‐dependent vascular effects—acutely dampening neutrophilic inflammation, but chronically worsening hyperglycemia and aggravating vascular dysfunction. These findings reveal spatially defined biomarkers and highlight DPEP1 as a therapeutic target in diabetic vasculopathy.

## Graphical abstract

This study presents the first spatially resolved proteomic map of diabetic vasculopathy, identifying distinct molecular signatures in aortic regions. The glucocorticoid receptor/dipeptidase 1 (GR/DPEP1) axis was identified as a novel driver of diabetic vascular inflammation and dysfunction. DPEP1 upregulation is synergistically induced by diabetes and laminar shear stress, and its inhibition potentially alleviates vascular pathology.

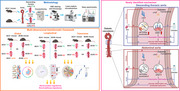

## 1 Introduction

Diabetes mellitus is a multifaceted disease, imposing a profound global health and economic burden, and affecting nearly 589 million adults (approximately one in nine adults) worldwide [1]. Alarmingly, diabetes is closely associated with vascular complications, particularly diabetic vasculopathy (e.g., diabetes‐driven atherosclerosis and diabetic retinopathy) [2]. Mechanistically, hyperglycemia contributes to diabetic vasculopathy by inducing endothelial dysfunction, oxidative stress, inflammation, and advanced glycation end‐product accumulation [2, 3]. Comprehensive proteomic profiling of diabetic versus healthy vasculature shall unveil novel biomarkers, therapeutic targets, and mechanistic insights into diabetes‐related complications.

Aorta, the largest artery in the body, exhibits significant segmental heterogeneity, with thoracic and abdominal regions differing substantially in embryological origin [4] and hemodynamic microenvironment [5]. These anatomical distinctions are amplified under diabetic conditions, where metabolic stressors and shear stress dynamics might synergistically drive region‐specific vascular remodeling [6]. Despite extensive atherosclerosis research, the link between shear stress and diabetic vasculopathy remains unclear. Hemodynamic forces fundamentally shape arterial biology that laminar shear stress (LSS) in straight segments maintains endothelial quiescence, whereas oscillatory shear stress (OSS) at bifurcations promotes endothelial dysfunction and atherogenesis [7].

Thoracic (TA) and abdominal aortas (AA) experience distinct hemodynamic environments. The descending TA is dominated by laminar flow that supports endothelial homeostasis [8]. By contrast, the AA has more branch points and frequently exhibits disturbed flow [9]. Hemodynamic forces also display segmental heterogeneity. Descending TA generally shows higher mean wall shear stress (WSS) than the AA, reflecting sustained high‐velocity flow from undivided cardiac output, whereas luminal dilation and geometric complexity reduce WSS in the AA [10, 11]. Collectively, low WSS and disturbed flow promote endothelial dysfunction, increasing abdominal aortic susceptibility to aneurysm formation [5, 9].

Despite advances in omics technologies, critical knowledge gaps persist. Conventional bulk transcriptomic and proteomic profiling of whole aortas lacks spatial resolution, potentially masking segment‐specific signatures in thoracic versus abdominal regions. A prior study has profiled tibial arteries from diabetic patients using single‐cell and spatial transcriptomics [12]. However, this study did not examine longitudinal differences along the aortic axis (e.g., thoracic vs. abdominal) over diabetes progression. In addition, previous proteomic comparisons of TA versus AA largely focused on aneurysm rather than diabetes [13], leaving diabetes‐specific vascular changes understudied. Likewise, transverse proteomic comparisons of TA and AA across healthy and diabetic states remain limited. Furthermore, how diabetes reshapes shear‐responsive proteins across aortic segments remains poorly understood.

Therefore, this study aims to (1) construct a spatially resolved proteomic atlas of diabetic vasculopathy by defining global proteomic differences between health and diabetes, and systematically mapping regional heterogeneity across the TA and AA through both longitudinal (thoracic vs. abdominal within each condition) and transverse (diabetic vs. control within each region) comparisons; (2) translate and validate key proteomic signatures by correlating the murine spatial atlas with human vascular findings; and (3) functionally interrogate prioritized, disease‐relevant proteins, particularly transcriptional regulators (TRs) and shear‐sensitive effectors, to resolve their spatial heterogeneity and clarify their mechanistic contributions to diabetic vascular inflammation and endothelial dysfunction, thereby enabling the identification of actionable biomarkers and therapeutic targets.

## 2 Results

### 2.1 Phenotypic Heterogeneity Across Vascular Segments

The aortas, and its constituent cells, such as endothelial cells (ECs) and vascular smooth muscle cells (VSMCs), exhibit significant spatial heterogeneity across different regions, particularly between the thoracic and abdominal regions [14]. However, most prior work has focused on transcriptional heterogeneity (bulk and single‐cell sequencing) [14, 15], with limited exploration of the proteomic landscape. Moreover, prior research has focused on aneurysm pathology, leaving spatial heterogeneity in non‐aneurysm conditions (e.g., diabetic vasculopathy) understudied.

**FIGURE 1:**
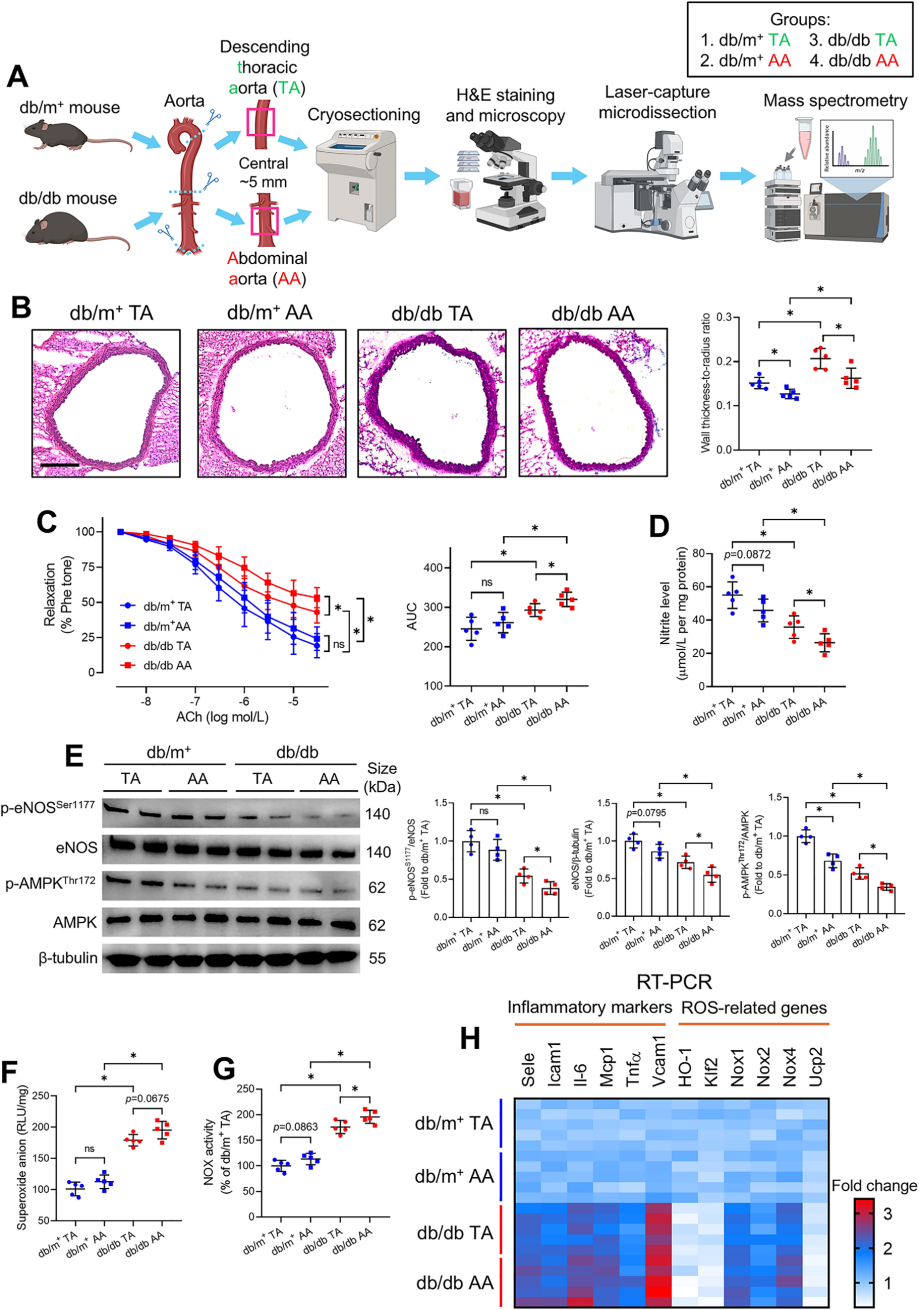
Phenotypic heterogeneity among thoracic and abdominal aortas in nondiabetic and diabetic mice. (A) Schematic overview of the study design. (B) H&E staining (Scale bar: 200 µm) and corresponding WTTR analysis of TA and AA from db/m^+^ and db/db mice. (C) Functional assay on EDRs of different mouse aortic segments by wire myograph and corresponding AUC analysis. (D) Nitrite levels in mouse thoracic and abdominal aortas (*n* = 5 per group). (E) Western blotting on mouse thoracic and abdominal aortas and corresponding quantification of expression (*n* = 4 per group). (F) Lucigenin‐enhanced chemiluminescence on ROS levels in mouse thoracic and abdominal aortas. (G) NOX activity assay in mouse thoracic and abdominal aortas. (H) Heatmap on the RT‐PCR results of inflammatory markers and ROS‐related genes in mouse thoracic and abdominal aortas (*n* = 5 per group). Data are presented as mean ± SD. ^*^ *p* < 0.05 (Brown–Forsythe and Welch ANOVA, and unpaired *t* with Welch's correction). AA, abdominal aorta; ACh, acetylcholine; AMPK, AMP‐activated protein kinase; AUC, area under the curve; EDR, endothelium‐dependent relaxation; eNOS, endothelial nitric oxide synthase; H&E, hematoxylin and eosin; NOX, NADPH oxidase; Phe, phenylephrine; RLU, relative light unit; ROS, reactive oxygen species; RT‐PCR, real‐time PCR; TA, descending thoracic aorta; WTTR, wall thickness‐to‐radius ratio.

To address these gaps, we dissected the descending TA and AA from nondiabetic (db/m^+^) and spontaneously diabetic (db/db) mice (Figure S1A) for histological assessment, phenotypic analysis, and site‐specific mass spectrometry (MS)‐based proteomics to identify site‐specific protein markers and novel therapeutic targets in diabetic vasculature (Figure 1A). Aortic arch, ascending aorta, renal arteries, and iliac arteries were excluded due to complex geometry and disturbed hemodynamics that could confound group comparisons.

To evaluate structural differences between TA and AA, we performed hematoxylin and eosin (H&E) staining on their central ∼5 mm regions to minimize edge effects from adjacent vasculature and avoid interference from branch ostia or peripheral microvessels. Diabetic db/db mice exhibited significantly higher wall thickness‐to‐radius ratio (WTTR) across both TA and AA than db/m^+^ controls (Figure 1B), indicating more severe vascular remodeling. Notably, TA showed higher WTTR than AA in both groups (*p* < 0.05), potentially aligning with TA's adaptation to withstand hemodynamic pressures and buffer pulsatile load during cardiac ejection [16].

To assess phenotypic differences, we dissected 2‐mm vascular rings from the central 5 mm of TA and AA for wire myography. Db/db mice demonstrated marked endothelial dysfunction compared to db/m^+^ mice across both segments. Furthermore, vascular function was better in TA than in AA for both db/m^+^ (non‐significant) and db/db mice (*p* < 0.05) (Figure 1C). Besides, sodium nitroprusside (SNP)‐induced endothelium‐independent relaxations were similar among segments (Figure S1B), indicating endothelial NO as the primary driver of functional differences [17].

Due to inadequate tissue from the ∼5 mm segments, whole TA and AA were harvested for subsequent molecular analyses. NO is a critical vasodilator mediating endothelial function. Consistent with myography, nitrite assays showed higher aortic NO production in db/m^+^ than db/db mice, with TA producing more NO than AA in both groups (db/m^+^: nonsignificant; db/db mice: *p* < 0.05) (Figure 1D). Western blotting of the AMP‐activated protein kinase (AMPK)/endothelial nitric oxide synthase (eNOS) axis confirmed the differences in NO production. Expression of the phosphorylated eNOS at Ser^1177^ (p‐eNOS^S1177^), total eNOS, and phosphorylated AMPK at Thr172 (p‐AMPK^Thr172^) followed the same hierarchy: db/m^+^ TA > db/m^+^ AA > db/db TA > db/db AA (Figure 1E), while total AMPK levels were comparable among groups (Figure S2).

Lucigenin chemiluminescence showed higher aortic oxidative stress in db/db than db/m^+^ mice, inversely correlating with NO production (db/db AA > db/db TA > db/m^+^ AA > db/m^+^ TA) (Figure 1F). The results were coherent to the notion that excessive reactive oxygen species (ROS) quenches NO, reducing its bioavailability and impairing endothelial function [18]. NADPH oxidase (NOX) activity showed similar pattern, higher in AA than TA in both groups (Figure 1G), supporting NOX‐driven ROS generation in diabetic vasculopathy.

Quantitative real‐time PCR (RT‐PCR) showed that: (1) db/db mice had higher inflammatory markers and NOX genes (Nox1/2/4) than db/m^+^ mice across segments; (2) within db/db mice, AA exhibited higher expression of inflammatory markers than TA; and (3) protective genes involved in ROS mitigation and shear stress sensing (*HO‐1, Klf2*, and *Ucp2*) were downregulated in db/db mice, with further reductions in AA versus TA (Figure 1H). Individual bar plots for the RT‐PCR results have been provided in Figure S3. The RT‐PCR results were consistent with the trend observed in superoxide production among aortic segments (Figure 1F,G). Notably, these transcriptional changes originate from two distinct cellular sources: resident vascular cells and infiltrating immune cells within the aortic wall. Collectively, these data imply superior TA function over AA in both health and diabetes.

### 2.2 Proteomic Patterns Across Vascular Segments

**FIGURE 2:**
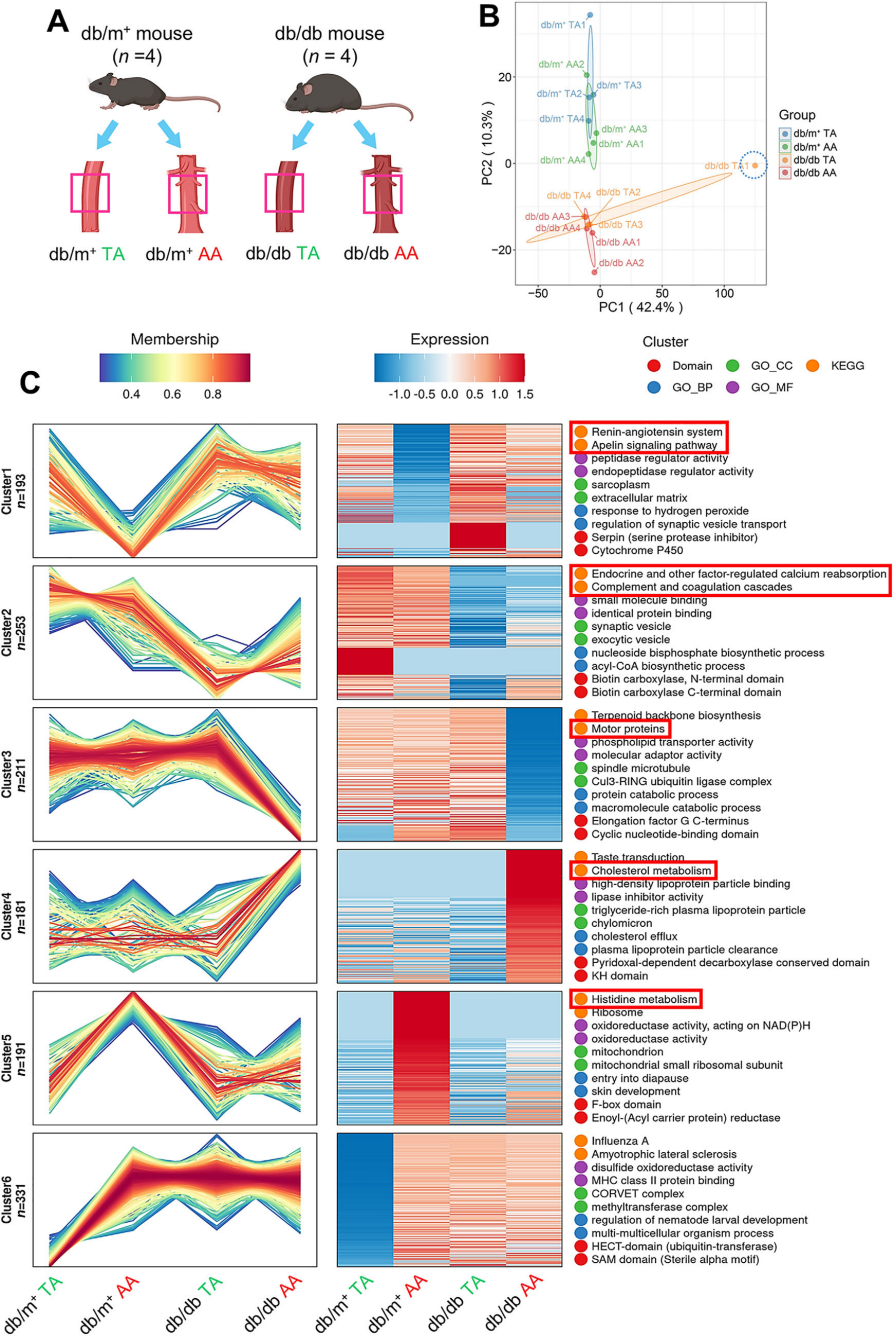
Proteomic patterns across thoracic and abdominal aortas of nondiabetic and diabetic mice. (A) Schematic representation of the aortic groups. (B) PCA analysis showing distinct clustering among the four aortic groups (*n* = 4 per group). (C) Soft clustering of expression patterns on 596 dynamically regulated proteins (SD > 0.3), characterized through KEGG, GO, and protein domains, across the four aortic groups (*n* = 4 for db/m^+^ TA, db/m^+^ AA, and db/db AA; *n* = 3 for db/db TA). AA, abdominal aorta; BP, biological process; CC, cellular component; GO, gene ontology; KEGG, Kyoto Encyclopedia of Genes and Genomes; MF, molecular function; PCA, principal component analysis; TA, descending thoracic aorta.

To assess segmental proteomic heterogeneity, we dissected the central ∼5 mm TA and AA from db/m^+^ and db/db mice (*n* = 4 individual mice per group) for laser‐capture microdissection (LCM)‐based proteomics (Figure 2A), avoiding the influence from proximal branches (e.g., brachiocephalic trunk) and distal bifurcations (e.g., iliac arteries). These regions feature strong hemodynamic disturbances (e.g., OSS) and structural heterogeneity (e.g., variable VSMC density/plasticity) [19], confounding interpretation of proteomic data. By sampling the more homogeneous central region, we minimized pre‐analytical heterogeneity, reduced batch effects, and preserved pathological relevance.

Compared with whole‐aorta proteomics, LCM precisely isolates target regions and minimizes contamination from nontarget tissues (e.g., perivascular adipose tissues). Thus, LCM captures core pathological changes that bulk analysis may mask. In this study, LCM‐based MS detected 25,778 peptides and identified 4101 proteins, where proteome coverage was comparable among groups (Figure S4), demonstrating sufficient proteome depth.

We first performed principal component analysis (PCA) to visualize segmental differences and ensure low intra‐group variability before downstream analysis. PCA revealed distinct clustering among the four groups, with TA and AA clustering more closely within db/m^+^ and db/db mice, respectively (Figure 2B). One diabetic TA sample (i.e., db/db TA1) was identified as an outlier and was excluded from downstream analysis (Figure 2B).

Given the TA‐AA anatomical gradient, we applied Mfuzz soft clustering on 596 dynamically regulated proteins (SD > 0.3) across segments in db/m^+^ and db/db mice. Mfuzz analysis identified six expression clusters, which were further annotated by enrichment analyses using three complementary frameworks, including Kyoto Encyclopedia of Genes and Genomes (KEGG), Gene Ontology (GO), and protein domains. Three distinct proteomic patterns were identified: (1) TA‐dominant, (2) AA‐dominant, or (3) transitional gradients between TA and AA, delineating a preliminary spatial proteomic landscape and segment‐specific pathway dysregulation (Figure 2C).

Notably, cluster‐specific pathway changes mirrored diabetic endothelial dysfunction, suggesting region‐specific vasculopathy risk. KEGG analysis showed that Cluster 1 was enriched in diabetic vasculature for the renin‐angiotensin system and apelin signaling (Figure 2C), pathways linked to endothelial dysfunction and vascular remodeling [20, 21]. Pathway activation strength of these two pathways followed the hierarchy: db/db TA > db/db AA > db/m^+^ TA > db/m^+^ AA (Figure 2C), consistent with WTTR results on structural remodeling (Figure 1B). In Cluster 2, two KEGG pathways were downregulated, namely endocrine and other factor‐regulated calcium reabsorption, and complement and coagulation cascades in diabetic vasculature (both TA and AA) (Figure 2C). Notably, impaired calcium signaling directly compromises vascular tone [22], and complement and coagulation cascades critically mediate vascular tone and inflammation [23]. These findings suggest homeostatic dysregulation in diabetic vasculature.

In Clusters 3 and 4, db/db AA showed downregulation of motor proteins, and upregulation of cholesterol metabolism pathways (Figure 2C), where perturbed arterial cholesterol metabolism can precede endothelial dysfunction and increase atherosclerosis and aneurysm risks [24]. In Cluster 5, histidine metabolism was markedly reduced in db/db AA versus db/m^+^ AA, with low involvement in TA (Figure 2C). Histamine upregulates eNOS expression in ECs [25], while its precursor, histidine, specifically reduces eNOS activity by hindering the substrate supply of eNOS [26]. These findings underscore AA‐specific vulnerability and metabolic reprogramming that disrupts vascular homeostasis in diabetes. Notably, since LCM‐based proteomics captures the aortic wall rather than purified cell populations, the AA‐specific proteomic changes may reflect both intrinsic molecular reprogramming in resident vascular cells and regional shifts in cellular composition (including immune infiltration).

The spatial concordance, especially AA susceptibility to diabetes‐related perturbations, suggests that regional microenvironments disproportionately shape diabetic vasculopathy.

### 2.3 Proteomic Heterogeneity Between Healthy and Diabetic Vasculature

**FIGURE 3:**
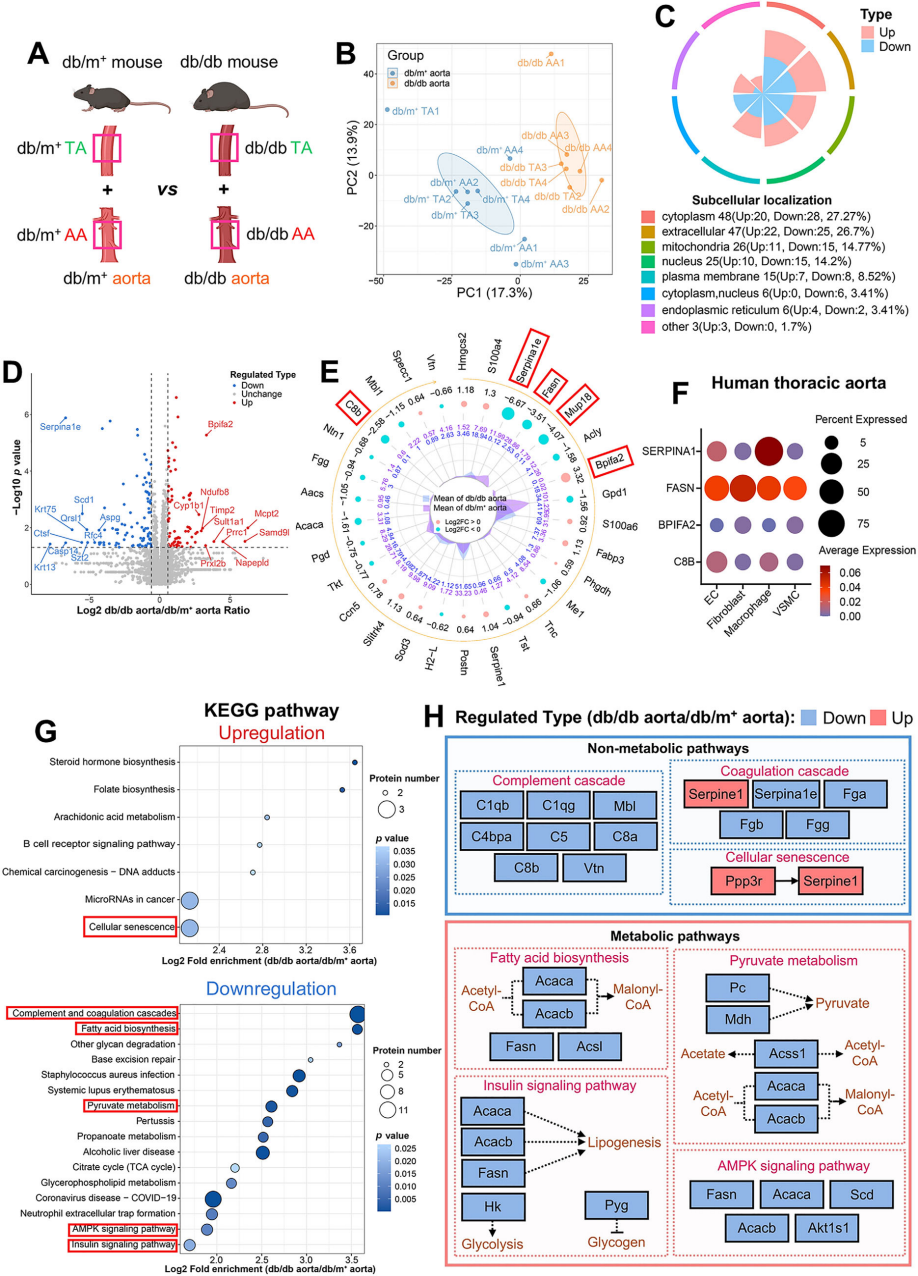
Proteomic heterogeneity between aortas of nondiabetic and diabetic mice. (A) Schematic representation of the combined aortic groups. (B) PCA analysis showing distinct clustering between the two combined groups (*n* = 8 for db/m^+^ aorta; *n* = 7 for db/db aorta). (C) Nightingale rose diagram on subcellular localizations of 77 upregulated and 100 downregulated DEPs in db/db aorta versus db/m^+^ aorta. (D) Volcano plot on DEPs between groups (Gray‐dotted lines: vertical at |Log_2_ FC| = 0.6 [∼1.5‐fold change] and horizontal at −log_10_ *p* = 1.3 [*p* < 0.05]). (E) Dynamic radar plot on top 30 DEPs between groups, ranked by *p* values (Blue dots: downregulated in db/db [Log_2_ FC < 0]; pink dots: upregulated [Log_2_ FC > 0]; central purple polygon: db/m^+^ aorta mean expression; blue: db/db aorta mean expression). (F) Dot plot on the abundance and expression of equivalent candidate markers in vascular cells of human thoracic aorta, as detected by snRNA‐seq (Dot size: percentage of cells expressing the gene; dot color: average gene expression within a cluster). (G) KEGG enrichment analysis on upregulated and downregulated pathways in db/db aorta versus db/m^+^ aorta, ranked by Log_2_ fold enrichment. (H) Overview of selected significantly enriched non‐metabolic and metabolic pathways, and the regulation of their corresponding DEPs in db/db aorta versus db/m^+^ aorta. AA, abdominal aorta; DEP, differentially expressed protein; EC, endothelial cell; KEGG, Kyoto Encyclopedia of Genes and Genomes; PCA, principal component analysis; snRNA‐seq, single‐nucleus RNA sequencing; TA, descending thoracic aorta; VSMC, vascular smooth muscle cell.

Prior to investigating segment‐specific proteomic alterations, we first assessed global differences between healthy and diabetic vasculature. Accordingly, TA and AA proteomes were pooled within db/m^+^ and db/db mice, respectively, to generate composite db/m^+^ and db/db aorta groups (Figure 3A). Analyzing TA and AA separately, instead of whole‐aorta homogenates, preserved segment‐resolved proteomes while enabling pan‐aortic comparisons, improving efficiency via single‐batch processing and reducing batch effects for better comparability.

PCA and heatmap visualization revealed clear inter‐group separation between db/m^+^ and db/db aortic proteomes (Figure 3B, Figure S5), justifying the feasibility of the integrated approach. Compared to nondiabetic vasculature, diabetic vasculature showed 77 upregulated and 100 downregulated proteins (Figure S6, Table S1), where these proteins were primarily localized to cytoplasmic, extracellular, mitochondrial, and nuclear compartments (Figure 3C). Top differentially expressed proteins (DEPs), prioritized by *p* values and fold change, were visualized by volcano and dynamic radar plots to highlight candidate markers for diabetic vasculopathy (Figure 3D,E). Five novel candidates (Serpina1e, Fasn, Mup18, Bpifa2, and C8b), that are underexplored in diabetic vasculopathy, were selected from the top 30 DEPs (Figure 3E). RT‐PCR of whole aortas confirmed transcript‐protein concordance for these markers (Figure S7), linking them to diabetic vasculopathy.

To explore the clinical relevance of these new markers, we re‐examined a previously published single‐nucleus RNA sequencing (snRNA‐seq) dataset of human TA [27]. We revealed distinct distribution patterns for these markers across vascular cell populations. SERPINA1 transcripts were predominantly detected in macrophages. FASN exhibited broad expression across multiple cell types. Mup18 was undetectable in all human vascular cell populations (Figure 3F, Figure S8).

KEGG analysis revealed heightened cellular senescence and dysregulated complement and coagulation cascades in diabetic vasculature, alongside suppressed metabolic pathways (fatty acid biosynthesis, pyruvate metabolism, AMPK signaling pathway, and insulin signaling pathway) (Figure 3G), implying metabolic reprogramming. GO pathway analysis also confirmed substantial pathway alterations in diabetic vasculature (Figure S9). Visualization of selected non‐metabolic and metabolic pathways with associated DEPs suggested profound metabolic reprogramming in diabetic vasculature, characterized by dysregulated energy metabolism and diminished lipogenesis (Figure 3H). Other key proteins linked to significantly altered KEGG pathways were outlined in a chord diagram (Figure S10).

### 2.4 Proteomic Heterogeneity Between Diabetic TA and AA

**FIGURE 4:**
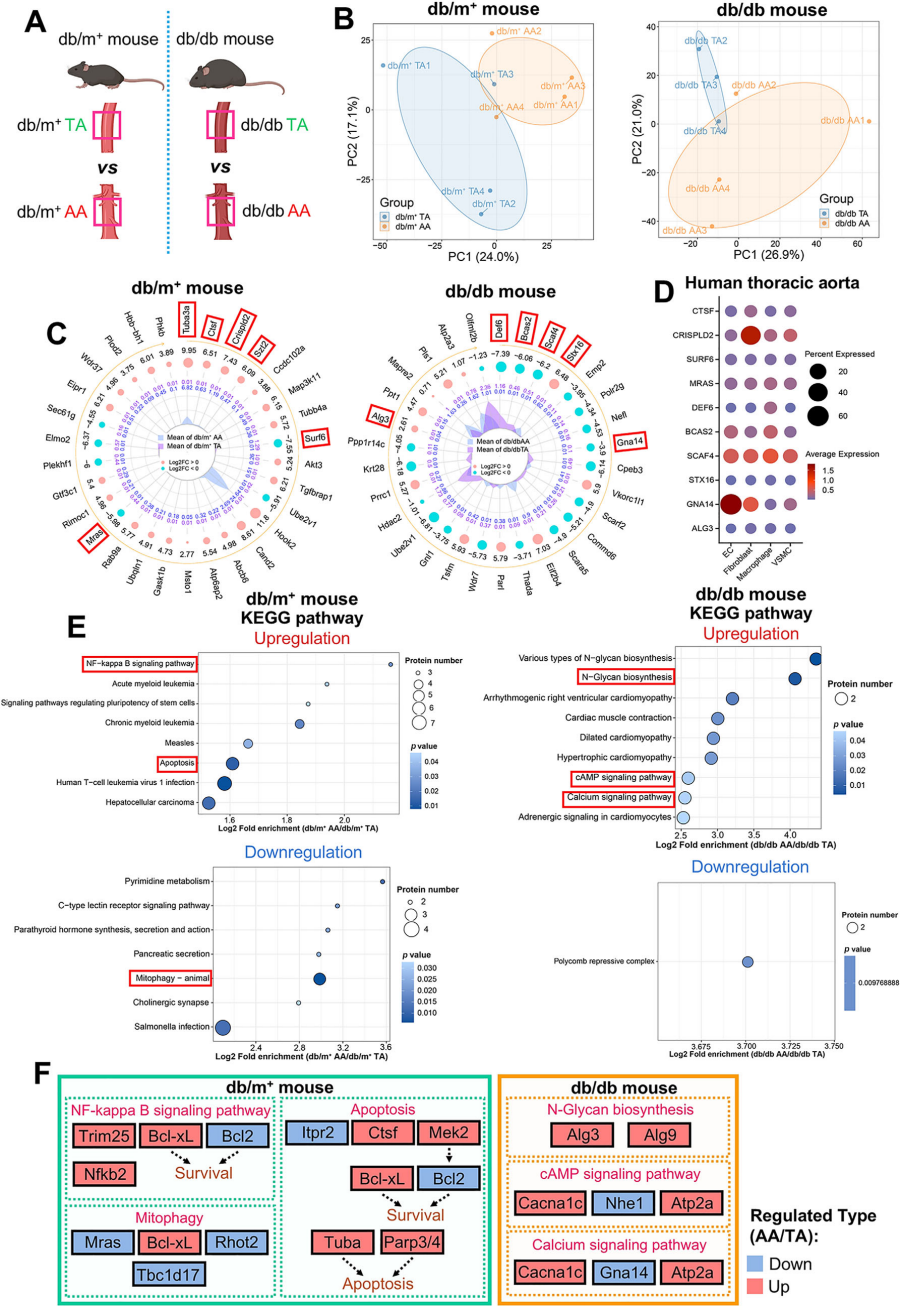
Proteomic heterogeneity between TA and AA of nondiabetic and diabetic mice. (A) Schematic representation of the comparison groups. (B) PCA analysis showing distinct clustering between TA and AA of db/m^+^ and db/db mice (*n* = 4 for db/m^+^ TA, db/m^+^ AA, and db/db AA; *n* = 3 for db/db TA). (C) Dynamic radar plots on top 30 DEPs between groups, ranked by *p* values (Blue dots: downregulated in AA [Log_2_ FC < 0]; pink dots: upregulated [Log_2_ FC > 0]; central purple polygon: TA mean expression; blue: AA mean expression). (D) Dot plot on the abundance and expression of equivalent candidate markers in vascular cells of human thoracic aorta, as detected by snRNA‐seq (Dot size: percentage of cells expressing the gene; dot color: average gene expression within a cluster). (E) KEGG enrichment analysis on upregulated and downregulated pathways in AA versus TA, ranked by Log_2_ fold enrichment. (F) Overview of selected significantly enriched pathways, and the regulation of their corresponding DEPs in AA versus TA. AA, abdominal aorta; DEP, differentially expressed protein; EC, endothelial cell; KEGG, Kyoto Encyclopedia of Genes and Genomes; PCA, principal component analysis; snRNA‐seq, single‐nucleus RNA sequencing; TA, descending thoracic aorta; VSMC, vascular smooth muscle cell.

We next compared TA and AA proteomes within db/m^+^ and db/db mice to investigate segment‐specific differences (Figure 4A). PCA and heatmaps revealed clear segregation of TA and AA proteomes in both cohorts (Figure 4B, Figure S11), indicating segmental proteomic heterogeneity. In db/m^+^ mice, AA exhibited 180 significantly upregulated and 41 downregulated proteins versus TA. In db/db mice, AA showed 26 upregulated and 46 downregulated proteins (Figure S12, Tables S2 and S3). Segmental DEPs were mainly localized to the cytoplasm, nucleus, plasma membrane, and mitochondria (Figure S13).

Volcano (Figure S14) and dynamic radar plots (Figure 4C) were employed to identify candidate regional markers. Six novel candidates were selected from the top 30 DEPs (AA vs. TA) of db/m^+^ (Tuba3a, Ctsf, Crispld2, Szt2, Surf6, and Mras) and db/db mice (Def6, Bcas2, Scaf4, Stx16, Gna14, and Alg3), respectively (Figure 4C). RT‐PCR validation demonstrated concordant trends in the transcription of these markers (Figure S15). Notably, these markers have been scarcely implicated in vascular spatial heterogeneity. We further mapped these markers across vascular cell types in a human TA snRNA‐seq dataset. While Tuba3a and Stz2 were undetectable in human vascular cells, CRISPLD2 and GNA14 were highly enriched in fibroblasts and ECs, respectively (Figure 4D, Figure S16). Any overall region‐specific expression alterations reflect the aggregated changes in these individual cell types.

KEGG analysis revealed upregulation of NF‐kappa B signaling and apoptosis pathways, alongside downregulation of mitophagy, in AA versus TA of db/m^+^ mice (Figure 4E). In db/db mice, AA exhibited upregulation of N‐glycan biosynthesis, cAMP signaling pathway, and calcium signaling pathway (Figure 4E). N‐glycans, complex oligosaccharide modifications covalently attached to proteins, are potentially functional effectors in diabetic cardiovascular diseases [28]. Moreover, dysregulated calcium signaling in TA might contribute to its greater susceptibility to diabetes‐related medial calcification than AA [29]. GO pathway analysis also revealed substantial pathway divergence between TA and AA in health and diabetes (Figures S17 and S18).

Selected critical pathways and associated DEPs were displayed to delineate vascular heterogeneity between TA and AA in both cohorts (Figure 4F). Importantly, our validated regulators Mras and Alg3 were involved in mitophagy and N‐glycan biosynthesis, respectively (Figure 4F). Other key proteins linked to significantly altered KEGG pathways were outlined in chord diagrams (Figure S19).

### 2.5 Proteomic Heterogeneity in TA and AA Between Healthy and Diabetic Vasculature

**FIGURE 5:**
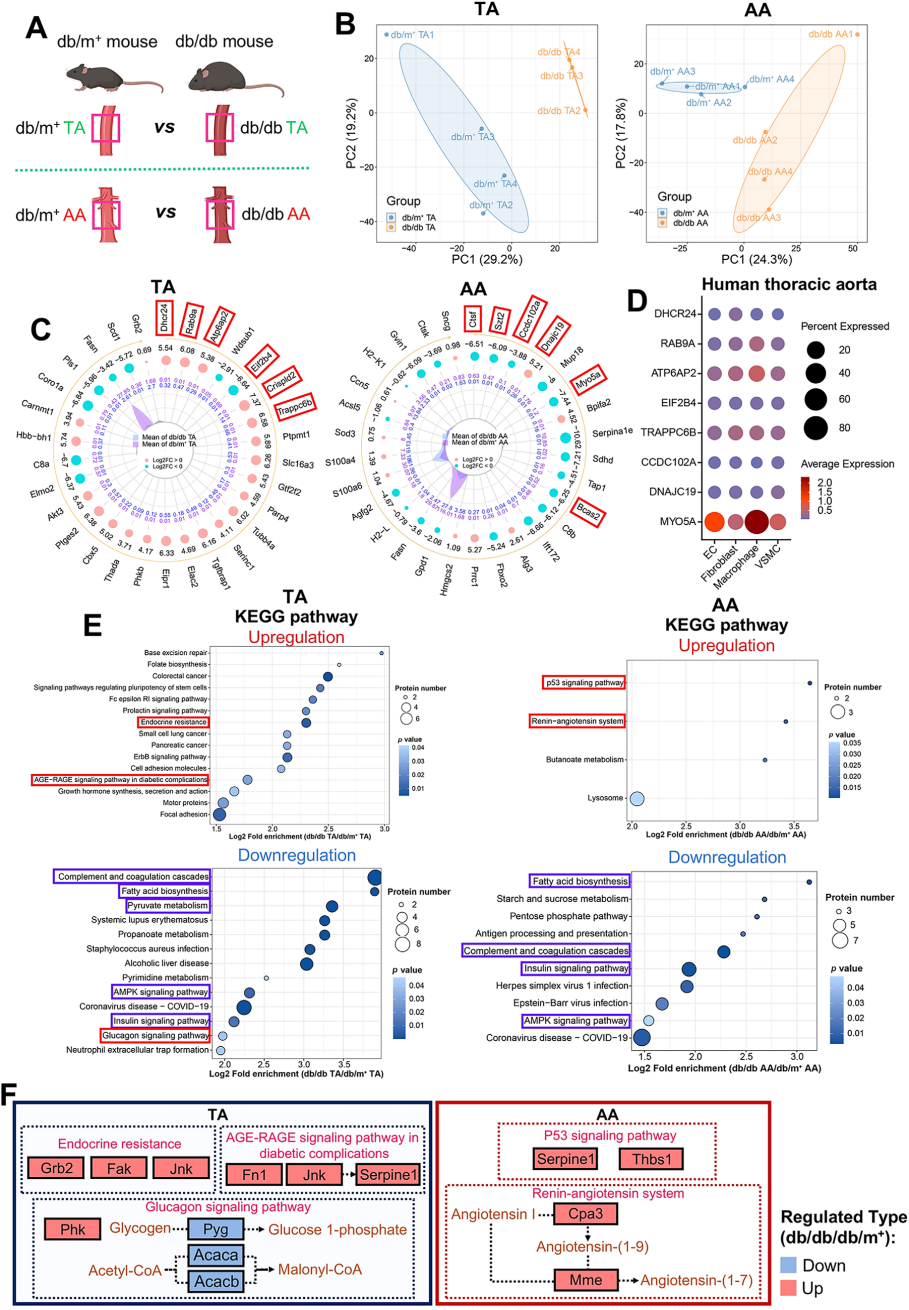
Proteomic heterogeneity in TA and AA between nondiabetic and diabetic mice. (A) Schematic representation of the comparison groups. (B) PCA analysis showing distinct clustering in TA and AA between db/m^+^ and db/db mice (*n* = 4 for db/m^+^ TA, db/m^+^ AA, and db/db AA; *n* = 3 for db/db TA). (C) Dynamic radar plots on top 30 DEPs between groups, ranked by *p* values (Blue dots: downregulated in AA [Log_2_ FC < 0]; pink dots: upregulated [Log_2_ FC > 0]; central purple polygon: db/m^+^ mean expression; blue: db/db mean expression). (D) Dot plot on the abundance and expression of equivalent candidate markers in vascular cells of human thoracic aorta, as detected by snRNA‐seq (Dot size: percentage of cells expressing the gene; dot color: average gene expression within a cluster). (E) KEGG enrichment analysis on upregulated and downregulated pathways in TA and AA of db/db mice versus db/m^+^ mice, ranked by Log_2_ fold enrichment. (F) Overview of selected significantly enriched pathways, and the regulation of their corresponding DEPs in TA and AA of db/db mice versus db/m^+^ mice. AA, abdominal aorta; DEP, differentially expressed protein; EC, endothelial cell; KEGG, Kyoto Encyclopedia of Genes and Genomes; PCA, principal component analysis; snRNA‐seq, single‐nucleus RNA sequencing; TA, descending thoracic aorta; VSMC, vascular smooth muscle cell.

We transversely investigated proteomic differences in both TA (db/m^+^ TA vs. db/db TA) and AA (db/m^+^ AA vs. db/db AA) of both cohorts (Figure 5A). PCA and heatmaps indicated proteomic heterogeneity in TA and AA between db/m^+^ and db/db mice (Figure 5B, Figure S20). Compared to db/m^+^ counterparts, db/db TA had 128 upregulated and 52 downregulated proteins, while db/db AA had 64 upregulated and 113 downregulated proteins (Figure S21, Tables S4 and S5). DEPs were primarily localized to cytoplasmic, extracellular, nuclear, and mitochondrial compartments (Figure S22).

Candidate markers identified in db/db TA (Dhcr24, Rab9a, Atp6ap2, Eif2b4, Crispld2, Trappc6b) and db/db AA (Ctsf, Szt2, Ccdc102a, Dnajc19, Myo5a, Bcas2) using volcano (Figure S23) and dynamic radar plots (Figure 5C) were validated by RT‐PCR, confirming concordant transcriptional and proteomic trends (Figure S24). These markers potentially represent novel candidates associated with vascular spatial heterogeneity. Human TA snRNA‐seq data revealed expression of these markers in major vascular cells, with MYO5A enriched in macrophages (Figure 5D, Figure S25).

Beyond the key metabolic pathway downregulations already noted in the global comparison between nondiabetic and diabetic vasculature (e.g., fatty acid biosynthesis and AMPK signaling pathway) (Figure 3G), diabetic TA and AA exhibited additional region‐specific pathway alterations. Specifically, diabetic TA showed increased endocrine resistance and AGE‐RAGE signaling, with reduced glucagon signaling. Conversely, diabetic AA displayed upregulation of the p53 signaling pathway and the RAS (Figure 5E,F). GO analysis also revealed distinct region‐specific pathway enrichment in diabetic TA and AA (Figures S26 and S27). Key proteins associated with other significantly regulated KEGG pathways were depicted in chord diagrams (Figure S28).

### 2.6 TR Heterogeneity Across Vascular Regions in Health and Diabetes

**FIGURE 6:**
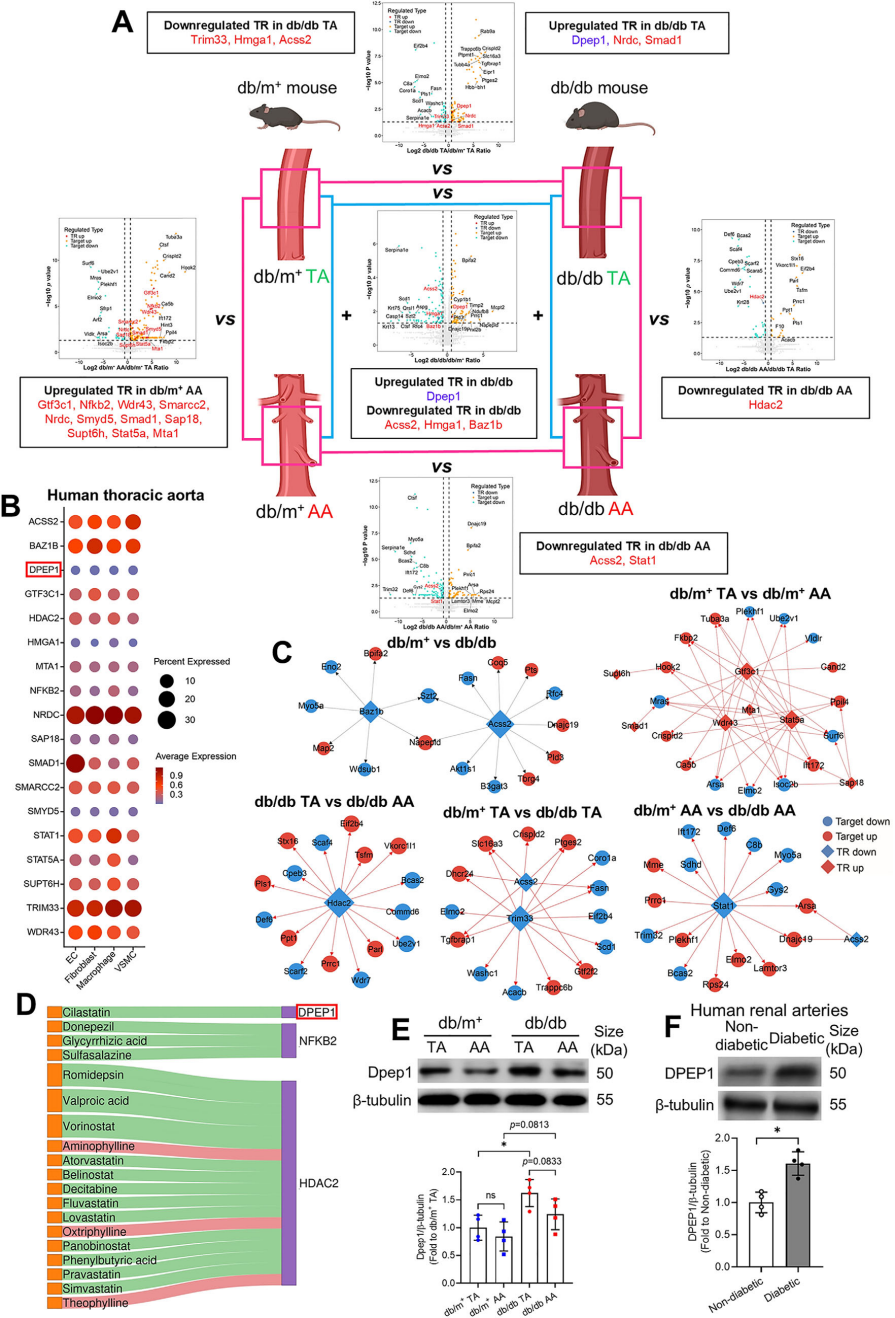
TR heterogeneity among thoracic and abdominal aortas in nondiabetic and diabetic mice. (A) Volcano plots on differentially expressed TRs and their targets among the four aortic groups (Gray‐dotted lines: vertical at |Log2 FC| = 0.6 [∼1.5‐fold change] and horizontal at −log10 *p* = 1.3 [*p* < 0.05]). (B) Dot plot on the abundance and expression of equivalent TRs in vascular cells of human thoracic aorta, as detected by snRNA‐seq (Dot size: percentage of cells expressing the gene; dot color: average gene expression within a cluster). (C) PPI networks on certain differentially expressed TRs and their known downstream targets being significantly altered among different group comparisons (Diamond: TR; circle: target). (D) Sankey plot on predicted interactions between FDA‐approved drugs and certain TRs with defined mechanisms (Orange node: FDA‐approved drug; purple node: TR; red link: activation; green link: inhibition). (E) Western blotting on Dpep1 expression in mouse TA and AA, and corresponding quantification. (F) Western blotting on DPEP1 expression in renal arteries of nondiabetic and diabetic patients, and corresponding quantification (*n* = 4 per group). Data are presented as mean ± SD. ^*^ *p* < 0.05 (Two‐tailed unpaired *t*‐tests and nonparametric Mann–Whitney tests for two‐group comparison; Brown–Forsythe and Welch ANOVA, and unpaired *t* with Welch's correction for multiple‐group comparison). AA, abdominal aorta; DPEP1, dipeptidase 1; EC, endothelial cell; PPI, protein–protein interaction; snRNA‐seq, single‐nucleus RNA sequencing; TA, descending thoracic aorta; TR, transcriptional regulator; VSMC, vascular smooth muscle cell.

Since TRs govern pathway outputs via target‐gene regulation, we profiled diabetes‐induced TR dysregulation across vascular regions. TR expression exhibited pronounced TA‐AA heterogeneity in health and diabetes (Figure S29). 18 TRs were differentially regulated in specific vascular regions, accompanied by their predicted downstream targets (Figure 6A, Figure S30). RT‐PCR validated that transcriptional changes for these TRs mirrored proteomic patterns across comparisons (Figure S31).

We revealed several TRs not previously implicated in diabetic vasculature (e.g., Dpep1, Smarcc2, Smyd5, Supt6h, Trim33, Wdr43). Beyond validating classical TRs (e.g., Hdac2, Smad1, Mta1, Stat1) involved in mediating vascular dysfunction, we unveiled compartmentalized expression signatures, adding spatial context to their pathophysiological roles (Figure 6A). All 18 TRs were detected within major vascular cell types of human TA (Figure 6B, Figures S32 and S33). Notably, snRNA‐seq may underestimate total expression since it captures only nuclear transcripts and excludes cytosolic RNA. Therefore, the snRNA‐seq results reflect nuclear transcriptional traces in human vascular cells rather than total cellular expression (Figure 6B).

Protein–protein interaction (PPI) networks integrated perturbed TRs with functionally annotated target genes exhibiting significant expression changes (Figure 6C). Notably, certain well‐established TRs were predicted upstream of novel diabetic vascular markers identified in prior sections (e.g., Bpifa2, Dnajc19, Myo5a, Szt2) (Figure 6C). Other newly identified TRs, though functionally uncharacterized, might serve as candidate biomarkers and therapeutic targets for diabetic vasculopathy.

Among the 18 TRs, dipeptidase 1 (Dpep1) is a membrane‐bound dipeptidase highly expressed in renal proximal tubular cells and peritubular capillaries, mediating substrate hydrolysis. DPEP1 is known to mediate renal, pulmonary, and hepatic inflammation [30, 31] and has been newly implicated in diabetic vasculopathy. Drug‐target analysis suggested that some FDA‐approved drugs might target TRs such as HDAC2 (Figure 6D), a known regulator of endothelial dysfunction [32]. Cilastatin is a clinical DPEP1 inhibitor for acute kidney injury (Figure 6D); however, whether it confers similar vascular benefits remains unexplored. Notably, Dpep1 was elevated in both systemic diabetic vasculature and diabetic TA (Figure 6A), suggesting that focal dysregulation may drive systemic changes that whole‐aorta analyses may obscure. Western blotting confirmed TA‐specific Dpep1 upregulation in db/db mice (Figure 6E), supporting region‐dependent TR elevation in diabetes. Coherently, DPEP1 was upregulated in renal arteries from diabetic patients (Figure 6F). Unlike human snRNA‐seq data, our Western blot findings showed relatively higher DPEP1 expression in human vasculature, hinting at the technical limitation of snRNA‐seq in capturing total expression.

### 2.7 Dpep1‐Mediated Vascular Inflammation and Endothelial Dysfunction in Diabetes

Beyond hyperglycemia, diabetes is associated with elevated circulating lipopolysaccharide (LPS). Given that LPS can induce renal DPEP1 [30], and DPEP1 was upregulated in diabetic vasculature (Figure 6A), we tested whether hyperglycemia and LPS induce vascular Dpep1 expression, including segment‐specific effects on TA and AA.

**FIGURE 7:**
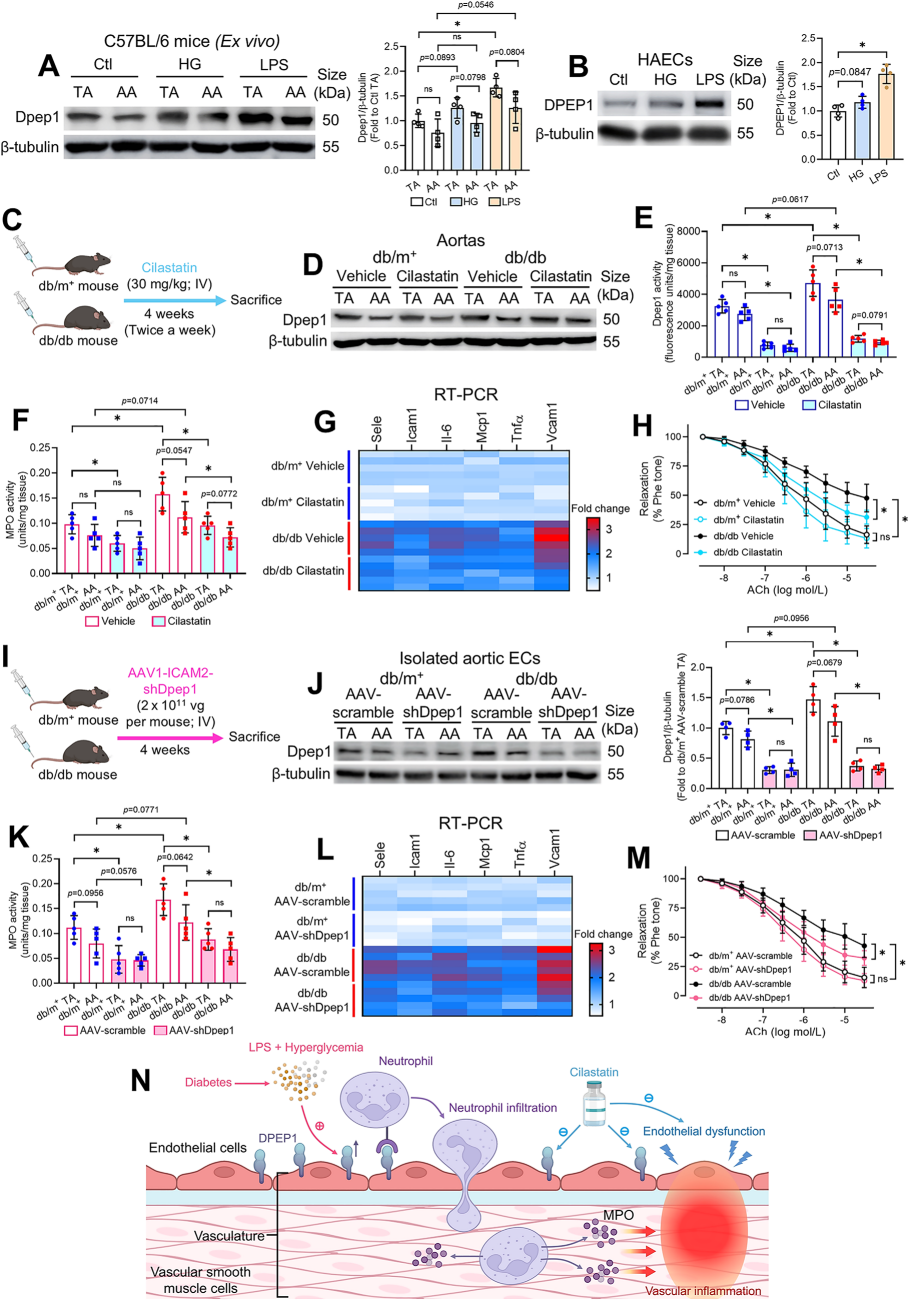
Vascular inflammation and endothelial dysfunction driven by diabetes‐related Dpep1 upregulation. (A) Western blotting on Dpep1 expression in TA and AA from C57BL/6 mice following ex vivo treatment with HG and LPS, and corresponding quantification. (B) Western blotting on DPEP1 expression in HG‐ and LPS‐treated HAECs, and corresponding quantification. (C) Schematic overview of chronic cilastatin treatment in diabetic mice. (D) Western blotting on Dpep1 expression in TA and AA from nondiabetic and diabetic mice following chronic cilastatin treatment (*n* = 4 per group). (E) Dpep1 activity in TA, and (F) MPO activity in TA and AA from db/m^+^ and db/db mice following chronic cilastatin treatment. (G) Heatmap on the RT‐PCR results of inflammatory markers in mouse aortas after cilastatin treatment. (H) Functional assay on EDRs of aortas from cilastatin‐treated mice by wire myograph (*n* = 5 per group). (I) Schematic overview of intravenous AAV1‐ICAM2‐shDpep1 injection in diabetic mice. (J) Western blotting on Dpep1 expression in isolated endothelial cells from TA and AA of nondiabetic and diabetic mice subjected to AAV‐shDpep1 injection, and corresponding quantification (*n* = 4 per group). (K) MPO activity in TA and AA from AAV‐shDpep1‐injected db/m^+^ and db/db mice. (L) Heatmap on the RT‐PCR results of inflammatory markers in mouse aortas from AAV‐shDpep1‐injected mice. (M) Functional assay on EDRs of aortas from AAV‐shDpep1‐injected mice by wire myograph (*n* = 5 per group). (N) Schematic overview of diabetes‐related endothelial Dpep1 upregulation and neutrophil‐released MPO in promoting vascular inflammation and endothelial dysfunction. Data are presented as mean ± SD. ^*^ *p* < 0.05 (Brown–Forsythe and Welch ANOVA, and unpaired *t* with Welch's correction). AA, abdominal aorta; AAV, adeno‐associated virus; ACh, acetylcholine; DPEP1, dipeptidase 1; EC, endothelial cell; HAEC, human aortic endothelial cell; HG, high glucose; IV, intravenous; LPS, lipopolysaccharide; MPO, myeloperoxidase; Phe, phenylephrine; TA, descending thoracic aorta.

Following 24‐h ex vivo incubation, high glucose (HG; 25 mmol/L) slightly, while LPS (1 µg/mL) significantly increased Dpep1 expression in TA and AA of C57BL/6 mice (Figure 7A), where mannitol (osmotic control) did not alter Dpep1 expression (Figure S34). Crucially, TA showed higher basal and induced Dpep1 expression than AA in all groups, implying that while hyperglycemia and LPS systemically induce vascular Dpep1 expression, neither explains the intrinsic TA‐AA difference. Given DPEP1 expression in different vascular cells (Figure 6B) and its pro‐inflammatory receptor role in pulmonary and hepatic ECs [31], we interrogated whether hyperglycemia or LPS upregulates DPEP1 in aortic ECs. Consistent with ex vivo findings, LPS caused stronger DPEP1 induction than HG in human aortic endothelial cells (HAECs) (Figure 7B).

Since cilastatin is a clinically used DPEP1 inhibitor that suppresses DPEP1 enzymatic activity rather than expression (Figure 6D), we investigated its chronic effects on diabetic vasculature. Four‐week intravenous injection of cilastatin (30 mg/kg) did not alter body weight, glucose homeostasis, or serum LPS levels in db/m^+^ and db/db mice (Figure 7C, Figure S35), indicating metabolic neutrality. Chronic cilastatin injection did not reduce aortic Dpep1 expression in either db/m^+^ or db/db mice (Figure 7D, Figure S36). Instead, cilastatin significantly mitigated Dpep1 enzymatic activity in both TA and AA across genotypes (Figure 7E), confirming effective target engagement.

In pulmonary and hepatic vasculature, endothelial Dpep1 functions as a surface receptor that mediates neutrophil recruitment, adhesion, and tissue infiltration, where activated neutrophils release myeloperoxidase (MPO) to amplify inflammatory responses and tissue damage [31]. Given that diabetes is associated with exacerbated neutrophil recruitment and neutrophil‐driven damage in the vasculature [33], and pharmacological inhibition of Dpep1 remarkably reduces neutrophil adhesion during renal injury [30], we therefore quantified MPO activity, an established neutrophil marker, in TA and AA from cilastatin‐treated mice. Importantly, chronic cilastatin treatment inhibited MPO activity in both diabetic TA and AA (Figure 7F), supporting the non‐segmental action of cilastatin on neutrophilic inflammation. Consistent with the reduced MPO activity, 4‐week cilastatin treatment diminished inflammatory marker expression (Figure 7G, Figure S37), and improved endothelium‐dependent vascular function in db/db aortas (Figure 7H), without altering SNP‐induced endothelium‐independent relaxations (Figure S38). Collectively, these findings hint that cilastatin potentially mitigates diabetes‐related vascular injury.

To rule out indirect vascular effects due to renal modulation, we employed endothelium‐specific Dpep1 knockdown through intravenous administration of adeno‐associated virus (AAV)‐ICAM2‐shDpep1 in diabetic mice (Figure 7I). Whole‐aorta Western blots showed Dpep1 downregulation (Figure S39), and purified CD31^+^ ECs confirmed robust knockdown efficiency in TA and AA (Figure 7J). AAV‐mediated endothelial Dpep1 knockdown did not alter body weights and glucose parameters in diabetic mice (Figure S40).

Endothelium‐specific Dpep1 knockdown significantly repressed MPO activity in diabetic TA (Figure 7K), attenuated vascular inflammation (Figure 7L, Figure S41), and alleviated endothelial dysfunction in diabetic vasculature (Figure 7M), without altering SNP‐induced vasorelaxation (Figure S42). Collectively, we demonstrated that hyperglycemia and LPS increased endothelial Dpep1 to drive MPO‐linked neutrophilic inflammation, whereas cilastatin inhibited Dpep1 and restored endothelial function without altering metabolic parameters (Figure 7N).

### 2.8 Shear Stress‐Mediated and Dexamethasone‐Induced GR/DPEP1 Axis

**FIGURE 8:**
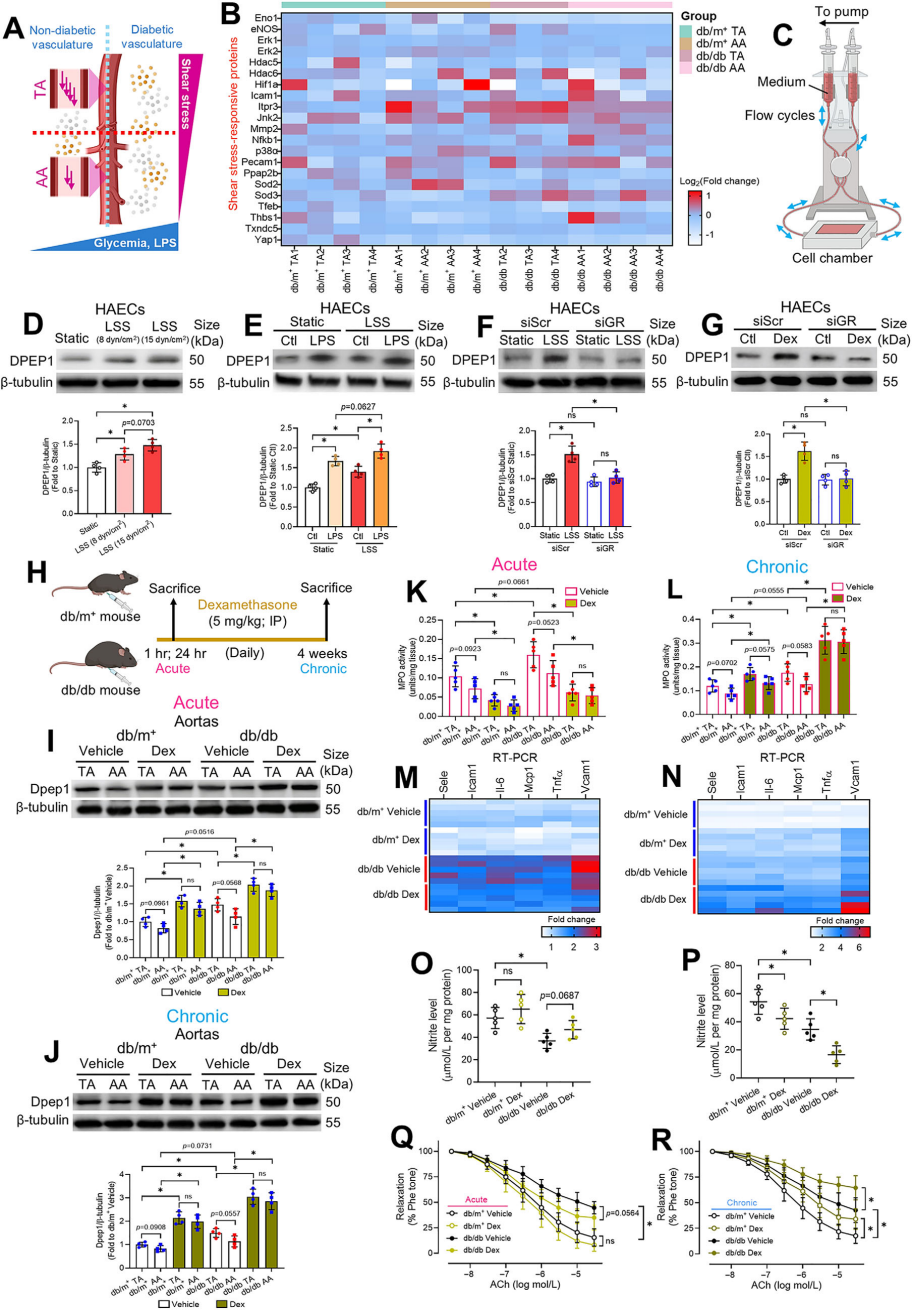
GR/DPEP1 axis induced by LSS and dexamethasone treatment. (A) Schematic representation of the alterations in shear stress magnitudes, glycemia, and circulatory LPS levels in TA and AA of the diabetic vasculature. (B) Heatmap on the protein expression of shear‐sensitive proteins in TA and AA of db/m^+^ and db/db mice (*n* = 4 for db/m^+^ TA, db/m^+^ AA, and db/db AA; *n* = 3 for db/db TA). (C) Schematic diagram on the design of ibidi flow system and flow chamber for in vitro simulation of shear stress. Western blotting on DPEP1 expression in HAECs subjected to (D) different LSS magnitudes, (E) LSS and LPS exposure, (F) siRNA‐mediated GR knockdown and subsequent LSS exposure, and (G) siRNA‐mediated GR knockdown and subsequent dexamethasone treatment. (H) Schematic overview of acute and chronic dexamethasone treatment in diabetic mice. Western blotting on aortic Dpep1 expression of db/m^+^ and db/db mice subjected to (I) acute, and (J) chronic dexamethasone treatment (*n* = 4 per group). MPO activity in TA and AA from db/m^+^ and db/db mice subjected to (K) acute, and (L) chronic dexamethasone treatment. Heatmap on the RT‐PCR results of inflammatory markers in mouse aortas upon (M) acute, and (N) chronic dexamethasone treatment. Aortic nitrite levels in mice upon (O) acute, and (P) chronic dexamethasone treatment. Functional assay on EDRs of aortas from mice upon (Q) acute, and (R) chronic dexamethasone treatment by wire myograph (*n* = 5 per group). Data are presented as mean ± SD. ^*^ *p* < 0.05 (Brown–Forsythe and Welch ANOVA, and unpaired *t* with Welch's correction). AA, abdominal aorta; ACh, acetylcholine; Dex, dexamethasone; DPEP1, dipeptidase 1; EDR, endothelium‐dependent relaxation; GR, glucocorticoid receptor; HAEC, human aortic endothelial cell; IP, intraperitoneal; LPS, lipopolysaccharide; LSS, laminar shear stress; MPO, myeloperoxidase; Phe, phenylephrine; siScr, scrambled siRNA; TA, descending thoracic aorta.

While hyperglycemia and LPS systemically elevate vascular DPEP1, its noticeable upregulation in the diabetic TA suggests region‐specific regulation. Given the inherent differences in hemodynamic shear stress between TA and AA, we hypothesize that diabetic conditions synergize with mechanical forces to drive preferential DPEP1 upregulation in TA (Figure 8A).

Segment‐specific regulation of shear‐sensitive genes under diabetic conditions remains underexplored. We therefore profiled the expression of well‐established shear‐sensitive proteins across healthy and diabetic vasculature (Figure 8B). Notably, the expression of these proteins differed between high‐shear stress TA and low‐shear stress AA in both conditions. Distinct, and sometimes opposite, expression trends were observed for specific proteins (e.g., Itpr3 and Thbs1) (Figure 8B), suggesting complex crosstalk between metabolic and biomechanical signaling in diabetic vasculature.

To study the effect of shear stress on DPEP1, we employed a custom‐built flow chamber to simulate laminar flow pattern in vitro (Figure 8C), as previously described [34]. To recapitulate TA‐AA hemodynamics, we first exposed HAECs to LSS at different intensities. A positive correlation between shear stress magnitude and DPEP1 expression was observed (Figure 8D), indicating that DPEP1 is shear‐sensitive. To simulate the pathophysiological condition of diabetic TA, HAECs were concomitantly exposed to high LSS (15 dyn/cm^2^) and LPS. This co‐exposure further increased DPEP1 expression (Figure 8E), providing a potential mechanism for elevated DPEP1 level in diabetic TA.

We next explored the mechanism linking LSS to DPEP1 upregulation. Previous work showed that the corticosteroid dexamethasone induces renal DPEP1 expression in a glucocorticoid receptor (GR)‐dependent manner [35]. Furthermore, LSS promotes GR nuclear translocation to modulate target gene expression in ECs [36]. We therefore posited a signaling axis where LSS drives GR nuclear translocation to induce DPEP1 expression. Since GR nuclear translocation is a rapid response, we assessed GR levels in nuclear/cytoplasmic fractions after 1‐h LSS exposure, which significantly promoted GR nuclear import (Figure S43). Crucially, GR knockdown in HAECs diminished GR levels and nuclear localization, and abrogated DPEP1 upregulation after 24‐h LSS exposure (Figure 8F, Figure S43). Coherently, the GR agonist dexamethasone significantly induced GR nuclear import and increased DPEP1 expression, where such effect was ablated in GR‐knockdown HAECs (Figure 8G, Figure S44). These results confirmed the necessity of GR in inducing DPEP1 expression.

We next studied the GR/DPEP1 axis in vivo. Given the acute anti‐inflammatory yet chronic diabetogenic effects of dexamethasone [37], we investigated its short‐term (24‐h) and long‐term (4‐week) effects on diabetic vasculature (Figure 8H), and the functional involvement of the GR/DPEP1 axis. Notably, acute dexamethasone treatment slightly raised blood glucose levels in db/db mice, without altering body weights (Figure S45). Conversely, 4‐week chronic dexamethasone treatment retarded weight gain and exacerbated hyperglycemia in both db/m^+^ and db/db mice (Figure S46), confirming adverse diabetogenic effects.

Both acute and chronic dexamethasone treatment significantly upregulated Dpep1 in TA and AA of both cohorts (Figure 8I,J), implying a systemic vascular effect. Alongside, both regimens increased aortic GR nuclear translocation, more pronounced in TA than AA (Figures S47 and S48), supporting shear‐enhanced GR/DPEP1 activation in vivo. Strikingly, dexamethasone time‐dependently exerted anti‐inflammatory and pro‐inflammatory effects on the vasculature. Acute dexamethasone treatment significantly reduced MPO activity and the expression of inflammation markers in aortas, whereas chronic treatment increased both (Figure 8K–N, Figures S49 and S50).

Despite robustly inducing GR nuclear translocation and DPEP1 upregulation, a protein we have established as sufficient to promote MPO release (Figure 7), acute dexamethasone treatment overall reduced vascular inflammation. This suggests dexamethasone's broad anti‐inflammatory program might override the pro‐inflammatory contributions of individual pathways, such as the GR/DPEP1 axis. In contrast, chronic dexamethasone treatment worsens diabetes, thereby aggravating vascular pathology through mechanisms like heightened hyperglycemia and circulating LPS. This implied that the aggravated vascular damage might primarily stem from diabetes‐related metabolic deterioration, rather than GR/DPEP1‐driven inflammation alone. Nevertheless, our findings demonstrated that LSS and dexamethasone are sufficient to activate the GR/DPEP1 axis. However, the causality between the GR/DPEP1 axis and neutrophilic inflammation remains unproven, since both interventions exert broad, pleiotropic effects beyond DPEP1 regulation.

In addition, acute dexamethasone treatment modestly increased NO production and eNOS phosphorylation in db/db aortas, whereas chronic treatment markedly suppressed NO production, total eNOS, and eNOS phosphorylation (Figure 8O,P, Figures S51 and S52). Consistently, acute dexamethasone treatment slightly improved, while chronic treatment significantly impaired EDRs in db/db aortas (Figure 8Q,R). The unaltered responses to SNP confirmed that the deficit lay specifically in endothelial signaling (Figures S53 and S54). These results align with established literature documenting eNOS inactivation after prolonged dexamethasone exposure [38] and highlight the vascular toxicity of chronic corticosteroid administration.

## 3 Discussion

### 3.1 Potential Value of LCM‐Based Spatial Proteomics

This study deliberately employed LCM‐based MS to resolve spatially distinct proteomic signatures in diabetic vasculature. Compared to conventional bulk proteomics, this approach ensures precise isolation of target tissues, thereby eliminating confounding signals from adjacent tissues and enhancing sample homogeneity. In this study, LCM‐based proteomics achieved comparable proteomic depth and sensitivity to bulk proteomics (4101 proteins identified). Therefore, the balanced study design, with precision optimization, contamination prevention, and assurance of proteomic depth, might more effectively reduce potential false positives than bulk proteomics. Another methodological strength of this study is using LCM‐MS to profile TA and AA separately. This strategy preserves spatial information of aortic segments and enables the integrative analysis of proteomic data. This spatial resolution is unattainable using conventional bulk proteomics on whole aorta homogenates, which inherently masks spatially restricted markers due to signal averaging of anatomically distinct regions.

### 3.2 Study Design Considerations

When assessing global differences between healthy and diabetic vasculature, our sampling strategy inherently excluded structurally complex regions, specifically the aortic arch and iliac bifurcation, which potentially confound results through nondiabetic biomechanical effects. This deliberate focus targeted segments central to diabetes‐specific pathology (i.e., TA and AA). Our goal prioritized identifying core molecular signatures over comprehensive anatomical mapping. Conversely, the relevance of the aortic arch and iliac bifurcation to diabetic vasculopathy is less established. Including these regions risks diluting the diabetes‐associated pathological signals in TA and AA with extraneous noise, particularly hemodynamic interference in the aortic arch and structural variation in the iliac bifurcation.

We acknowledge that proteomic measurements might show substantial variability. Therefore, we implemented several measures to strengthen robustness despite the group size. First, we applied LCM to enrich vascular tissue and reduce contamination, improving reproducibility relative to whole‐tissue bulk homogenates. Second, all samples were processed and analyzed in a single experimental batch using identical preparation and LC‐MS/MS settings to minimize technical variation and batch effects. Third, our study design was paired and multidimensional (TA vs. AA within the same animal, and diabetes vs. control comparisons within region), which increases statistical power by leveraging within‐animal controls and consistent anatomical matching. Furthermore, the proteomic findings were not interpreted in isolation, where key candidates and pathways were validated using orthogonal approaches (e.g., RT‐PCR), supporting the biological reproducibility of the principal conclusions. Consistently, our PCA analyses demonstrate that most samples do not exhibit substantial within‐group variability.

### 3.3 Translational Relevance and Atlas Utility

Our proteomic analysis successfully identified novel protein and pathway signatures in diabetic vasculature. These newly identified novel markers were mostly found in human TA, highlighting clinical relevance. By integrating proteomic and single‐cell transcriptomic analyses, we identified distinct candidate markers specific to TA and AA in health and diabetes, and achieved the mapping of their distribution patterns across major vascular cell types. However, the mechanistic roles of these markers in either exacerbating or mitigating endothelial dysfunction still require extensive future study. Pathway dysregulation differed substantially between TA and AA in health and diabetes, suggesting disease‐specific spatial regulatory mechanisms. Therefore, distinct disease contexts necessitate separate investigation to delineate region‐specific pathophysiological mechanisms.

### 3.4 Spatially Dysregulated TRs

We identified novel TRs (e.g., Dpep1) as potential mediators of diabetic vasculopathy, offering new mechanistic insights. Furthermore, we consolidate established TRs with known roles in vasoregulation (e.g., Hdac2, Smad1, Stat1), by exploring their spatial dysregulation patterns in diabetic arteries. However, the limited sensitivity of snRNA‐seq means that low read counts for a gene can be misleading, potentially masking its actual expression abundance. For instance, since DPEP1 is a membrane protein, snRNA‐seq, which primarily captures the nuclear transcriptome, might severely underestimate DPEP1 levels by omitting the expression of mature DPEP1 transcripts in cytosol. Importantly, regionally confined upregulation inducing global vascular changes demonstrates the power of spatial proteomics to uncover hidden drivers (e.g., Dpep1) of diabetic vasculopathy.

### 3.5 DPEP1 as a Mechanistic Mediator of Neutrophilic Vascular Inflammation

Our study extended the functional role of endothelial DPEP1 in mediating diabetic vascular inflammation and endothelial dysfunction, beyond its established involvement in renal, pulmonary, and hepatic inflammation. We revealed a novel mechanism whereby DPEP1 aggravates diabetic endothelial dysfunction by initiating neutrophilic vascular inflammation, aligning with its established functions in pulmonary and hepatic ECs [31]. In addition to its catalytic role, we consolidated the role of DPEP1 as a neutrophil regulatory receptor with broader pathological relevance. Moreover, we extended the pathogenesis of diabetic vasculopathy by incorporating DPEP1 as a key amplifier in the diabetes‐LPS‐neutrophilic inflammation cascade, providing deeper mechanistic insight.

Our work identified vascular DPEP1 as a druggable target and extended the therapeutic utility of FDA‐approved drugs like cilastatin to the vasculature. Crucially, cilastatin's reversal of diabetic vascular damage was dissociated from systemic metabolic effects, implying its potential for vascular‐specific protection. However, we cannot exclude the possibility that cilastatin's vascular benefits arise, at least partially, through renal modulation, given the bidirectional link between diabetic nephropathy and vascular injury. However, although pharmacological inhibition and endothelium‐specific knockdown support Dpep1 as a functional mediator of endothelial dysfunction in diabetes, future gain‐of‐function experiments are warranted to test the necessity and sufficiency of Dpep1 in driving diabetic vasculopathy. Collectively, LPS, hyperglycemia, and cilastatin exert systemic effects on modulating vascular DPEP1 expression/activity, implying that the differential DPEP1 expression (TA > AA) arises from intrinsic vascular mechanisms beyond these stimuli.

### 3.6 Mechanosensitive Regulation: The LSS/GR/DPEP1 Axis

Importantly, DPEP1 exhibits multifaceted functions across pathologies, acting as a catalytic enzyme, a surface receptor, and a potential transcriptional modulator. However, we did not explore the role of DPEP1 as a TR within diabetic vasculopathy, given its potential involvement in transcriptional regulation in cancer pathology [39]. Our findings underscore the critical concept that the regulation of key proteins, such as shear‐sensitive proteins, arises from a complex interplay between hemodynamic forces and metabolic stimuli. Particularly, we further identified DPEP1 as a novel shear‐sensitive protein, expanding its pleiotropic roles in ECs, where its differential regulation by varied shear stress intensities might explain its site‐specific upregulation in diabetic aortas. Mechanistically, we delineated that an LSS/GR/DPEP1 axis in ECs, aligning with prior indications that Dpep1 is a potential downstream target of GR in adipogenesis and renal metabolism [35, 40].

### 3.7 Context‐Dependent GR/DPEP1 Signaling

Our findings also illuminate a nuanced reality: although GR nuclear translocation, LSS, and dexamethasone are broadly associated with anti‐inflammatory actions, this does not preclude the possibility that they concurrently engage pro‐inflammatory components (e.g., DPEP1). However, the ultimate anti‐inflammatory outcome suggests the broader anti‐inflammatory program they activate exerts a dominant and overriding effect, which compensates for the harmful effects of pro‐inflammatory components. Particularly, stable LSS accounts for the constitutive DPEP1 upregulation in TA, alongside other anti‐inflammatory and vasoprotective cascades. Therefore, the benefits of LSS potentially outweigh the detrimental effects arising from DPEP1.

An alternative explanation centers on hierarchical signaling priorities. The identified GR/DPEP1 axis alone might be insufficient to drive the full spectrum of neutrophilic vascular inflammation, due to the pleiotropic nature of LSS and dexamethasone. The net phenotypic outcome represents the integrated output of a complex network. The dichotomous effects of acute versus chronic dexamethasone treatment on the vasculature underscore that the timing and duration of an intervention are decisive factors in shaping the net biological response.

### 3.8 Limitations

The study is subject to certain limitations. First, the exclusive use of male mice precludes an investigation into sex‐specific effects. Second, our spatial proteomic analysis is potentially limited by modest cohort size. In addition, one diabetic TA sample (db/db TA1) was excluded as an outlier, which further reduced statistical power and may limit sensitivity for detecting subtle changes. Nevertheless, the principal trends and key candidates were supported by independent orthogonal validation (e.g., RT‐PCR), mitigating the impact of this exclusion on the overall conclusions. Third, since db/db mice incompletely capture the heterogeneity and comorbidities of human Type 2 diabetes, further validation in additional diabetic models (e.g., diet‐induced obesity or atherosclerosis‐prone models) and region‐matched human vascular samples are needed.

Fourth, our study is constrained by the extremely limited availability of human aortic tissue samples, where procuring specific regions of human aortic samples from diabetic donors is exceptionally challenging. Furthermore, due to the limited availability of sequencing datasets on human TA and AA under diabetic conditions, we were able to identify only one snRNA‐seq dataset of human TA from a nondiabetic background [27]. However, our correlation with human snRNA‐seq data, while informative, is inherently limited as the technique primarily captures the nuclear transcriptome, thereby underrepresenting gene expression signatures from other subcellular compartments.

### 3.9 Conclusions and Outlook

This work establishes a foundational spatially resolved proteomic atlas of diabetic aorta, uncovering region‐specific protein and pathway signatures that are easily masked by conventional bulk analyses. Our multidimensional comparative framework uniquely identified the GR/DPEP1 axis as a novel and mechanosensitive signaling pathway mediating diabetic vasculopathy. We demonstrate that DPEP1, upregulated specifically in diabetic TA, functions as a critical driver of neutrophilic inflammation and endothelial dysfunction. This work expands DPEP1's role into vascular pathology by linking diabetic cues and LSS to GR/DPEP1 activation (Figure S55), and provides a rich resource of region‐specific markers, opening new avenues for diagnosing and treating diabetic vascular complications. The spatiotemporal dimension of intervention in vascular therapy shall not be overlooked.

## 4 Materials and Methods

### 4.1 Animal Studies and Ethical Compliance

All animal procedures were approved by the Animal Research Ethics Sub‐Committee of City University of Hong Kong (Approval No. AN‐STA‐00000132) and conducted in accordance with the NIH Guide for the Care and Use of Laboratory Animals and the ARRIVE guidelines. Male db/m^+^, db/db, and C57BL/6 mice (10 weeks old) were provided by the Laboratory Animal Research Unit at City University of Hong Kong. Mice were housed in individually ventilated cages under controlled conditions (temperature 23 ± 1°C, humidity 55 ± 5%, and a 12‐h light/12‐h dark cycle) within a specific pathogen‐free facility. Food and water were provided ad libitum. Randomization was applied prior to all experiments.

Some db/m^+^ and db/db mice were intravenously injected with saline or cilastatin (30 mg/kg; Sigma‐Aldrich) twice a week for 4 consecutive weeks. For AAV‐mediated Dpep1 knockdown in vivo, some db/m^+^ and db/db mice were intravenously injected with AAV1‐scramble or AAV1‐ICAM2‐shDpep1 (2 × 10^11^ vg per mouse), generated by Vigene Biosciences (Maryland, USA). Some db/m^+^ and db/db mice were intraperitoneally injected with saline or dexamethasone (5 mg/kg; Sigma‐Aldrich) and were sacrificed at different time points: 1 h, 24 h, or after 4 weeks of daily injections.

### 4.2 Frozen Section

Descending TA (above diaphragm) and AA (below diaphragm) of db/m^+^ and db/db mice (*n* = 4 individual mice per group) were dissected free of adhering connective tissues in ice‐cold PBS. The central ∼5 mm segments of both descending TA and AA were harvested to minimize the edge effects from adjacent vascular regions, thereby eliminating interference from branch ostia and peripheral microvascular networks. After excess PBS on the tissue surface was removed, the aortic segments were soaked in liquid nitrogen pre‐cooled isopentane for 1 min and were then embedded in optimum cutting temperature (OCT) compound (Sakura Finetek, Flemingweg, Holland), followed by snap‐frozen. The OCT‐embedded segments were sectioned at 10 µm (cross section) on a CM1950 cryostat (Leica, Wetzlar, Germany) and mounted onto PEN membrane‐coated glass slides (415190‐9041‐000; Zeiss, Oberkochen, Germany).

### 4.3 Laser‐Capture Microdissection

LCM was performed on LCM System PALM (Zeiss). The LCM system was initially turned on for 15 min to stabilize laser energy. The microscope and laser settings were set up as following: Zoom: 20x; cut energy: 48; focus: 62; catapulting energy: 20; focus: 70; cycle number: 1; cut speed: 5. The dried H&E‐stained slide of aortic section was put onto the slide adapter of the LCM microscope. The regions of interest in the section were marked with LCM marker pen, microdissected using the above settings, and collected by microtubes (415190‐9201‐000, Zeiss). The microdissected samples were stored at −80°C for later digestion for MS‐based proteomics.

### 4.4 Liquid Chromatography–Tandem MS and Data Processing

The tryptic peptides were dissolved in solvent A and were directly loaded onto a home‐made reverse‐phase analytical column (15 cm length × 100 µm internal diameter), using a Vanquish Neo UPLC system (Thermo Fisher Scientific). The mobile phase comprised Solvent A (0.1% formic acid in water) and Solvent B (0.1% formic acid, 80% acetonitrile in water), with chromatographic separation achieved through the following gradient program at a constant flow rate (200 nL/min): 0–1.6 min, 4%–22.5% B; 1.6–2.0 min, 22.5%–35% B; 2.0–2.1 min, 35.0%–35.1% B; 2.1–2.3 min, 35.1% B; 2.3–9.2 min, 35.1%–35.2% B; 9.2–9.6 min, 35.2%–55.0% B; 9.6–10.1 min, 55.0%–99.0% B; 10.1–12.0 min, 99% B. The separated peptides were ionized using a nano‐electrospray ion source at 1900 V and were analyzed on an Orbitrap Astral mass spectrometer (Thermo Fisher Scientific). Full‐scan MS spectra (400–800 *m/z*) were acquired in the Orbitrap mass analyzer at a resolution of 240,000, while tandem MS (MS/MS) spectra were obtained in the Astral analyzer with a resolution of 80,000, using a fixed first mass of 150.0 *m/z*. Higher‐energy collisional dissociation was performed at a normalized collision energy (25%). Automatic gain control was set to 800% with a maximum ion injection time (15 ms) to optimize sensitivity and dynamic range.

Data‐independent acquisition (DIA) data were analyzed using the DIA‐NN computational platform (v1.8). Tandem mass spectra were queried against the Mus_musculus_10090_SP_20231220.fasta (17,191 entries), concatenated with reverse decoy database. Trypsin/P was designated as the proteolytic enzyme with allowance for up to one missed cleavage. Fixed modifications included N‐terminal methionine excision and cysteine carbamidomethylation. Peptide and protein identifications were filtered at a global false discovery rate threshold of 1%. Further data filtering on the database search results was performed to enhance analytic quality by ensuring that the identified proteins must contain at least one peptide.

### 4.5 In Vitro Hemodynamic Simulation

High LSS (15 dyn/cm^2^) and low LSS (8 dyn/cm^2^) were applied using an ibidi flow system (Gräfelfing, Germany), coupled with custom‐built flow chambers (Figure 8C). HAECs (5 × 10^5^ cells) were seeded onto fibronectin (50 µg/mL, 24 h)‐coated glass slides (75 mm × 38 mm; Corning, USA). Following a 16‐h attachment, cells were incubated in EGM containing 2% FBS. Slides were then mounted into flow chambers and connected to the ibidi system, followed by LSS exposure for 1 h or 24 h, in the presence or absence of LPS (1 µg/mL).

### 4.6 Statistical Analysis

Results are expressed as mean ± SD. Statistical analyses were conducted with GraphPad Prism (Version 10.0). Significance between two groups was determined using two‐tailed unpaired *t*‐tests and nonparametric Mann–Whitney tests. Comparisons among multiple groups employed Brown–Forsythe and Welch ANOVA tests (unpaired *t* with Welch's correction). A *p* value < 0.05 was considered statistically significant.

Detailed Materials and Methods are available in the online‐only Supporting Information.

## Author Contributions

**Chak Kwong Cheng**: conceptualization, data curation, formal analysis, funding acquisition, investigation, methodology, project administration, supervision, validation, visualization, writing – original draft, writing – review and editing. **Shuhui Meng**: formal analysis, investigation, methodology. **Teng Li**: data curation, formal analysis, investigation, methodology, software. **Huanyu Ding**: investigation. **Minchun Jiang**: investigation. **Zizhao Tian**: formal analysis, investigation. **Chi‐Fai Ng**: resources, writing – review and editing. **Yin Xia**: resources, writing – review and editing. **Stefan Offermanns**: resources, writing – review and editing. **Yu Huang**: funding acquisition, project administration, resources, supervision, writing – review and editing. All authors have read and approved the final manuscript.

## Funding

This work was supported by the Research Grants Council of the Hong Kong Special Administrative Region, China (T12‐101/23‐N, RGC‐SRFS2021‐4S04) and the City University of Hong Kong Start‐Up Fund. This work was also substantially supported by a fellowship award from the Research Grants Council of the Hong Kong Special Administrative Region, China (project No. CityU PDFS2223‐1S01) and the fellowship scheme funded by William G. Kerckhoff Foundation.

## Ethics Statement

All animal procedures were approved by the Animal Research Ethics Sub‐Committee of City University of Hong Kong (Approval No. AN‐STA‐00000132) and conducted in accordance with the NIH Guide for the Care and Use of Laboratory Animals and the ARRIVE guidelines. The procedures involving human individuals were conducted in accordance with the Declaration of Helsinki and were approved by the Clinical Research Ethics Committee of The Chinese University of Hong Kong (CUHK; Approval No.: 2014.468 and 2018.055). All participants signed informed consent prior to their inclusion in the study.

## Conflicts of Interest

The authors declare no conflicts of interest.

## Supporting information




**Figure S1**. Diabetic phenotype confirmation and endothelium‐independent aortic relaxation. (A) Fasting glucose levels of diabetic mice. (B) endothelium‐independent relaxations of aortas from diabetic mouse aortas and corresponding AUC analysis. *n* = 5 per group. Data are presented as mean ± SD. Brown‐Forsythe and Welch ANOVA, and unpaired t with Welch's correction. AA, abdominal aorta; AUC, area under the curve; Phe, phenylephrine; SNP, sodium nitroprusside; TA, descending thoracic aorta.
**Figure S2**. Quantification on Western blotting results of AMPK expression. *n* = 4 per group. Data are presented as mean ± SD. Brown‐Forsythe and Welch ANOVA, and unpaired t with Welch's correction. AA, abdominal aorta; AMPK, AMP‐activated protein kinase; TA, descending thoracic aorta.
**Figure S3**. RT‐PCR on inflammatory markers and ROS‐related genes of mouse thoracic and abdominal aortas. *n* = 5 per group. Data are presented as mean ± SD. *p < 0.05 (Brown‐Forsythe and Welch ANOVA, and unpaired t with Welch's correction). AA, abdominal aorta; TA, descending thoracic aorta.
**Figure S4**. Proteome coverage of mouse aortic samples. (A) Number of peptides and proteins detected by LCM‐based MS. (B) Number of proteins detected in different groups of mouse aortic segments. AA, abdominal aorta; LCM, laser‐capture microdissection; MS, mass spectrometry; TA, thoracic aorta.
**Figure S5**. Heatmap showing distinct clustering between the two combined groups. *n* = 8 for db/m^+^ aorta; *n* = 7 for db/db aorta. AA, abdominal aorta; TA, descending thoracic aorta.
**Figure S6**. Number of upregulated and downregulated DEPs in db/db aorta vs db/m^+^ aorta. DEP, differentially expressed protein.
**Figure S7**. RT‐PCR on candidate markers in db/m^+^ aorta and db/db aorta. *n* = 5 per group. Data are presented as mean ± SD. *p < 0.05 (unpaired *t*‐tests and nonparametric Mann‐Whitney tests).
**Figure S8**. Cellular distribution and expression of selected marker genes from mouse aorta in human thoracic aorta based on snRNA‐seq data. (A) UMAP visualization of 50133 individual nuclei from thoracic aortas of 3 individuals. (B) UMAP plots showing the distribution and violin plots showing the expression of selected marker genes in different vascular cell types. snRNA‐seq, single‐nucleus RNA sequencing; UMAP, uniform manifold approximation and projection.
**Figure S9**. Positively and negatively enriched GO terms in db/db aorta vs db/m^+^ aorta, ranked by log2 fold enrichment. Dot plots display significantly enriched GO terms (FDR < 0.05) across three ontologies, including (A) CC, (B) BP, and (C) MF. BP, biological process; CC, cellular component; FDR, false discovery rate; GO, Gene Ontology; MF, molecular function.
**Figure S10**. Chord diagram linking significantly enriched KEGG pathways to DEPs, ranked by Log_2_FC, in db/db aorta vs db/m^+^ aorta. DEP, differentially expressed protein; FC, fold change; KEGG, Kyoto Encyclopedia of Genes and Genomes.
**Figure S11**. Heatmaps showing distinct clustering between TA and AA of (A) db/m^+^ and (B) db/db mice. *n* = 4 for db/m^+^ TA; *n* = 4 for db/m^+^ AA; *n* = 3 for db/db TA; *n* = 4 for db/db AA. AA, abdominal aorta; TA, descending thoracic aorta.
**Figure S12**. Number of upregulated and downregulated DEPs in TA and AA of (A) db/m^+^ mice and (B) db/db mice. AA, abdominal aorta; DEP, differentially expressed protein; TA, descending thoracic aorta.
**Figure S13**. Nightingale rose diagram on subcellular localizations of upregulated and downregulated DEPs in TA and AA of (A) db/m^+^ aorta and (B) db/db aorta. AA, abdominal aorta; DEP, differentially expressed protein; TA, descending thoracic aorta.
**Figure S14**. Volcano plots on DEPs between TA and AA of (A) db/m^+^ mice and (B) db/db mice. Grey dotted lines: vertical at |Log_2_ FC| = 0.6 (∼1.5‐fold change) and horizontal at ‐log_10_ *p* = 1.3 (*p* < 0.05). AA, abdominal aorta; DEP, differentially expressed protein; TA, descending thoracic aorta.
**Figure S15**. RT‐PCR on candidate markers in TA and AA of (A) db/m^+^ mice and (B) db/db mice. *n* = 5 per group. Data are presented as mean ± SD. *p < 0.05 (unpaired *t*‐tests and nonparametric Mann‐Whitney tests). AA, abdominal aorta; TA, descending thoracic aorta.
**Figure S16**. Cellular distribution and expression of selected marker genes from mouse TA and AA in human thoracic aorta based on snRNA‐seq data. (A) UMAP visualization of 50133 individual nuclei from thoracic aortas of 3 individuals. (B) UMAP plots showing the distribution and violin plots showing the expression of selected marker genes in different vascular cell types. AA, abdominal aorta; snRNA‐seq, single‐nucleus RNA sequencing; TA, descending thoracic aorta; UMAP, uniform manifold approximation and projection.
**Figure S17**. Positively and negatively enriched GO terms in db/m^+^ AA vs db/m^+^ TA, ranked by log2 fold enrichment. Dot plots display significantly enriched GO terms (FDR < 0.05) across three ontologies, including (A) CC, (B) BP, and (C) MF. AA, abdominal aorta; BP, biological process; CC, cellular component; FDR, false discovery rate; GO, Gene Ontology; MF, molecular function; TA, descending thoracic aorta.
**Figure S18**. Positively and negatively enriched GO terms in db/db AA vs db/db TA, ranked by log2 fold enrichment. Dot plots display significantly enriched GO terms (FDR < 0.05) across three ontologies, including (A) CC, (B) BP, and (C) MF. AA, abdominal aorta; BP, biological process; CC, cellular component; FDR, false discovery rate; GO, Gene Ontology; MF, molecular function; TA, descending thoracic aorta.
**Figure S19**. Chord diagrams linking significantly enriched KEGG pathways to DEPs, ranked by Log_2_FC, in AA vs TA of (A) db/m^+^ mice and (B) db/db mice. AA, abdominal aorta; DEP, differentially expressed protein; FC, fold change; KEGG, Kyoto Encyclopedia of Genes and Genomes; TA, descending thoracic aorta.
**Figure S20**. Heatmaps showing distinct clustering of (A) TA between db/m^+^ and db/db mice, and (B) AA between db/m^+^ and db/db mice. *n* = 4 for db/m^+^ TA; *n* = 3 for db/db TA; *n* = 4 for db/m^+^ AA; *n* = 4 for db/db AA. AA, abdominal aorta; TA, descending thoracic aorta.
**Figure S21**. Number of upregulated and downregulated DEPs in (A) TA between db/m^+^ mice and db/db mice, and (B) AA between db/m^+^ mice and db/db mice. AA, abdominal aorta; DEP, differentially expressed protein; TA, descending thoracic aorta.
**Figure S22**. Nightingale rose diagram on subcellular localizations of upregulated and downregulated DEPs in (A) db/db TA vs db/m^+^ TA, and (B) db/db AA vs db/m^+^ AA. AA, abdominal aorta; DEP, differentially expressed protein; TA, descending thoracic aorta.
**Figure S23**. Volcano plots on DEPs in (A) TA between db/m^+^ and db/db mice, and (B) AA between db/m^+^ and db/db mice. Grey dotted lines: vertical at |Log_2_ FC| = 0.6 (∼1.5‐fold change) and horizontal at ‐log_10_ *p* = 1.3 (*p* < 0.05). AA, abdominal aorta; DEP, differentially expressed protein; TA, descending thoracic aorta.
**Figure S24**. RT‐PCR on candidate markers in (A) TA of db/db vs db/m^+^ mice, and (B) AA of db/db vs db/m^+^ mice. *n* = 5 per group. Data are presented as mean ± SD. *p < 0.05 (unpaired *t*‐tests and nonparametric Mann‐Whitney tests). AA, abdominal aorta; TA, descending thoracic aorta.
**Figure S25**. Cellular distribution and expression of selected marker genes from mouse TA and AA in human thoracic aorta based on snRNA‐seq data. (A) UMAP visualization of 50133 individual nuclei from thoracic aortas of 3 individuals. (B) UMAP plots showing the distribution and violin plots showing the expression of selected marker genes in different vascular cell types. AA, abdominal aorta; snRNA‐seq, single‐nucleus RNA sequencing; TA, descending thoracic aorta; UMAP, uniform manifold approximation and projection.
**Figure S26**. Positively and negatively enriched GO terms in db/db TA vs db/m^+^ TA, ranked by log2 fold enrichment. Dot plots display significantly enriched GO terms (FDR < 0.05) across three ontologies, including (A) CC, (B) BP, and (C) MF. BP, biological process; CC, cellular component; FDR, false discovery rate; GO, Gene Ontology; MF, molecular function; TA, descending thoracic aorta.
**Figure S27**. Positively and negatively enriched GO terms in db/db AA vs db/m^+^ AA, ranked by log2 fold enrichment. Dot plots display significantly enriched GO terms (FDR < 0.05) across three ontologies, including (A) CC, (B) BP, and (C) MF. AA, abdominal aorta; BP, biological process; CC, cellular component; FDR, false discovery rate; GO, Gene Ontology; MF, molecular function.
**Figure S28**. Chord diagrams linking significantly enriched KEGG pathways to DEPs, ranked by Log_2_FC, in (A) db/db TA vs db/m^+^ TA, and (B) db/db AA vs db/m^+^ AA. AA, abdominal aorta; DEP, differentially expressed protein; FC, fold change; KEGG, Kyoto Encyclopedia of Genes and Genomes; TA, descending thoracic aorta.
**Figure S29**. Heatmap showing distinct clustering of TRs and TR targets between the four aortic groups. *n* = 4 for db/m^+^ TA; *n* = 3 for db/db TA; *n* = 4 for db/m^+^ AA; *n* = 4 for db/db AA. AA, abdominal aorta; TA, descending thoracic aorta; TR, transcriptional regulator.
**Figure S30**. Heatmap depicting the relative protein expression of the 18 differentially expressed TRs among the four aortic groups. *n* = 4 for db/m^+^ TA; *n* = 4 for db/m^+^ AA; *n* = 3 for db/db TA; *n* = 4 for db/db AA. AA, abdominal aorta; TA, descending thoracic aorta; TR, transcriptional regulator.
**Figure S31**. RT‐PCR on 18 differentially expressed TRs in TA and AA of non‐diabetic and diabetic mice among different group comparisons, including (a) db/m^+^ vs db/db, (b) db/m^+^ TA vs db/m^+^ AA, (c) db/db TA vs db/db AA, (d) db/m^+^ TA vs db/db TA, and (e) db/m^+^ AA vs db/db AA. *n* = 5 per group. Data are presented as mean ± SD. *p < 0.05 (unpaired *t*‐tests and nonparametric Mann‐Whitney tests). AA, abdominal aorta; TA, descending thoracic aorta; TR, transcriptional regulator.
**Figure S32**. Cellular distribution of the 18 differentially expressed TRs from mouse TA and AA in human thoracic aorta based on snRNA‐seq data. (A) UMAP visualization of 50133 individual nuclei from thoracic aortas of 3 individuals. (B) UMAP plots showing the distribution of the 18 TRs in different vascular cell types. AA, abdominal aorta; snRNA‐seq, single‐nucleus RNA sequencing; TA, descending thoracic aorta; TR, transcriptional regulator; UMAP, uniform manifold approximation and projection.
**Figure S33**. Violin plots showing the expression of the 18 TRs in different vascular cell types from mouse TA and AA in human thoracic aorta based on snRNA‐seq data. AA, abdominal aorta; snRNA‐seq, single‐nucleus RNA sequencing; TA, descending thoracic aorta; TR, transcriptional regulator.
**Figure S35**. Effects of 4‐week cilastatin treatment on body weights and blood glucose levels of non‐diabetic and diabetic mice. (A) Body weights, (B) fasting glucose levels, (C) OGTT, and (D) serum LPS levels of db/m^+^ and db/db mice after 4‐week cilastatin treatment (*n* = 5 per group). Data are presented as mean ± SD. *p < 0.05 (Brown‐Forsythe and Welch ANOVA, and unpaired t with Welch's correction). LPS, lipopolysaccharide; OGTT, oral glucose tolerance test.
**Figure S36**. Quantification on Western blotting results of Dpep1 expression in aortic segments of cilastatin‐treated db/m^+^ and db/db mice. *n* = 4 per group. Data are presented as mean ± SD. *p < 0.05 (Brown‐Forsythe and Welch ANOVA, and unpaired t with Welch's correction). AA, abdominal aorta; Dpep1, dipeptidase 1; TA, descending thoracic aorta.
**Figure S37**. RT‐PCR on inflammatory markers of mouse aortas after chronic cilastatin treatment. *n* = 5 per group. Data are presented as mean ± SD. *p < 0.05 (Brown‐Forsythe and Welch ANOVA, and unpaired t with Welch's correction).
**Figure S38**. Endothelium‐independent relaxations of diabetic mouse aortas upon chronic cilastatin treatment. *n* = 5 per group. Data are presented as mean ± SD. Brown‐Forsythe and Welch ANOVA, and unpaired t with Welch's correction. Phe, phenylephrine; SNP, sodium nitroprusside.
**Figure S39**. Western blotting on Dpep1 expression in thoracic and abdominal aortas of non‐diabetic and diabetic mice upon injection of AAV‐shDpep1. *n* = 4 per group. Data are presented as mean ± SD. *p < 0.05 (Brown‐Forsythe and Welch ANOVA, and unpaired t with Welch's correction). AA, abdominal aorta; AAV, adeno‐associated virus; Dpep1, dipeptidase 1; TA, descending thoracic aorta.
**Figure S40**. Effects of AAV‐mediated Dpep1 knockdown on body weights and blood glucose levels of non‐diabetic and diabetic mice. (A) Body weights, (B) fasting glucose levels, and (C) OGTT of db/m^+^ and db/db mice after injection of AAV‐shDpep1 (*n* = 5 per group). Data are presented as mean ± SD. *p < 0.05 (Brown‐Forsythe and Welch ANOVA, and unpaired t with Welch's correction). AAV, adeno‐associated virus; Dpep1, dipeptidase 1; OGTT, oral glucose tolerance test.
**Figure S41**. RT‐PCR on inflammatory markers of mouse aortas upon AAV‐mediated Dpep1 knockdown. *n* = 5 per group. Data are presented as mean ± SD. *p < 0.05 (Brown‐Forsythe and Welch ANOVA, and unpaired t with Welch's correction). AAV, adeno‐associated virus; Dpep1, dipeptidase 1.
**Figure S42**. Endothelium‐independent relaxations of diabetic mouse aortas upon AAV‐mediated Dpep1 knockdown. *n* = 5 per group. Data are presented as mean ± SD. Brown‐Forsythe and Welch ANOVA, and unpaired t with Welch's correction. AAV, adeno‐associated virus; Dpep1, dipeptidase 1; Phe, phenylephrine; SNP, sodium nitroprusside.
**Figure S43**. Western blotting on GR expression in nuclear/cytoplasmic fractions of HAECs upon GR knockdown and laminar flow exposure. *n* = 4 per group. Data are presented as mean ± SD. Brown‐Forsythe and Welch ANOVA, and unpaired t with Welch's correction. GR, glucocorticoid receptor; HAEC, human aortic endothelial cell; LSS, laminar shear stress; siScr, scrambled siRNA.
**Figure S44**. Western blotting on GR expression in nuclear/cytoplasmic fractions of HAECs upon GR knockdown and dexamethasone treatment. *n* = 4 per group. Data are presented as mean ± SD. Brown‐Forsythe and Welch ANOVA, and unpaired t with Welch's correction. Dex, dexamethasone; GR, glucocorticoid receptor; HAEC, human aortic endothelial cell; siScr, scrambled siRNA.
**Figure S45**. Effects of acute dexamethasone treatment on body weights and blood glucose levels of non‐diabetic and diabetic mice. (A). Body weights, (B) fasting glucose levels, and (C) OGTT of db/m^+^ and db/db mice 24 hr after dexamethasone injection (*n* = 5 per group). Data are presented as mean ± SD. *p < 0.05 (Brown‐Forsythe and Welch ANOVA, and unpaired t with Welch's correction). Dex, dexamethasone; OGTT, oral glucose tolerance test.
**Figure S46**. Effects of chronic dexamethasone treatment on body weights and blood glucose levels of non‐diabetic and diabetic mice. (A) Body weights, (B) fasting glucose levels, and (C) OGTT of db/m^+^ and db/db mice after 4‐week dexamethasone treatment (*n* = 5 per group). Data are presented as mean ± SD. *p < 0.05 (Brown‐Forsythe and Welch ANOVA, and unpaired t with Welch's correction). Dex, dexamethasone; OGTT, oral glucose tolerance test.
**Figure S47**. Western blotting on GR expression in nuclear/cytoplasmic fractions of non‐diabetic and diabetic mouse aortas upon acute dexamethasone treatment. *n* = 4 per group. Data are presented as mean ± SD. Brown‐Forsythe and Welch ANOVA, and unpaired t with Welch's correction. AA, abdominal aorta; Dex, dexamethasone; GR, glucocorticoid receptor; TA, descending thoracic aorta.
**Figure S48**. Western blotting on GR expression in nuclear/cytoplasmic fractions of non‐diabetic and diabetic mouse aortas upon chronic dexamethasone treatment. *n* = 4 per group. Data are presented as mean ± SD. Brown‐Forsythe and Welch ANOVA, and unpaired t with Welch's correction. AA, abdominal aorta; Dex, dexamethasone; GR, glucocorticoid receptor; TA, descending thoracic aorta.
**Figure S49**. RT‐PCR on inflammatory markers of mouse aortas upon acute dexamethasone treatment. *n* = 5 per group. Data are presented as mean ± SD. *p < 0.05 (Brown‐Forsythe and Welch ANOVA, and unpaired t with Welch's correction). Dex, dexamethasone.
**Figure S50**. RT‐PCR on inflammatory markers of mouse aortas upon chronic dexamethasone treatment. *n* = 5 per group. Data are presented as mean ± SD. *p < 0.05 (Brown‐Forsythe and Welch ANOVA, and unpaired t with Welch's correction). Dex, dexamethasone.
**Figure S51**. Western blotting on phosphorylated eNOS and total eNOS in aortas of non‐diabetic and diabetic mice after acute dexamethasone treatment. *n* = 4 per group. Data are presented as mean ± SD. Brown‐Forsythe and Welch ANOVA, and unpaired t with Welch's correction. Dex, dexamethasone; eNOS, endothelial nitric oxide synthase.
**Figure S52**. Western blotting on phosphorylated eNOS and total eNOS in aortas of non‐diabetic and diabetic mice after chronic dexamethasone treatment. *n* = 4 per group. Data are presented as mean ± SD. Brown‐Forsythe and Welch ANOVA, and unpaired t with Welch's correction. Dex, dexamethasone; eNOS, endothelial nitric oxide synthase.
**Figure S53**. Endothelium‐independent relaxations of diabetic mouse aortas upon acute dexamethasone treatment. *n* = 5 per group. Data are presented as mean ± SD. Brown‐Forsythe and Welch ANOVA, and unpaired t with Welch's correction. Dex dexamethasone; Phe, phenylephrine; SNP, sodium nitroprusside.
**Figure S54**. Endothelium‐independent relaxations of diabetic mouse aortas upon chronic dexamethasone treatment. *n* = 5 per group. Data are presented as mean ± SD. Brown‐Forsythe and Welch ANOVA, and unpaired t with Welch's correction. Dex dexamethasone; Phe, phenylephrine; SNP, sodium nitroprusside.
**Figure S55**. Spatially differential regulation of the GR/DPEP1 axis by hemodynamic shear stress and diabetic conditions. In diabetic TA, higher LSS magnitude and metabolic stressors co‐activate the GR/DPEP1 axis in mediating neutrophilic inflammation and diabetic vasculopathy. In diabetic AA, lower LSS magnitude corresponds to less pronounced GR nuclear translocation and DPEP1 upregulation. Cilastatin treatment ameliorates the features of diabetic vasculopathy through suppressing DPEP1 activity. Though inducing GR nuclear translocation, dexamethasone exerts pleiotropic and time‐dependent effects on diabetic vasculature, being acutely suppressive but chronically promotive to diabetic vasculopathy. AA, abdominal aorta; Dex, dexamethasone; DPEP1, dipeptidase 1; EC, endothelial cell; GR, glucocorticoid receptor; LPS, lipopolysaccharide; LSS, laminar shear stress; TA, descending thoracic aorta.
**Table S1**. List of upregulated and downregulated DEPs in db/db aorta vs db/m^+^ aorta.
**Table S2**. List of upregulated and downregulated DEPs in db/m^+^ AA vs db/m^+^ TA.
**Table S3**. List of upregulated and downregulated DEPs in db/db AA vs db/db TA.
**Table S4**. List of upregulated and downregulated DEPs in db/db TA vs db/m^+^ TA.
**Table S5**. List of upregulated and downregulated DEPs in db/db AA vs db/m^+^ AA.
**Table S6**. Antibodies used in this study.
**Table S7**. RT‐PCR primers for detection of mouse genes.
**Table S8**. Demographic characteristics of the included patients.

## Data Availability

We declare that the data supporting the findings of this study are available within this manuscript and its Supporting Information files. The proteomic data discussed in this publication have been deposited in PRIDE and are accessible through the accession number PXD067688.
