# Supplementary material for: Spatially Characterized Aortic Proteome Reveals Novel Regional Signatures and Glucocorticoid Receptor/Dipeptidase 1 Axis in Diabetic Vasculopathy
Source: MedComm (2020). 2026 Mar 30;7(4):e70714. doi: 10.1002/mco2.70714 (PMC13042678; doi:10.1002/mco2.70714)
Supplement: Supplementary file 1 — Figure S1. Diabetic phenotype confirmation and endothelium‐independent aortic relaxation. (A) Fasting glucose levels of diabetic mice. (B) endothelium‐independent relaxations of aortas from diabetic mouse aortas and corresponding AUC analysis. n = 5 per group. Data are presented as mean ± SD. Brown‐Forsythe and Welch ANOVA, and unpaired t with Welch's correction. AA, abdominal aorta; AUC, area under the curve; Phe, phenylephrine; SNP, sodium nitroprusside; TA, descending thoracic aorta. Figure S2. Quantification on Western blotting results of AMPK expression. n = 4 per group. Data are presented as mean ± SD. Brown‐Forsythe and Welch ANOVA, and unpaired t with Welch's correction. AA, abdominal aorta; AMPK, AMP‐activated protein kinase; TA, descending thoracic aorta. Figure S3. RT‐PCR on inflammatory markers and ROS‐related genes of mouse thoracic and abdominal aortas. n = 5 per group. Data are presented as mean ± SD. *p < 0.05 (Brown‐Forsythe and Welch ANOVA, and unpaired t with Welch's correction). AA, abdominal aorta; TA, descending thoracic aorta. Figure S4. Proteome coverage of mouse aortic samples. (A) Number of peptides and proteins detected by LCM‐based MS. (B) Number of proteins detected in different groups of mouse aortic segments. AA, abdominal aorta; LCM, laser‐capture microdissection; MS, mass spectrometry; TA, thoracic aorta. Figure S5. Heatmap showing distinct clustering between the two combined groups. n = 8 for db/m+ aorta; n = 7 for db/db aorta. AA, abdominal aorta; TA, descending thoracic aorta. Figure S6. Number of upregulated and downregulated DEPs in db/db aorta vs db/m+ aorta. DEP, differentially expressed protein. Figure S7. RT‐PCR on candidate markers in db/m+ aorta and db/db aorta. n = 5 per group. Data are presented as mean ± SD. *p < 0.05 (unpaired t‐tests and nonparametric Mann‐Whitney tests). Figure S8. Cellular distribution and expression of selected marker genes from mouse aorta in human thoracic aorta based on snRNA‐seq data. (A) UMAP [file MCO2-7-e70714-s001.docx]

**Spatially characterized aortic proteome reveals novel regional signatures and glucocorticoid receptor/dipeptidase 1 axis in diabetic vasculopathy**

*Running title: Vascular spatial heterogeneity in diabetes*

Chak Kwong Cheng^1,2,#,^*, Shuhui Meng^2,#^, Teng Li^3^, Huanyu Ding^4^, Minchun Jiang^4,5^, Zizhao Tian^2^, Chi-Fai Ng^6^, Yin Xia^4^, Stefan Offermanns^1^, Yu Huang^2,^*

^1^Department of Pharmacology, Max Planck Institute for Heart and Lung Research, Bad Nauheim 61231, Germany

^2^Department of Biomedical Sciences and Tung Biomedical Sciences Centre, City University of Hong Kong, Hong Kong 999077, China

^3^Department of Cell Biology & Institute of Biomedicine, College of Life Science and Technology, Jinan University, Guangzhou 510000, China

^4^School of Biomedical Sciences, Faculty of Medicine, The Chinese University of Hong Kong; Hong Kong 999077, China

^5^Department of Endocrinology, Guangdong Provincial People's Hospital (Guangdong Academy of Medical Sciences), Southern Medical University; Guangzhou 510000, China

^6^S.H. Ho Urology Centre, Department of Surgery, The Chinese University of Hong Kong, Hong Kong 999077, China

*Correspondence: Yu Huang ([yu.huang@cityu.edu.hk](mailto:yu.huang@cityu.edu.hk)) or Chak Kwong Cheng ([chak-kwong.cheng@mpi-bn.mpg.de](mailto:chak-kwong.cheng@mpi-bn.mpg.de))

^#^Chak Kwong Cheng and Shuhui Meng have contributed equally to this work.

**Part I. Methods and Materials**

*Animal studies and ethical compliance*

All animal procedures were approved by the Animal Research Ethics Sub-Committee of City University of Hong Kong (Approval No. AN-STA-00000132) and conducted in accordance with the NIH Guide for the Care and Use of Laboratory Animals and ARRIVE guidelines. Male db/m^+^, db/db, and C57BL/6 mice (10 weeks old) were provided by the Laboratory Animal Research Unit at City University of Hong Kong. Mice were housed in individually ventilated cages under controlled conditions (temperature 23 ± 1°C, humidity 55 ± 5%, and a 12-hr light/12-hr dark cycle) within a specific pathogen-free facility. Food and water were provided *ad libitum*. Randomization was applied prior to all experiments.

Some db/m^+^ and db/db mice were intravenously injected with saline or cilastatin (30 mg/kg; Sigma-Aldrich) twice a week for 4 consecutive weeks. For AAV-mediated Dpep1 knockdown *in vivo*, some db/m^+^ and db/db mice were intravenously injected with AAV1-scramble or AAV1-ICAM2-shDpep1 (2 x 10^11^ vg per mouse), generated by Vigene Biosciences (Maryland, USA). The ICAM2 core promoter sequence was:

CCAAGGGCTGCCTGGAGGGAGATGGTGGGCGCAGGTCTGAGCTATGGCCCAGAATCCCTAGCCTTCTGCAAACTGATGACTGCATTTCCTCTCATTATCTGAGAGATCTTTGGGAAGCCACGTGCACCAGCTCGTTCTAG. Some db/m^+^ and db/db mice were intraperitoneally injected with saline or dexamethasone (5 mg/kg; Sigma-Aldrich) and were sacrificed at different time points: 1 hr, 24 hr, or after 4 weeks of daily injections. Prior to sacrifice, mouse body weights and fasting glucose levels were recorded, and oral glucose tolerance test (OGTT) was performed via tail venous blood collection 6 hr post-fasting.

*Frozen section and* *H&E staining*

Descending thoracic (above diaphragm) and abdominal aortas (below diaphragm) of db/m^+^ and db/db mice (*n* = 4 individual mice per group) were dissected free of adhering connective tissues in ice-cold PBS. The central ~5 mm segments of both descending thoracic and abdominal aortas were harvested to minimize the edge effects from adjacent vascular regions, thereby eliminating interference from branch ostia and peripheral microvascular networks. After excess PBS on the tissue surface was removed, the aortic segments were soaked in liquid nitrogen pre-cooled isopentane for 1 min and was then embedded in optimum cutting temperature compound (OCT; Sakura Finetek, Flemingweg, Holland), followed by snap frozen. The OCT-embedded segments were sectioned at 10 μm (cross section) on a CM1950 cryostat (Leica, Wetzlar, Germany) and mounted onto PEN membrane-coated glass slides (415190-9041-000; Zeiss, Oberkochen, Germany). The aortic sections were stained with H&E as following. The slides were incubated at 37^o^C for 1 min and were subsequently incubated in pre-cooled methanol at -20^o^C for 30 min. After volatilization of residual methanol, the slides were processed with isopropanol (1 min), hematoxylin (7 min), bluing buffer (2 min), and eosin (40 s). The H&E-stained aortic sections were then incubated at 37^o^C for 5 min, and were visualized on digital slide scanner SLIDEVIEW VS200 (Olympus, Tokyo, Japan) at 20x magnification. WTTR was calculated to assess aortic structure. The samples on the dried slides were later subjected to laser microdissection and protein digestion for MS-based proteomics.

*Measurement of vascular tone by wire myography*

After mice were sacrificed via CO_2_ suffocation, descending thoracic and/or abdominal aortas were dissected free of adhering connective tissues in sterile PBS. Arterial segments (length ≈ 2 mm) were prepared in ice-cold oxygenated Krebs solution (in mmol/L: NaCl 119, NaHCO_3_ 25, KCl 4.7, MgCl_2_ 1, KH_2_PO_4_ 1.2, CaCl_2_ 2.5, D-glucose 11). Segments were individually mounted on a Multi Wire Myograph System (Danish Myo Technology, Hinnerup, Denmark) for isometric tension measurement. Baseline tension of aortas was set to 3 mN. Segments equilibrated for 30 min at 37^o^C in oxygenated (95% O_2_, 5% CO_2_) Krebs solution. Following equilibration, segments were pre-contracted with 60 mmol/L KCl, followed by three-time rinsing in Krebs solution. Segments were then pre-contracted with phenylephrine (Phe; 3 μmol/L; Sigma-Aldrich). EDR was assessed by cumulative addition of acetylcholine (ACh; 10 nmol/L - 10 μmol/L; Sigma-Aldrich). Endothelium-independent relaxation was evaluated by cumulative addition of SNP (1 nmol/L - 10 μmol/L; Sigma-Aldrich). Changes in isometric tension were recorded by using a PowerLab LabChart 7.0 system (AD Instruments, Bella Vista, NSW, Australia).

*Measurement of NO production by nitrite assay*

To assess NO production, mouse aortas were first incubated with ACh (10 μmol/L) at 37^o^C for 10 min. The aortas were then treated with nitrate reductase to reduce nitrate to nitrite. After homogenization, supernatants were assayed for nitrite concentration by using a colorimetric Griess reaction kit (Molecular Probes, Eugene, OR, USA). Absorbance readings at 548 nm were referenced against a nitrite standard curve. Nitrite values were normalized to the protein contents of the homogenates, as measured by the Bradford assay.

*Western blotting*

Aortic tissues and cultured ECs were homogenized in ice-cold RIPA lysis buffer (1X), supplemented with Complete Protease Inhibitor Cocktail (Sigma-Aldrich) and PhosSTOP phosphatase inhibitor (Roche, Basel, Switzerland). Protein concentrations were determined using a BCA protein assay kit (Pierce Biotechnology, Rockford, IL, USA). Equal amounts of protein were resolved by 10% SDS-polyacrylamide gel and transferred to Immobilon-P polyvinylidene difluoride membranes (Millipore Corp., Burlington, MA, USA). Membranes were blocked for 1 hr in TBS containing 3% BSA and 0.05% Tween-20. Blots were then incubated overnight at 4^o^C with primary antibodies, followed by incubation with horseradish peroxidase-conjugated secondary antibodies (Cell Signaling Technology, Danvers, MA, USA) for 2 hr at room temperature. All primary antibodies in the current study were listed in Table S6. Protein bands were visualized by using enhanced chemiluminescence (Cell Signaling Technology) and quantified using a ChemiDoc^TM^ Imaging System (Bio-Rad, Hercules, CA, USA).

*Measurement of vascular superoxide anion production*

Generation of superoxide anion (O_2_˙⁻) in freshly isolated mouse aortas was evaluated by lucigenin-enhanced chemiluminescence assay. Aortic tissues were equilibrated for 45 min at 37^o^C in Krebs-HEPES solution (in mmol/L: NaCl 99, KCl 4.7, NaHCO_3_ 25, KH_2_PO_4_ 1, Na-HEPES 20, CaCl_2_ 2.5, MgSO_4_ 1.2, D-glucose 11), containing diethyldithiocarbamic acid (1 mmol/L, Sigma-Aldrich) and β-NADPH (0.1 mmol/L, Sigma-Aldrich). Subsequently, the tissues were transferred into vials containing Krebs-HEPES solution with 10 μmol/L lucigenin (Sigma-Aldrich). Chemiluminescent signals were recorded at 1-min intervals for 10 min using a GloMax^®^ 20/20 Luminometer (Promega, Madison, WI, USA). Data were normalized to tissue dry weight and presented as relative light units per mg.

*Measurement of NOX activity*

NOX enzymatic activity in aortic tissues was quantified spectrophotometrically by using a commercial assay kit (Genmed Scientifics, Shanghai, China), following the manufacturer’s protocol. Briefly, aortic tissues were homogenized in PBS and centrifuged at 2500 xg for 10 min. Supernatants were incubated with NADPH, and enzyme activity was determined by tracking NADPH oxidation kinetics at 340 nm, where NADPH consumption was inhibited by diphenyliodonium. Nox activity values were normalized to tissue weight and differences in NOX activity were expressed as percentage change relative to control samples.

*RT-PCR*

Total RNA from aortic tissues was isolated using TRIzol reagent (Invitrogen). cDNA was synthesized from RNA using the iScript^TM^ cDNA synthesis kit (Bio-Rad). Quantitative RT-PCR amplification was performed on an ABI ViiA7 system (Applied Biosystems) with SYBR Premix ExTaq (TakaRa, Kusatsu, Japan). All primer sequences in the current study were listed in Table S7. Gadph served as the endogenous reference gene.

*Laser microdissection*

LCM was performed on LCM System PALM (Zeiss). The LCM system was initially turned on for 15 min to stabilize laser energy. The microscope and laser settings were set up as following: Zoom: 20x; cut energy: 48; focus: 62; catapulting energy:20; focus: 70; cycle number: 1; cut speed: 5. The dried H&E-stained slide of aortic section was put onto the slide adapter of the LCM microscope. The regions of interest in the section was marked with LCM marker pen, microdissected using the above settings, and collected by microtubes (415190-9201-000, Zeiss). The microdissected samples were stored at -80^o^C for later digestion for MS-based proteomics.

*Protein digestion*

The microdissected samples were resuspended in 5 μL of lysis buffer and subjected to 3-min sonication using a high-intensity contactless ultrasonic processor (Scientz, Zhejiang, China). Subsequently, the lysates were incubated at 95^o^C for 10 min. After cooling to room temperature, dithiothreitol (5 mmol/L) was added for reduction at 56^o^C for 30 min. Subsequently, iodoacetamide (11 mmol/L) was added, followed by incubation at room temperature in the dark for 15 min. For protein digestion, trypsin (10 ng/μL) was added to the samples, followed by overnight digestion at 37^o^C. Following digestion, the resulting peptides were desalted with C18 Zip Tips in accordance with the manufacturer’s protocol and vacuum-dried in preparation for subsequent MS analysis.

*Liquid chromatography-tandem MS and data processing*

The tryptic peptides were dissolved in solvent A and were directly loaded onto a home-made reverse-phase analytical column (15 cm length x 100 μm internal diameter), using a Vanquish Neo UPLC system (Thermo Fisher Scientific). The mobile phase comprised solvent A (0.1% formic acid in water) and solvent B (0.1% formic acid, 80% acetonitrile in water), with chromatographic separation achieved through the following gradient program at a constant flow rate (200 nL/min): 0-1.6 min, 4%-22.5% B; 1.6-2.0 min, 22.5%-35% B; 2.0-2.1 min, 35.0%-35.1% B; 2.1-2.3 min, 35.1% B; 2.3-9.2 min, 35.1%-35.2% B; 9.2-9.6 min, 35.2%-55.0% B; 9.6-10.1 min, 55.0%-99.0% B; 10.1-12.0 min, 99% B. The separated peptides were ionized using a nano-electrospray ion source at 1900 V and were analyzed on an Orbitrap Astral mass spectrometer (Thermo Fisher Scientific). Full-scan MS spectra (400-800 m/z) were acquired in the Orbitrap mass analyzer at a resolution of 240,000, while tandem MS (MS/MS) spectra were obtained in the Astral analyzer with a resolution of 80,000, using a fixed first mass of 150.0 m/z. Higher-energy collisional dissociation was performed at a normalized collision energy (25%). Automatic gain control was set to 800% with a maximum ion injection time (15 ms) to optimize sensitivity and dynamic range.

Data-independent acquisition (DIA) data were analyzed using the DIA-NN computational platform (v1.8). Tandem mass spectra were queried against the Mus_musculus_10090_SP_20231220.fasta (17191 entries), concatenated with reverse decoy database. Trypsin/P was designated as the proteolytic enzyme with allowance for up to one missed cleavage. Fixed modifications included N-terminal methionine excision and cysteine carbamidomethylation. Peptide and protein identifications were filtered at a global false discovery rate threshold of 1%. Further data filtering on the database search results was performed to enhance analytic quality, by ensuring that the identified proteins must contain at least one peptide.

*Identification of DEPs*

DEPs between experimental groups (*n* = 4 per group) were identified via statistical analysis of normalized protein intensity values using the R package “limma”, which calculates *p* values via two-sample Student’s t-test and Log_2_ fold changes (FCs). Proteins with both significance (*p* value < 0.05) and biological relevance thresholds (|Log_2_(FC)| ≥ Log_2_(1.5), equivalent to FC ≥ 1.5 or ≤ 1/1.5) were classified as DEPs. To analyze sample clustering based on similarities in protein expression profiles, we performed PCA unsupervised hierarchical clustering of sample-to-sample distances. PCA plot was generated with the R package “ggplot2”, while the hierarchically clustered heatmap was created with the R package “pheatmap”. To highlight significant DEPs with indicated thresholds, volcano plot displaying -log_10_(*p* value) against Log_2_(FC) was generated with the R package “ggplot2”. Dynamic radar plot illustrating the *p* values, Log_2_(FC), and mean quantitative levels per group was generated with the R package “ggiraphExtra”. Subcellular localization of DEPs was predicted using WoLF PSORT, which infers localization based on amino acid sequence features such as sorting signals, amino acid composition, and functional motifs, was used. For visualization of DEP distribution across compartments, a Nightingale rose diagram was generated with the R package “ggplot2” with polar coordinate transformation (coord_polar()).

*Expression pattern clustering*

To evaluate the expression pattern across aortic segments, we performed expression pattern clustering. Relative protein expression values were subjected to Log_2_ transformation to stabilize variance. Proteins with high expression dynamics (SD > 0.3) were retained, resulting in 596 dynamically regulated proteins for clustering analysis using the “Mfuzz” package in R (with the fuzzifier parameter set to 2). These proteins were grouped into six distinct clusters, which provided optimal separation of temporal expression trajectories. To characterize biological properties of clustered proteins, enrichment analyses were conducted for KEGG, GO, and protein domains using R package “clusterProfiler”.

*Functional and pathway enrichment analysis of DEPs*

GO enrichment analysis was performed using eggnog-mapper (v2.1.6) to assign GO IDs to identified proteins via the EggNOG database (<http://eggnog5.embl.de/#/app/home>), followed by functional classification across cellular components (CC), molecular functions (MF), and biological processes (BP). KEGG pathway annotation was performed against the KEGG database (<http://www.kegg.jp/kegg/mapper.html>). Results were visualized through bubble plots (showing protein numbers/*p* values), bar plots (ranking enriched terms), and chord diagrams (gene-pathway networks), generated with the R packages “ggplot2”, “enrichplot”, and “GOplot”, respectively.

*Human thoracic aortic snRNA-seq analysis*

snRNA-seq data from human thoracic aortic samples were extracted from a dataset publicly available at NCBI’s Gene Expression Omnibus database (accession number: GSE165824) and the Broad Institute’s Single Cell Portal (<https://singlecell.broadinstitute.org/single_cell>). Uniform manifold approximation and projection plot, and violin plots were generated using tools from the same portal. Dot plots were generated with the R packages “Seurat” and “ggplot2”.

*Annotation of functional DEPs*

TRs among the DEPs were identified using established TR-target gene regulatory databases, including TRRUST (<https://www.grnpedia.org/trrust/>) and GTRD (<https://gtrd.biouml.org/>), with analysis restricted to mouse. Shear-responsive effectors in DEPs were annotated based on the list of known effectors summarized by a previous literature.

*PPI network*

PPI networks of DEPs were constructed via the STRING database (<https://string-db.org>), with interactions filtered at a high-confidence cutoff (score ≥ 0.7). PPI networks were visualized by the R package “visNetwork”.

*Drug-target interaction prediction*

Drug-target interactions were queried from DrugBank (v5.1.12), a curated knowledgebase integrating potential drug mechanisms, drug target information, FDA approval status, and pharmacokinetic properties. Annotated TRs were converted to UniProt protein identifiers to standardize database queries. Interactions were filtered to retain FDA-approved drugs, and drugs with defined activator/inhibitor mechanisms. Filtered interactions were visualized as a Sankey diagram using the R package “ggalluvial”.

*Human arteries*

Human renal arteries (*n* = 8) were collected from non-diabetic and diabetic patients who underwent nephrectomy. Demographic characteristics of the patients were presented in Table S8. The study was conducted in accordance with the Declaration of Helsinki and was approved by the Clinical Research Ethics Committee of The Chinese University of Hong Kong (CUHK; Approval No.: 2014.468 and 2018.055). All arterial samples were procured by the Department of Surgery at Prince of Wales Hospital, CUHK. All participants signed an informed consent prior to their inclusion in the study.

*Ex vivo tissue culture*

After C57BL/6 mice were sacrificed by CO_2_ suffocation, the whole aortas were dissected free from connective tissues in sterile PBS and were then cultured in low glucose DMEM (Life Technologies, Paisley, UK), supplemented with 10% FBS and antibiotics. For pharmacological studies, some aortas were incubated with HG (25 mmol/L), mannitol (19.5 mmol/L), or LPS (1 μg/mL; Sigma-Aldrich) for 24 hr.

*Serum LPS quantification*

Prior to analysis, mouse serum samples were diluted 1:50-1:100 under pyrogen-free conditions and heat-inactivated at 70 °C for 15 min. Serum LPS levels were then measured using a LAL Chromogenic Endotoxin Quantitation kit (Pierce, Massachusetts, USA), following the manufacturer’s protocol.

*Cell culture*

HAECs (CC-2535; Lonza, MD, USA) were cultured in EGM-2MV bullet kit (Lonza) supplemented with 10% FBS and antibiotics at 37^o^C until confluence. Experiments used cells at passages 4-7. For pharmacological studies, HAECs were incubated with HG (25 mmol/L) or LPS (1 μg/mL) for 24 hr. Some HAECs were incubated with dexamethasone (100 nmol/L) for 1hr or 24 hr.

*Measurement of Dpep1 enzymatic activity*

The enzymatic activity of endogenous Dpep1 *in vivo* was measured as previously described. In brief, mouse aortic tissues were harvested, weighed, and washed with PBS. Proteins were isolated from tissues using octyl-glucopyranoside/RIPA in the absence of protease inhibitors, followed by incubation with the membrane dipeptidase substrate Gly-D-Phe (Sigma-Aldrich) at 37^o^C for 1 hr and 40 min. Following incubation, 50 μL/well of reaction assay buffer was added, and the plate was incubated at 37^o^C for 40 min in the dark. Fluorescence signals generated by the enzymatic conversion of D-Phe to 6, 69-dihydroxy-(1, 19-biphenyl)-3, 39-diacetic acid, in the presence of peroxidase (Sigma-Aldrich) and D-amino acid oxidase (Sigma-Aldrich), were measured using a fluorescence plate reader (excitation: 317 nm; emission: 414 nm).

*MPO activity assay*

To quantify MPO activity *in vivo*, mouse aortic tissues were harvested, weighed, and snap-frozen in liquid nitrogen. MPO activity was measured according to established methodology. Samples were analyzed in 96-well microtiter plates with absorbance detected at 530 nm using SoftMax PRO software. Data are expressed as absorbance units/mg tissue.

*Aortic endothelial cell isolation*

To isolate mouse aortic ECs, mouse aortic tissues were cut into tiny pieces before placing in 1x dissociation enzyme cocktail (450 U/mL collagenase type I, 60 U/mL DNase I, and 60 U/mL hyaluronidase) at 37 °C for 1 hr. The digestion was stopped by adding FACS buffer (2 mM EDTA, 2% FBS). Cell suspensions were filtered through a 50-μm cell strainer, then incubated with anti-mouse CD31 microbeads on ice for 15 min. Magnetic separation was performed using an LS column (Miltenyi Biotec, Bergisch Gladbach, Germany) placed in a magnetic separator. After washing the column with FACS buffer (3x), CD31^+^ cells were eluted into a 15-mL tube by pushing syringe plug. Cell suspension was subsequently centrifuged, and the cell pellet was ready for protein isolation.

*In vitro hemodynamic simulation*

High LSS (15 dyn/cm^2^) and low LSS (8 dyn/cm^2^) were applied using an ibidi flow system (Gräfelfing, Germany), coupled with custom-built flow chambers (Figure 8C). HAECs (5 × 10^5^ cells) were seeded onto fibronectin (50 μg/mL, 24 hr)-coated glass slides (75 mm × 38 mm; Corning, USA). Following 16-hr attachment, cells were incubated in EGM containing 2% FBS. Slides were then mounted into flow chambers and connected to the ibidi system, followed by LSS exposure for 1hr or 24 hr, in the presence or absence of LPS (1 μg/mL).

*siRNA-mediated genetic knockdown*

72 hr before LSS exposure or dexamethasone treatment, HAECs (~80% confluency) were transfected by either scrambled siRNA (siScr; Cell Signaling Technology) or GR-targeting siRNA (siGR; Cell Signaling Technology) via electroporation by the Amaxa Basic Nucleofector Kit specialized for mammalian ECs (Lonza). Briefly, HAECs were trypsinized, washed twice with PBS, and resuspended in 100 µL of basic Nucleofector solution. The cell suspension was added to an electroporation vial pre-loaded with 50 pmol siScr or siGR. After electroporation with the Amaxa Nucleofector^TM^ apparatus, the cells were cultured in FBS-free DMEM and allowed to recover for 48 hr before seeding onto glass slides for 24 hr-attachment and subsequent LSS exposure.

*Cytoplasmic and nuclear protein fractionation*

Cytoplasmic and nuclear proteins from HAECs and mouse aortas were extracted using the NE-PER Nuclear and Cytoplasmic Extraction Kit (#78833, Thermo Fisher Scientific). Briefly, HAECs were harvested by trypsin-EDTA, pelleted by centrifugation, and washed with ice-cold PBS, while freshly collected aortic tissues were cut into small pieces, washed with ice-cold PBS, and homogenized using a Dounce homogenizer in CER I reagent. Samples were sequentially subjected to lysis using protease inhibitor-containing CER I, CER II, and NER reagents. The cytoplasmic and nuclear extracts were used for Western blotting to determine GR nuclear translocation. β-tubulin and histone H3 served as markers for the cytoplasmic and nuclear fractions, respectively.

*Statistical analysis*

Results are expressed as mean ± SD. Statistical analyses were conducted with GraphPad Prism (Version 10.0). Significance between two groups was determined using two-tailed unpaired *t*-tests and nonparametric Mann-Whitney tests. Comparisons among multiple groups employed Brown-Forsythe and Welch ANOVA tests (unpaired t with Welch’s correction). A *p* value < 0.05 was considered statistically significant.

**PART II: Supplementary Figures**

**
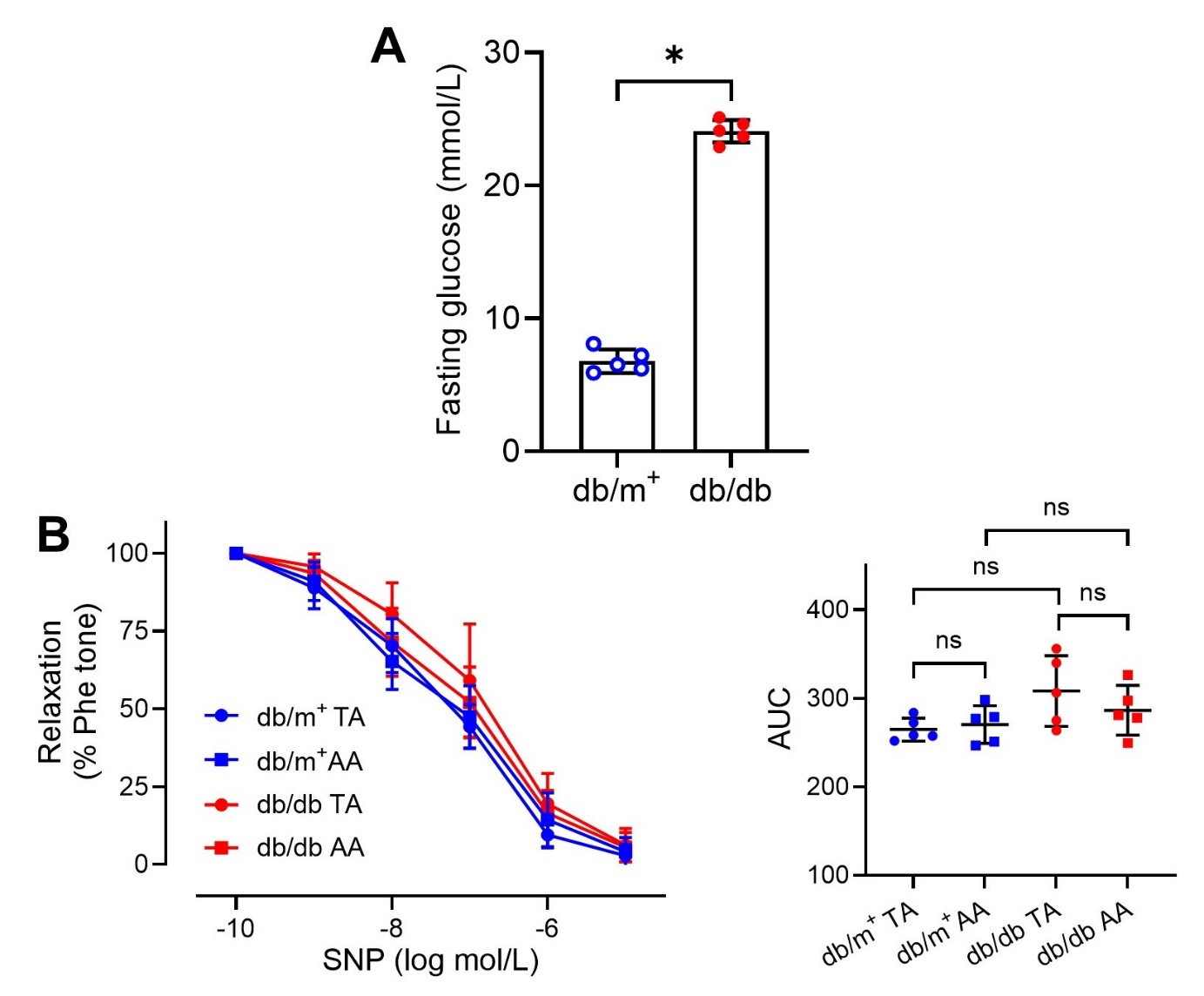
**

**Figure S1.** Diabetic phenotype confirmation and endothelium-independent aortic relaxation. (**A**) Fasting glucose levels of diabetic mice. (**B**) endothelium-independent relaxations of aortas from diabetic mouse aortas and corresponding AUC analysis. *n* = 5 per group. Data are presented as mean ± SD. Brown-Forsythe and Welch ANOVA, and unpaired t with Welch’s correction. AA, abdominal aorta; AUC, area under the curve; Phe, phenylephrine; SNP, sodium nitroprusside; TA, descending thoracic aorta.


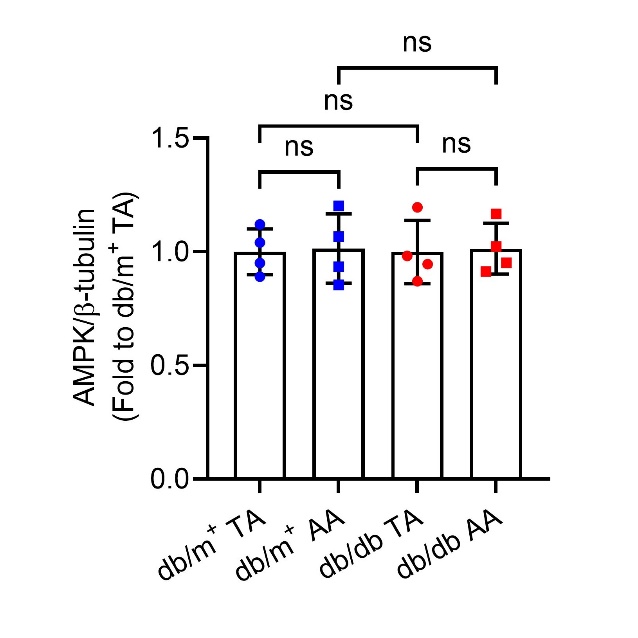


**Figure S2.** Quantification on Western blotting results of AMPK expression. *n* = 4 per group. Data are presented as mean ± SD. Brown-Forsythe and Welch ANOVA, and unpaired t with Welch’s correction. AA, abdominal aorta; AMPK, AMP-activated protein kinase; TA, descending thoracic aorta.


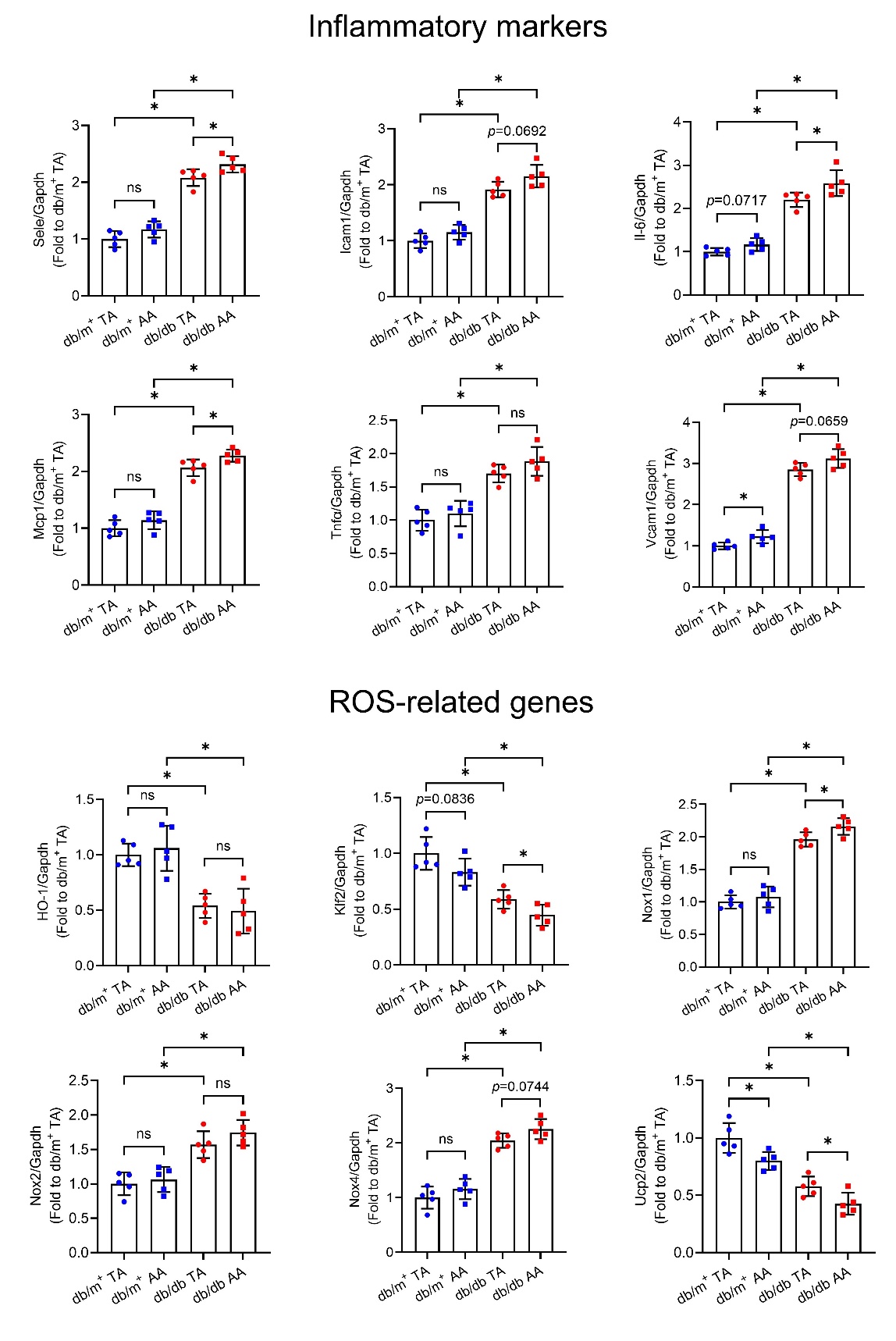


**Figure S3.** RT-PCR on inflammatory markers and ROS-related genes of mouse thoracic and abdominal aortas. *n* = 5 per group. Data are presented as mean ± SD. **p* < 0.05 (Brown-Forsythe and Welch ANOVA, and unpaired t with Welch’s correction). AA, abdominal aorta; TA, descending thoracic aorta.


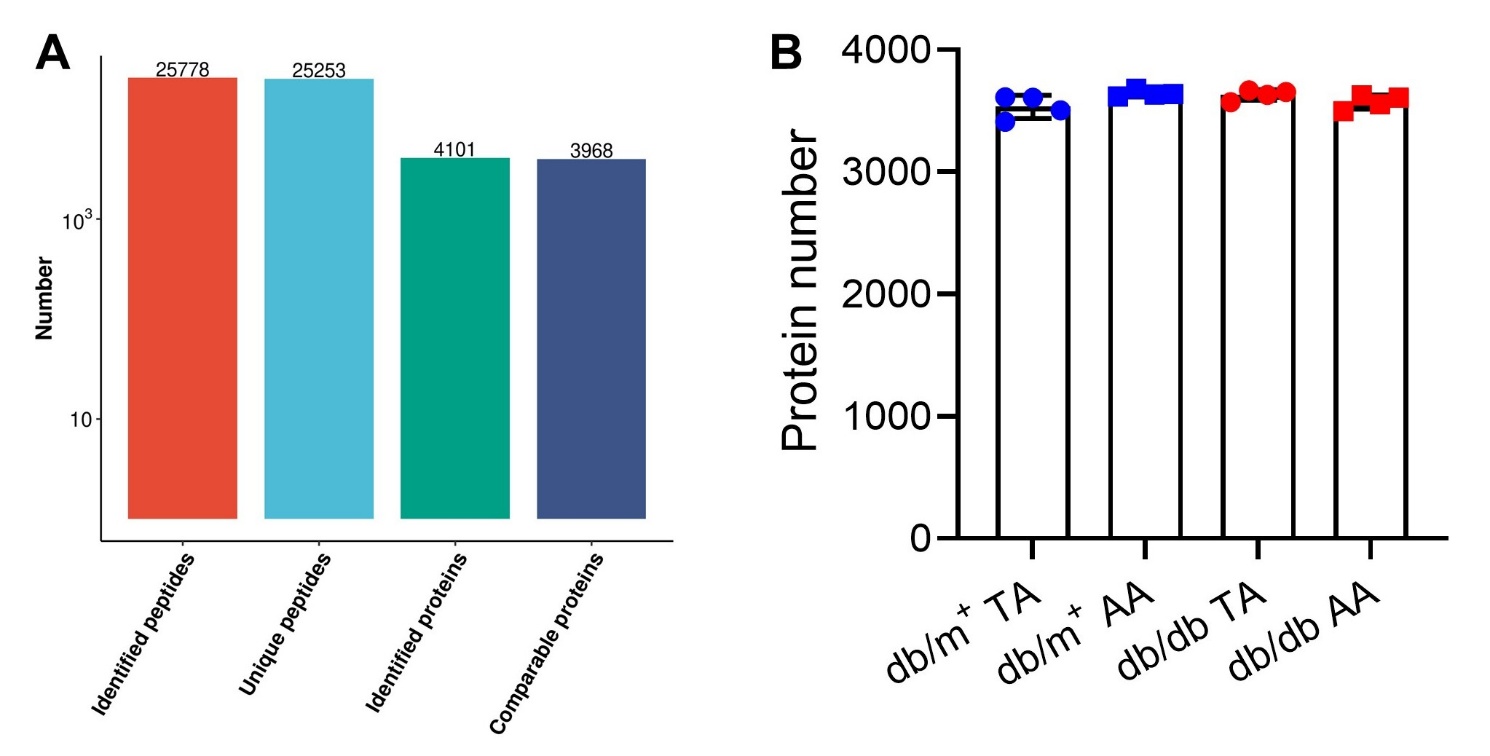


**Figure S4.** Proteome coverage of mouse aortic samples. (**A**) Number of peptides and proteins detected by LCM-based MS. (**B**) Number of proteins detected in different groups of mouse aortic segments. AA, abdominal aorta; LCM, laser-capture microdissection; MS, mass spectrometry; TA, thoracic aorta.


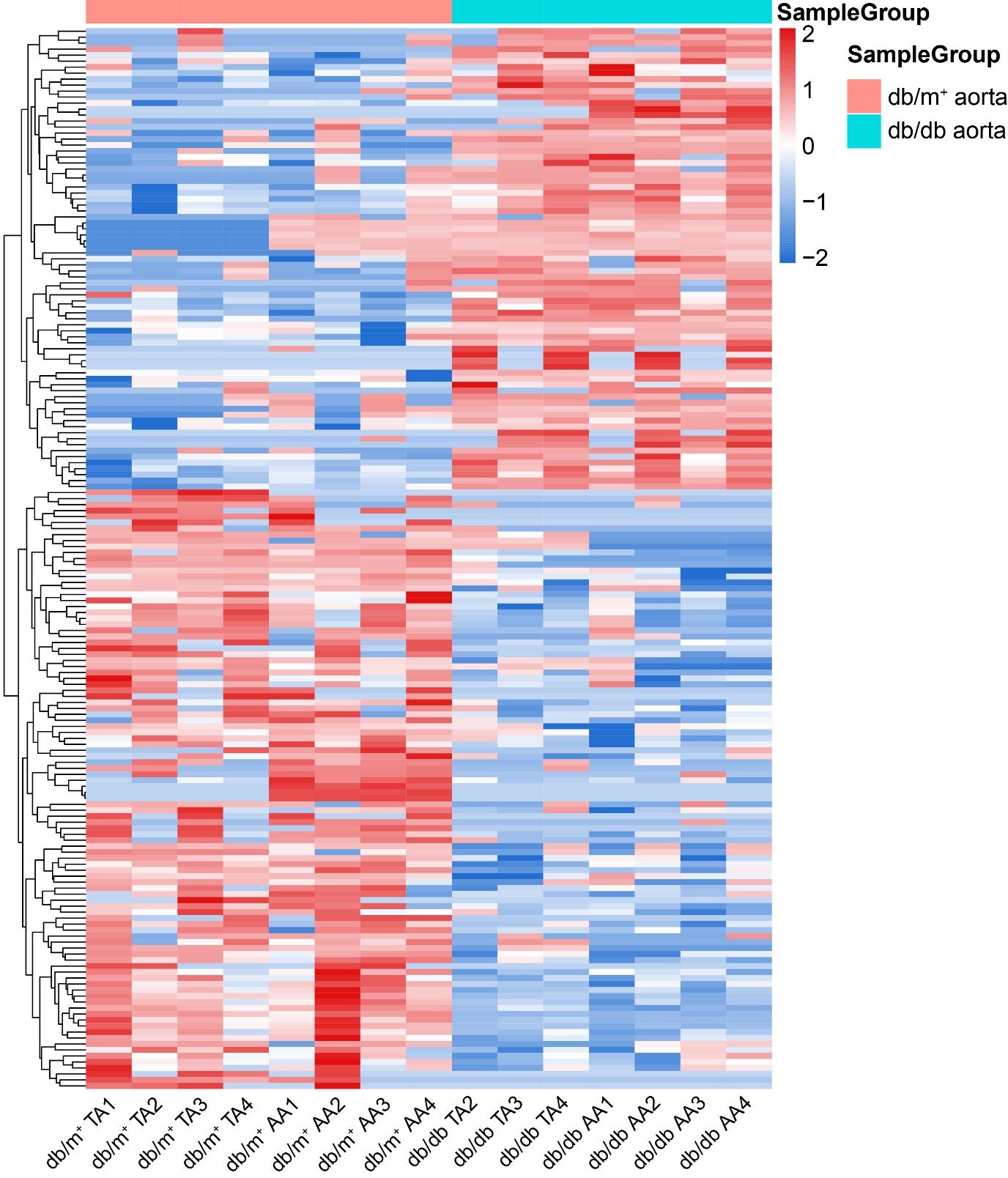


**Figure S5.** Heatmap showing distinct clustering between the two combined groups. *n* = 8 for db/m^+^ aorta; *n* = 7 for db/db aorta. AA, abdominal aorta; TA, descending thoracic aorta.


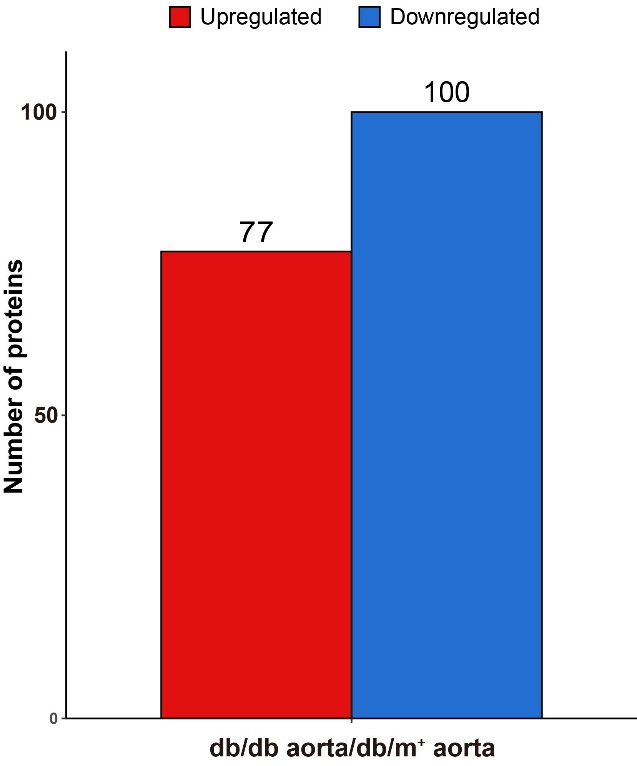


**Figure S6.** Number of upregulated and downregulated DEPs in db/db aorta vs db/m^+^ aorta. DEP, differentially expressed protein.


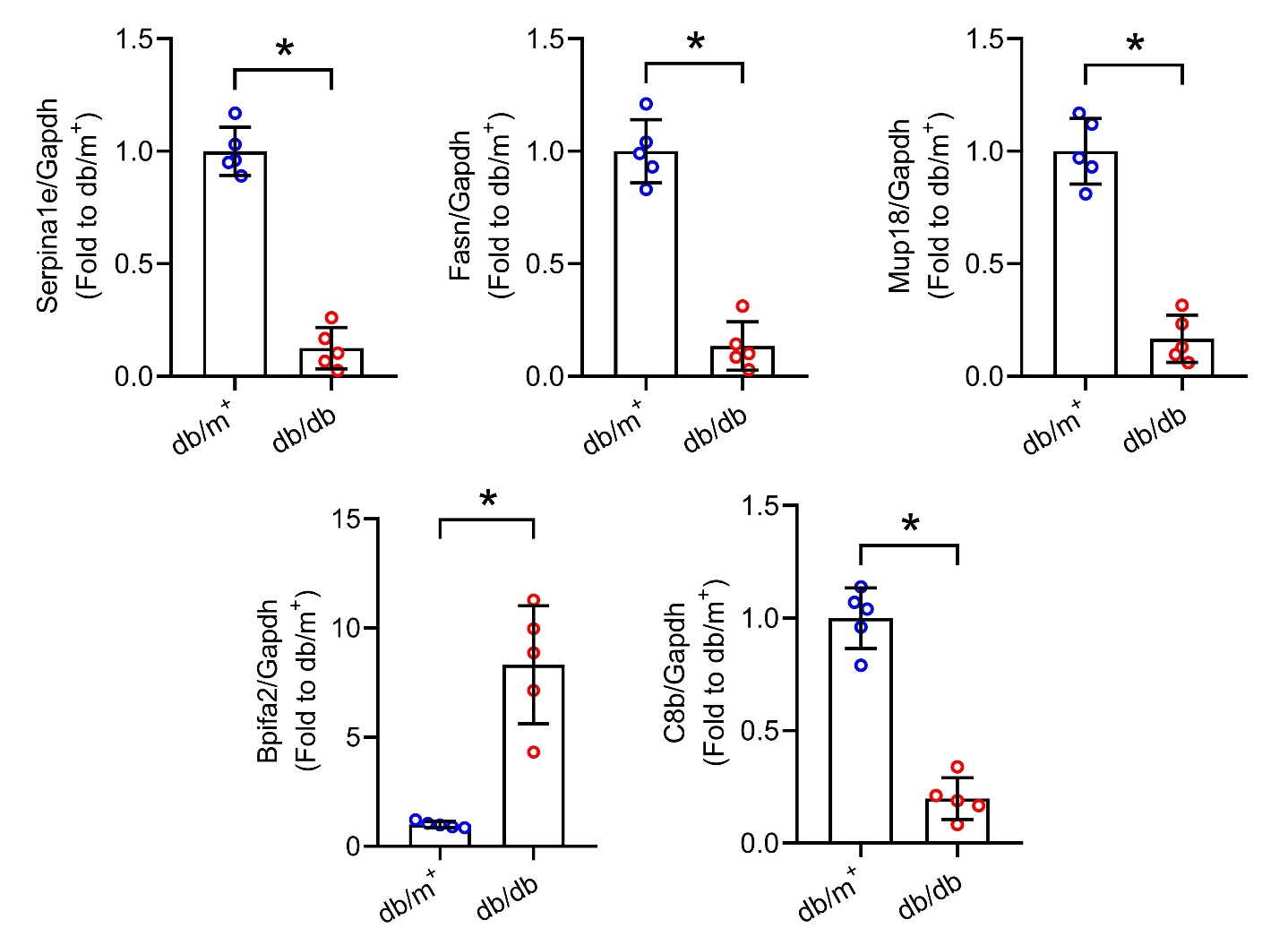


**Figure S7.** RT-PCR on candidate markers in db/m^+^ aorta and db/db aorta. *n* = 5 per group. Data are presented as mean ± SD. **p* < 0.05 (unpaired *t*-tests and nonparametric Mann-Whitney tests).


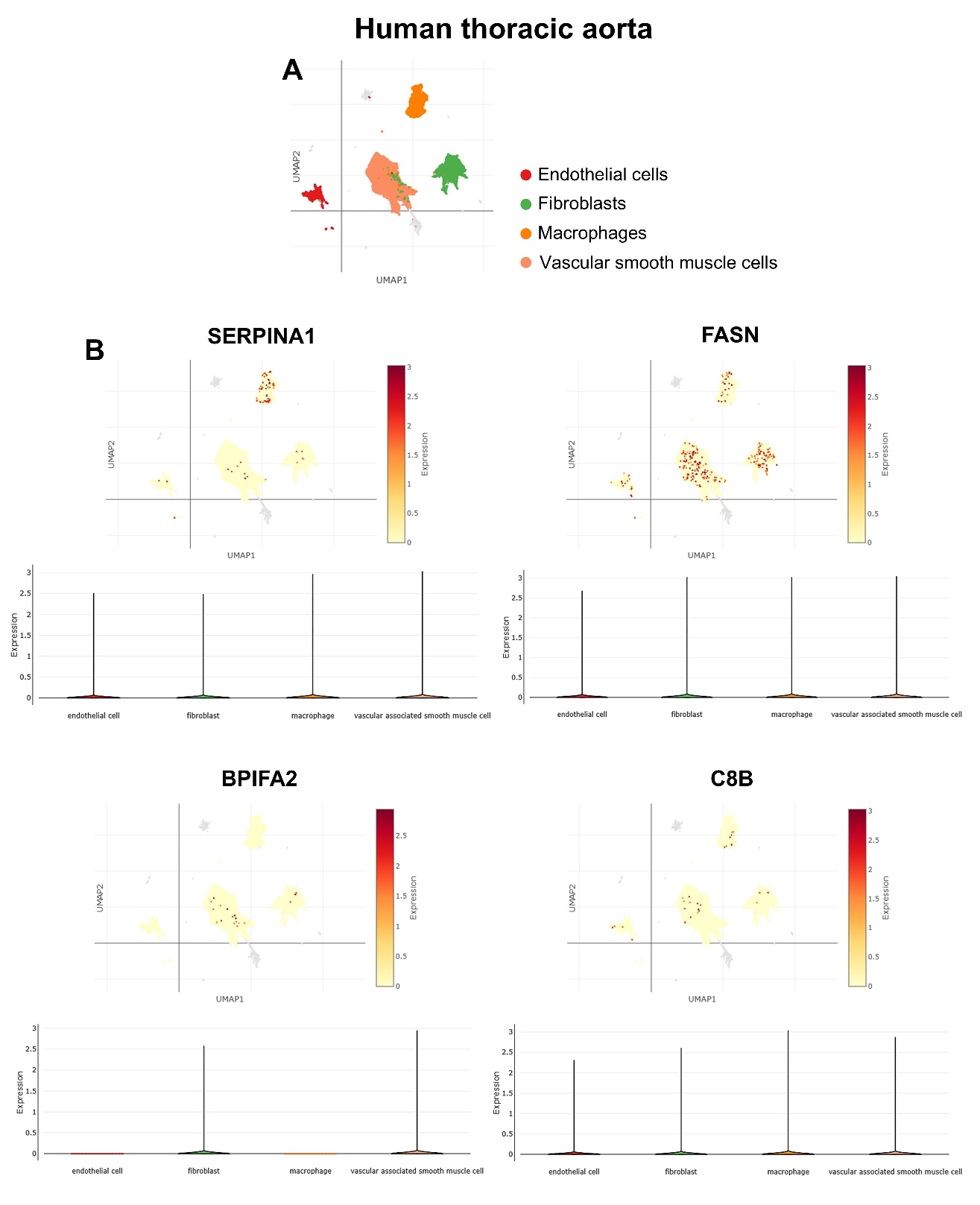


**Figure S8.** Cellular distribution and expression of selected marker genes from mouse aorta in human thoracic aorta based on snRNA-seq data. (**A**) UMAP visualization of 50133 individual nuclei from thoracic aortas of 3 individuals. (**B**) UMAP plots showing the distribution and violin plots showing the expression of selected marker genes in different vascular cell types. snRNA-seq, single-nucleus RNA sequencing; UMAP, uniform manifold approximation and projection.

**
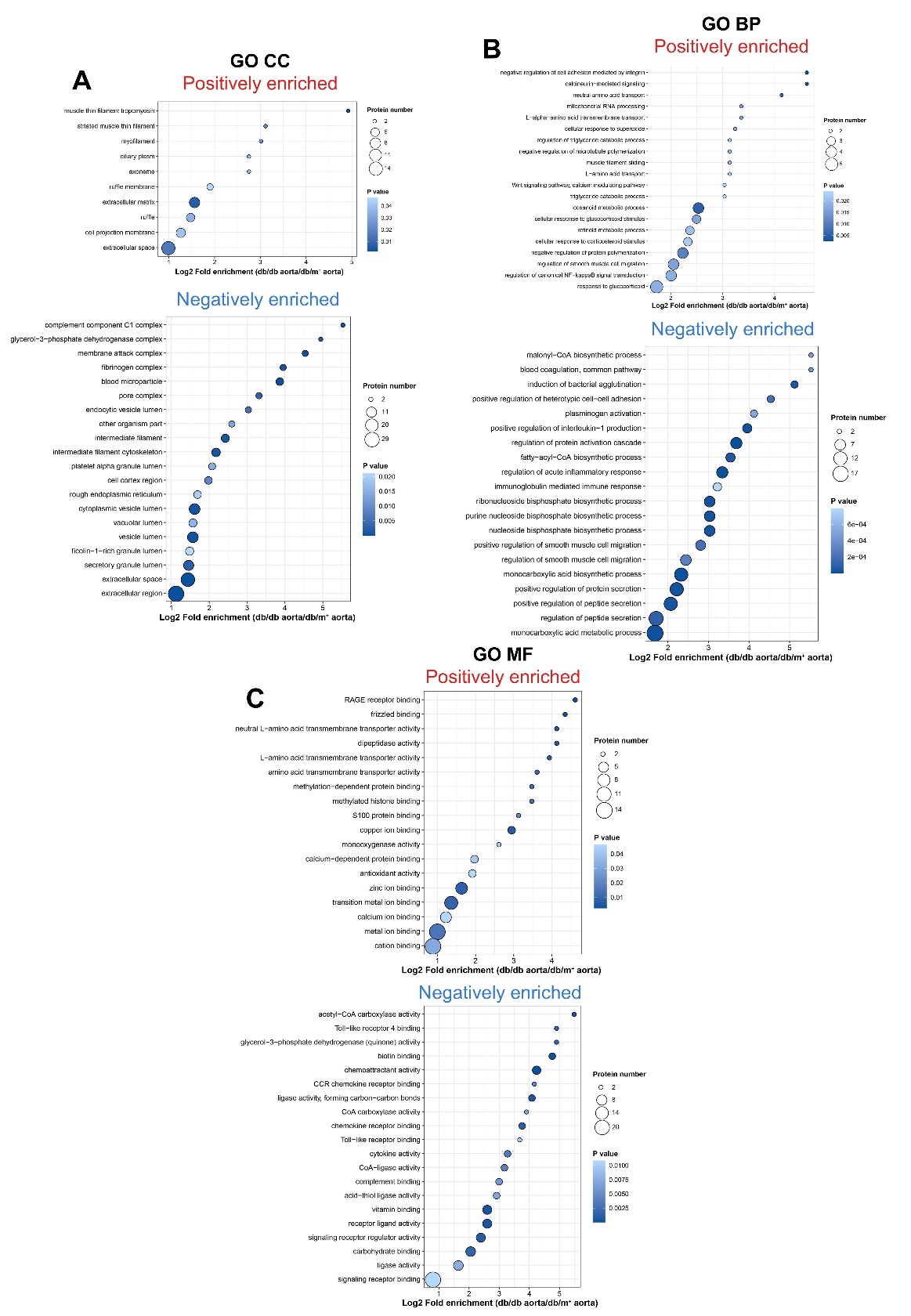
**

**Figure S9.** Positively and negatively enriched GO terms in db/db aorta vs db/m^+^ aorta, ranked by log2 fold enrichment. Dot plots display significantly enriched GO terms (FDR < 0.05) across three ontologies, including (**A**) CC, (**B**) BP, and (**C**) MF. BP, biological process; CC, cellular component; FDR, false discovery rate; GO, Gene Ontology; MF, molecular function.


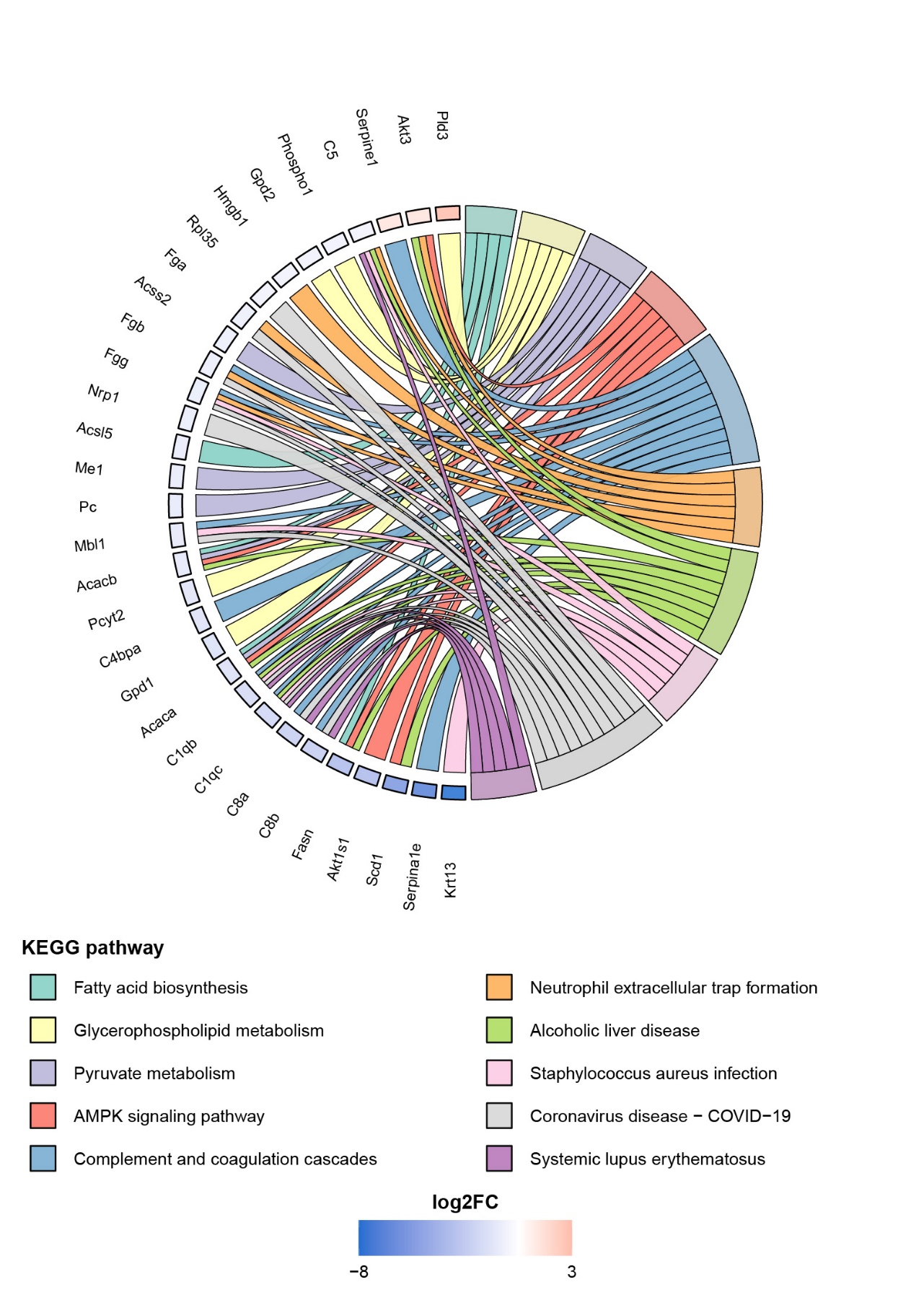


**Figure S10.** Chord diagram linking significantly enriched KEGG pathways to DEPs, ranked by Log_2_FC, in db/db aorta vs db/m^+^ aorta. DEP, differentially expressed protein; FC, fold change; KEGG, Kyoto Encyclopedia of Genes and Genomes.

**
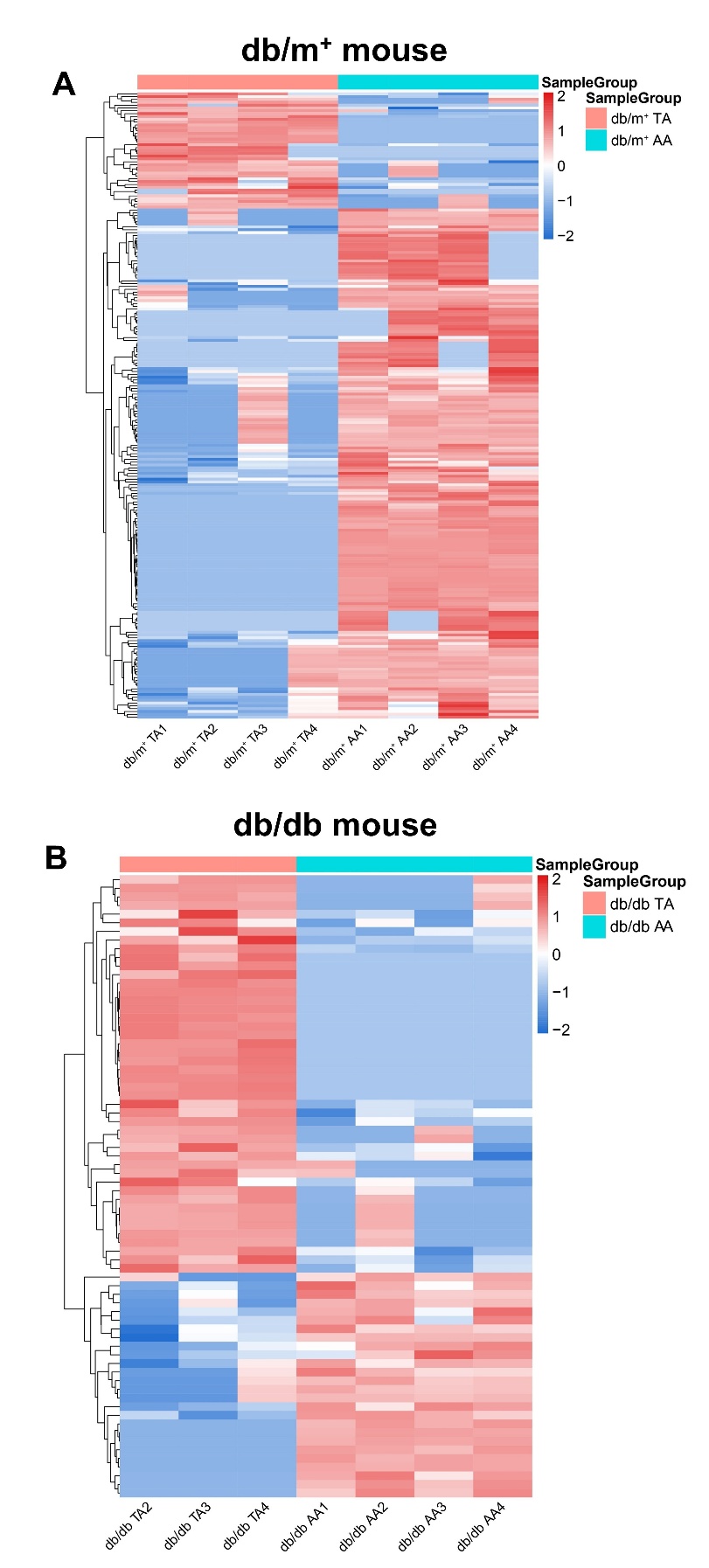
**

**Figure S11.** Heatmaps showing distinct clustering between TA and AA of (**A**) db/m^+^ and (**B**) db/db mice. *n* = 4 for db/m^+^ TA; *n* = 4 for db/m^+^ AA; *n* = 3 for db/db TA; *n* = 4 for db/db AA. AA, abdominal aorta; TA, descending thoracic aorta.

**
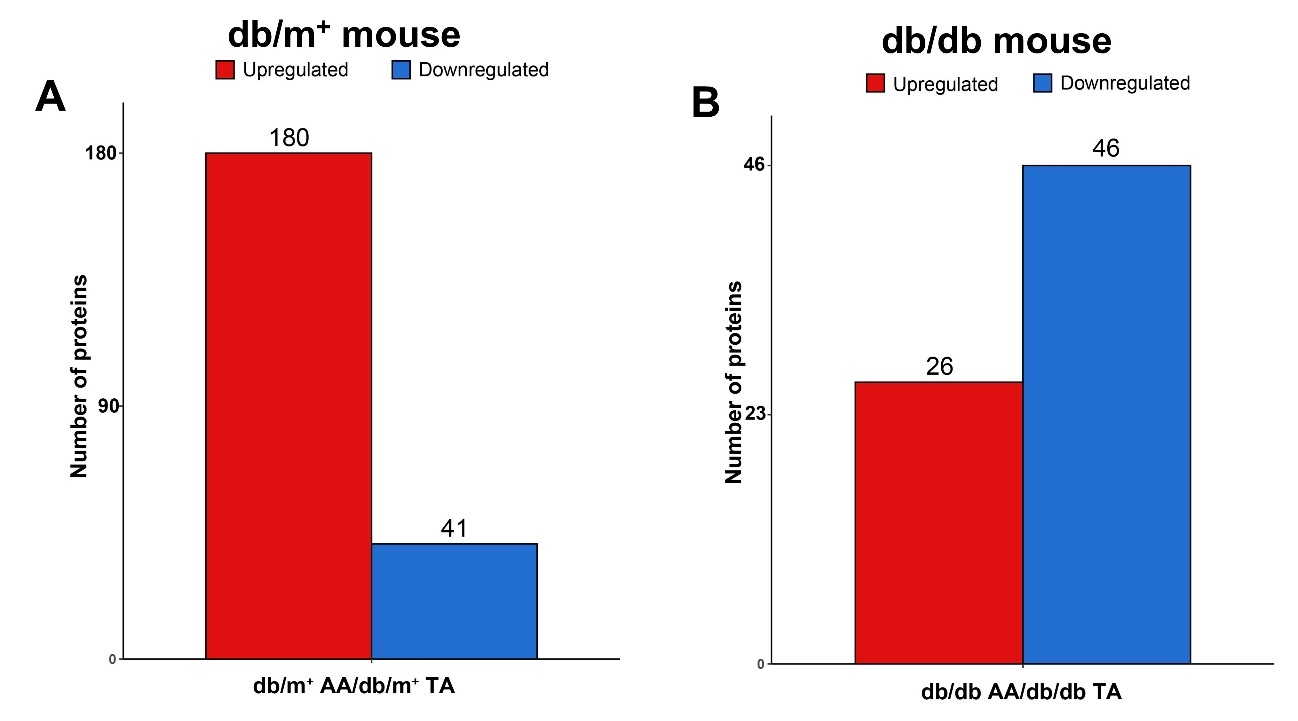
**

**Figure S12.** Number of upregulated and downregulated DEPs in TA and AA of (**A**) db/m^+^ mice and (**B**) db/db mice. AA, abdominal aorta; DEP, differentially expressed protein; TA, descending thoracic aorta.

**
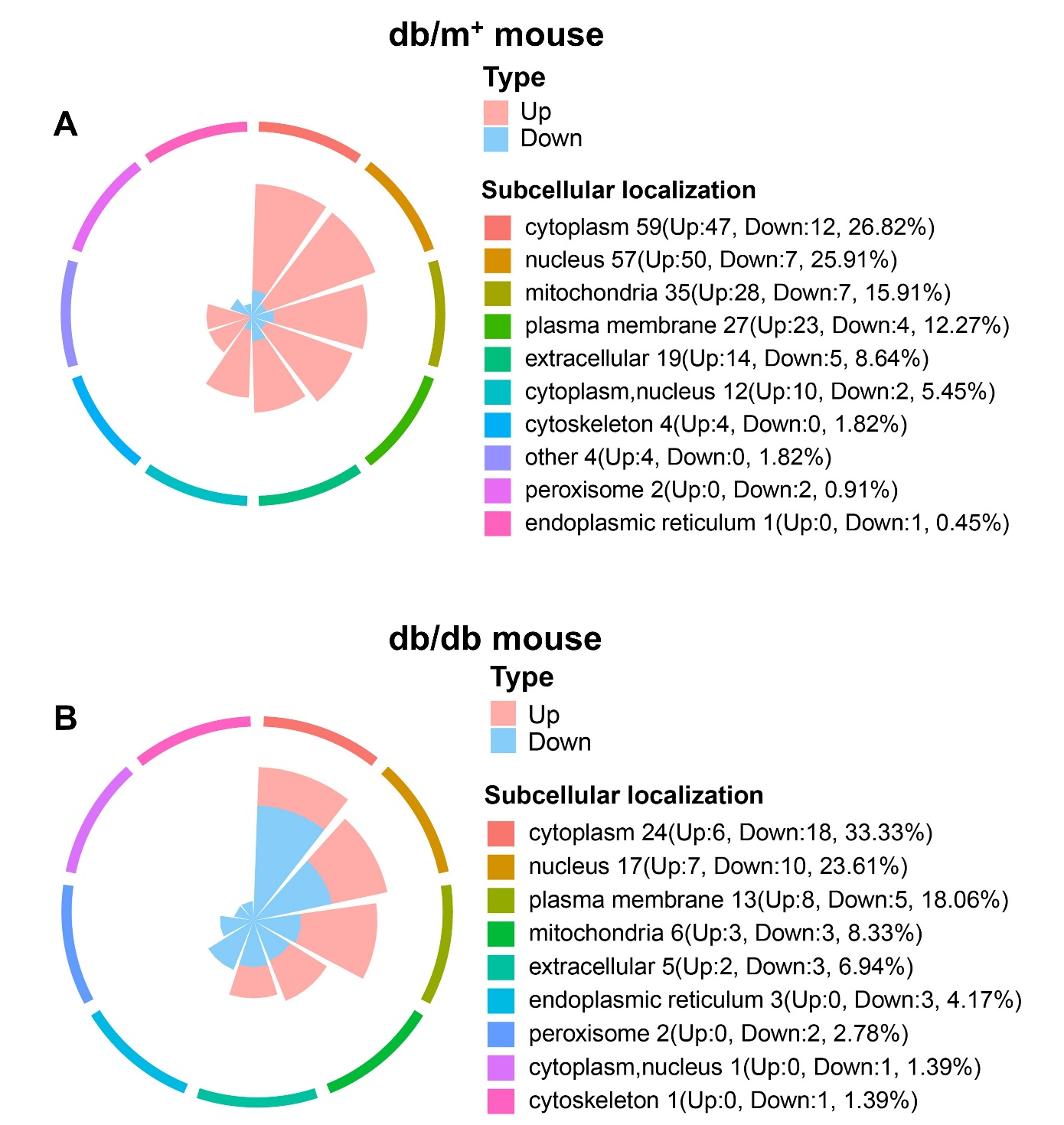
**

**Figure S13.** Nightingale rose diagram on subcellular localizations of upregulated and downregulated DEPs in TA and AA of (**A**) db/m^+^ aorta and (**B**) db/db aorta. AA, abdominal aorta; DEP, differentially expressed protein; TA, descending thoracic aorta.

**
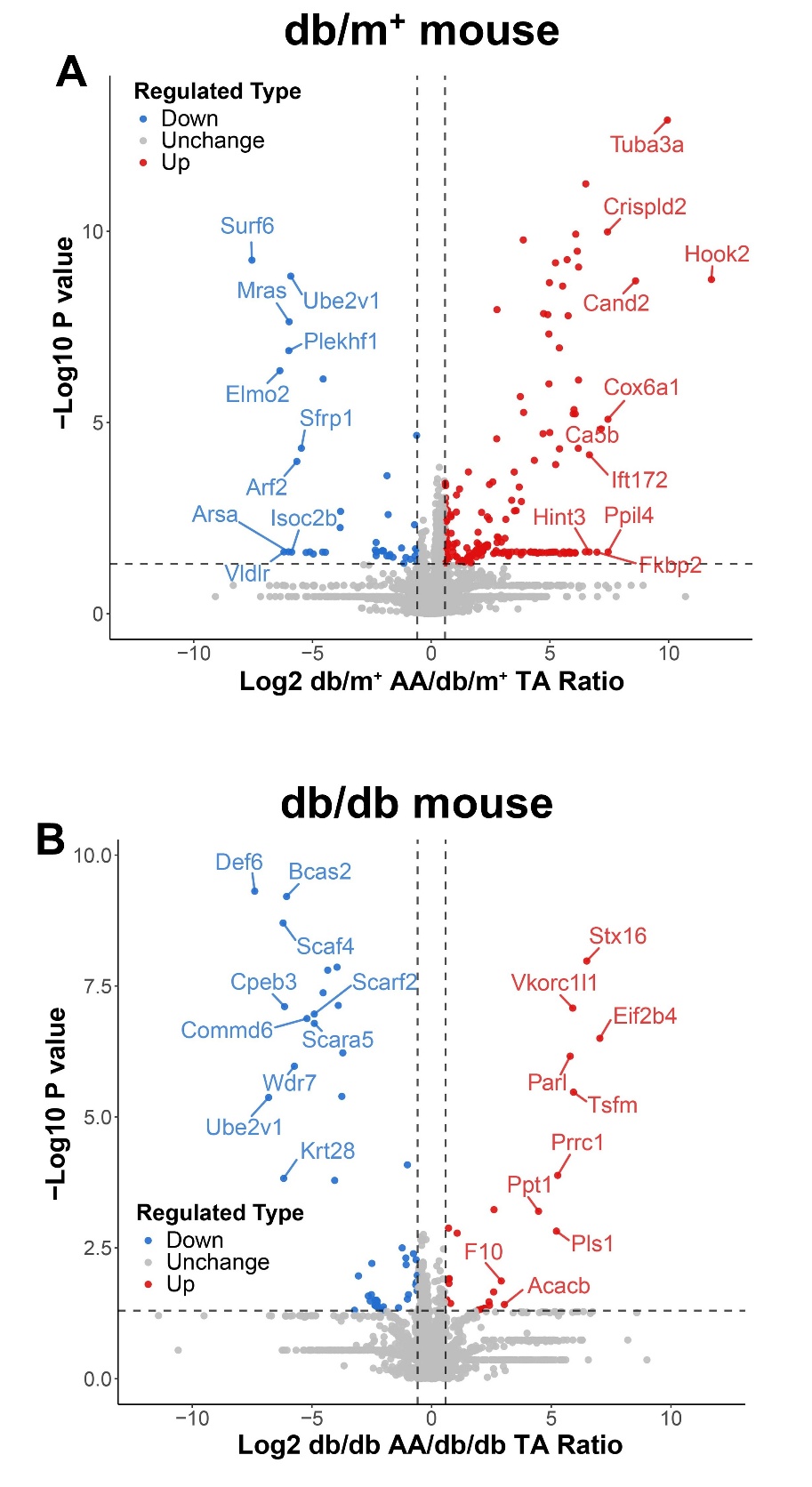
**

**Figure S14.** Volcano plots on DEPs between TA and AA of (**A**) db/m^+^ mice and (**B**) db/db mice. Grey dotted lines: vertical at |Log_2_ FC| = 0.6 (~1.5-fold change) and horizontal at -log_10_ *p* = 1.3 (*p* < 0.05). AA, abdominal aorta; DEP, differentially expressed protein; TA, descending thoracic aorta.

**
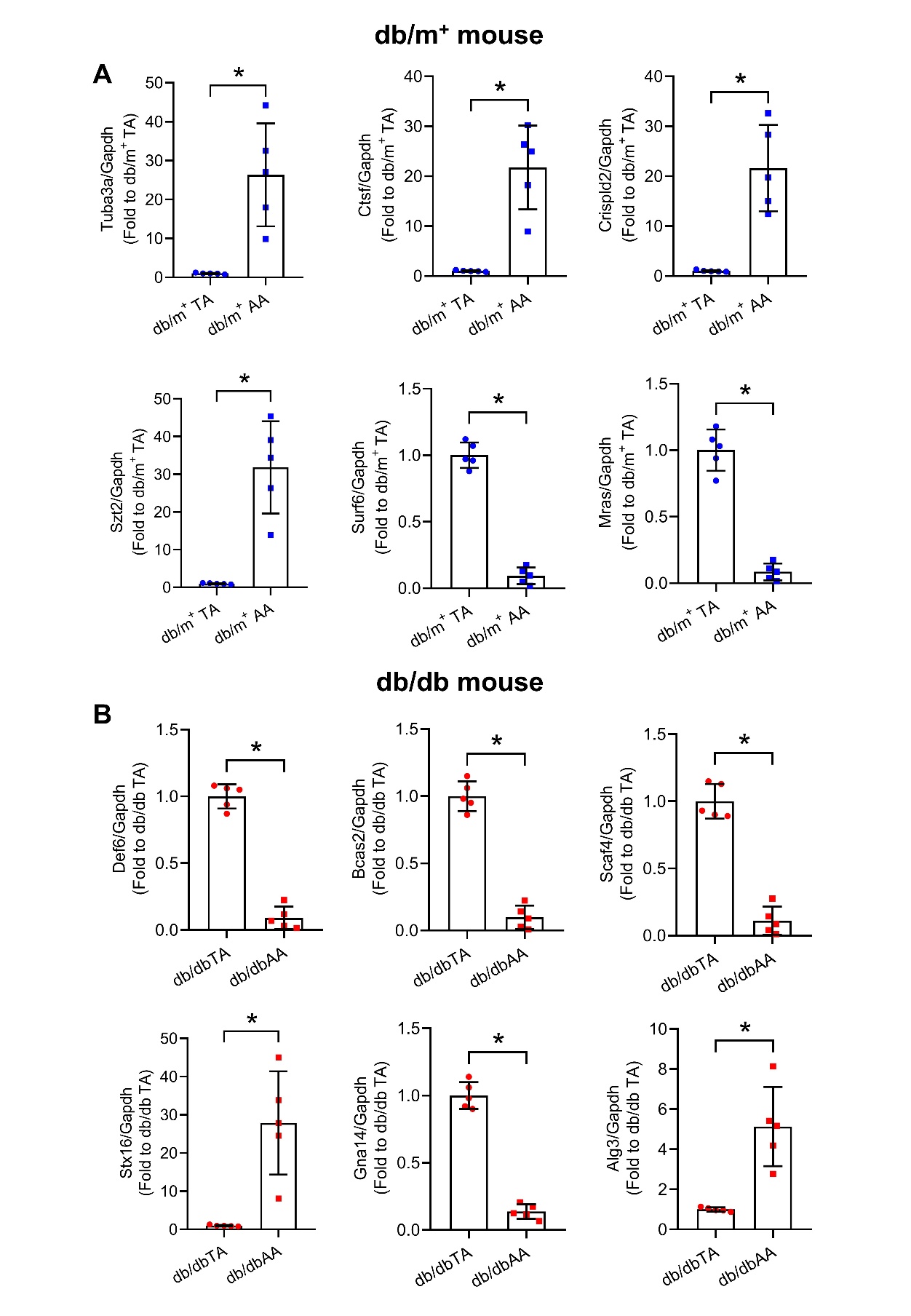
**

**Figure S15.** RT-PCR on candidate markers in TA and AA of (**A**) db/m^+^ mice and (**B**) db/db mice. *n* = 5 per group. Data are presented as mean ± SD. **p* < 0.05 (unpaired *t*-tests and nonparametric Mann-Whitney tests). AA, abdominal aorta; TA, descending thoracic aorta.


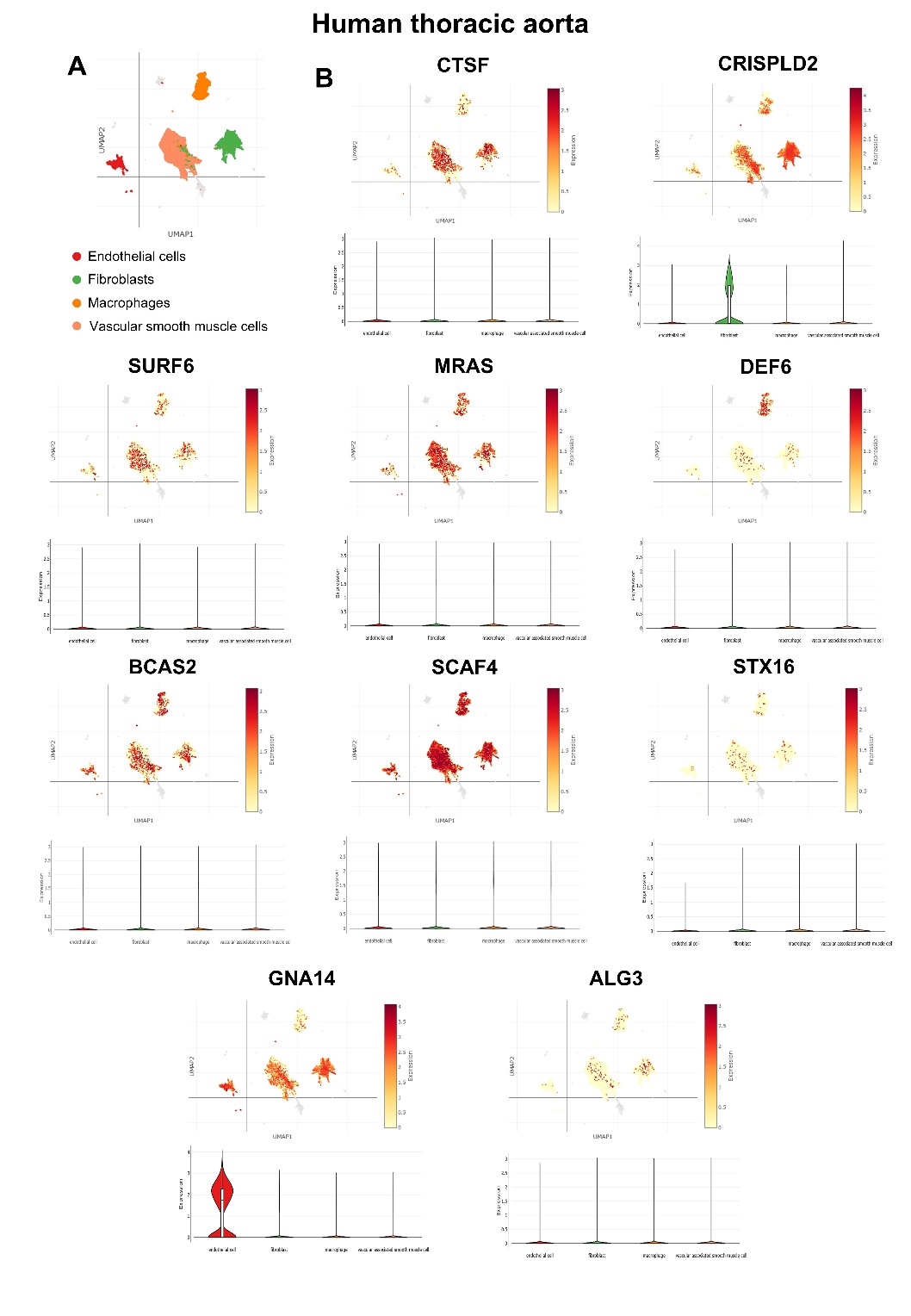


**Figure S16.** Cellular distribution and expression of selected marker genes from mouse TA and AA in human thoracic aorta based on snRNA-seq data. (**A**) UMAP visualization of 50133 individual nuclei from thoracic aortas of 3 individuals. (**B**) UMAP plots showing the distribution and violin plots showing the expression of selected marker genes in different vascular cell types. AA, abdominal aorta; snRNA-seq, single-nucleus RNA sequencing; TA, descending thoracic aorta; UMAP, uniform manifold approximation and projection.


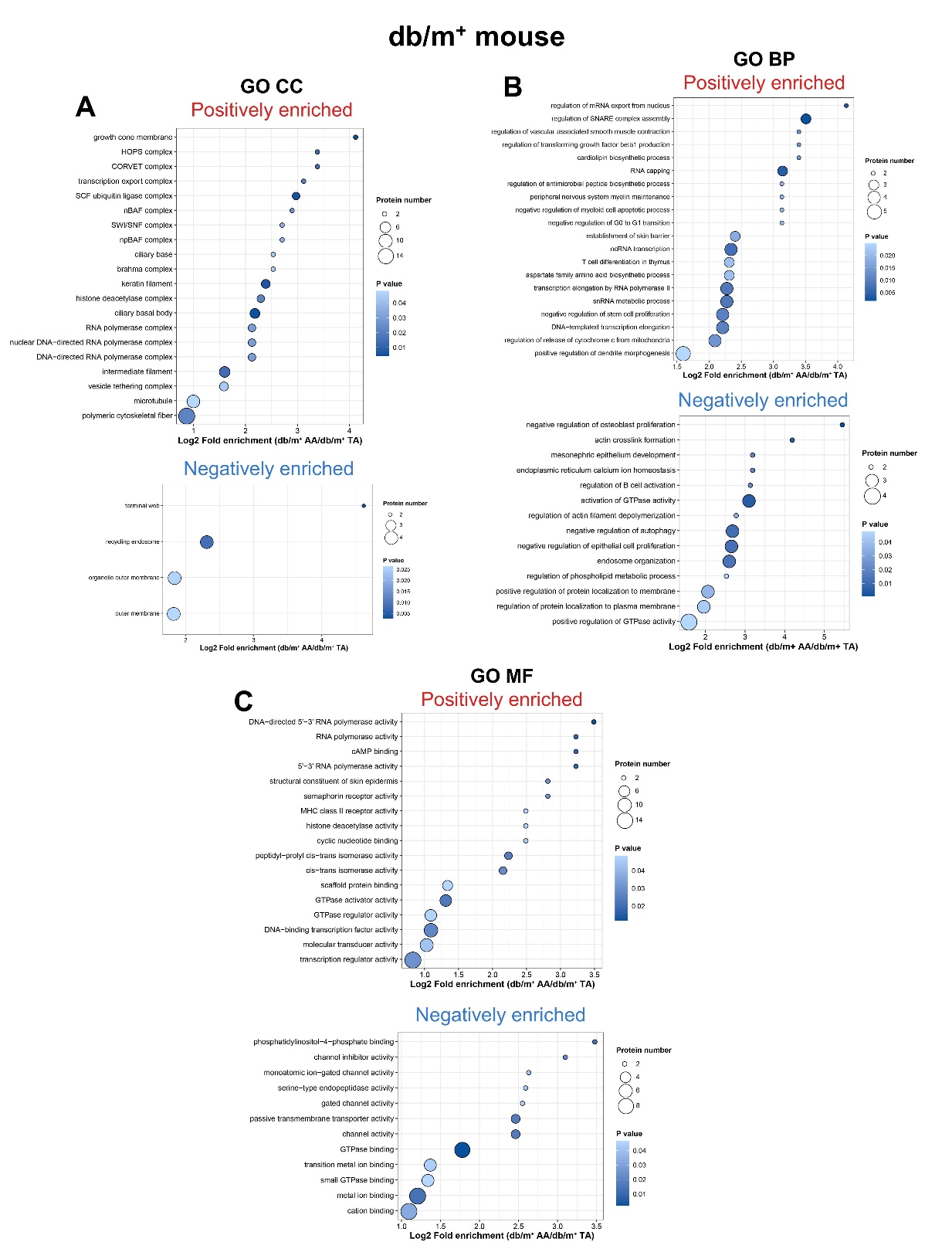


**Figure S17.** Positively and negatively enriched GO terms in db/m^+^ AA vs db/m^+^ TA, ranked by log2 fold enrichment. Dot plots display significantly enriched GO terms (FDR < 0.05) across three ontologies, including (**A**) CC, (**B**) BP, and (**C**) MF. AA, abdominal aorta; BP, biological process; CC, cellular component; FDR, false discovery rate; GO, Gene Ontology; MF, molecular function; TA, descending thoracic aorta.


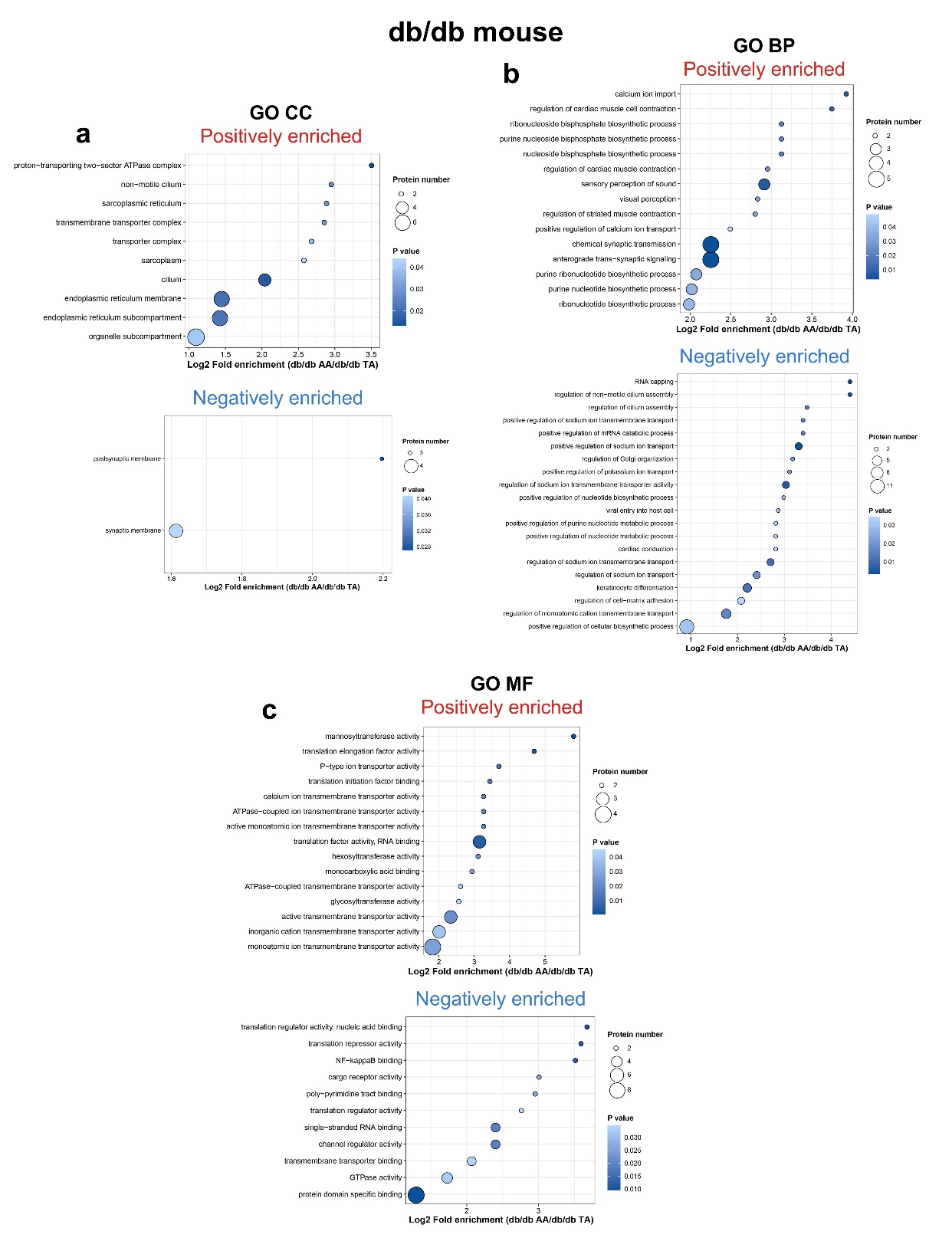


**Figure S18.** Positively and negatively enriched GO terms in db/db AA vs db/db TA, ranked by log2 fold enrichment. Dot plots display significantly enriched GO terms (FDR < 0.05) across three ontologies, including (**A**) CC, (**B**) BP, and (**C**) MF. AA, abdominal aorta; BP, biological process; CC, cellular component; FDR, false discovery rate; GO, Gene Ontology; MF, molecular function; TA, descending thoracic aorta.


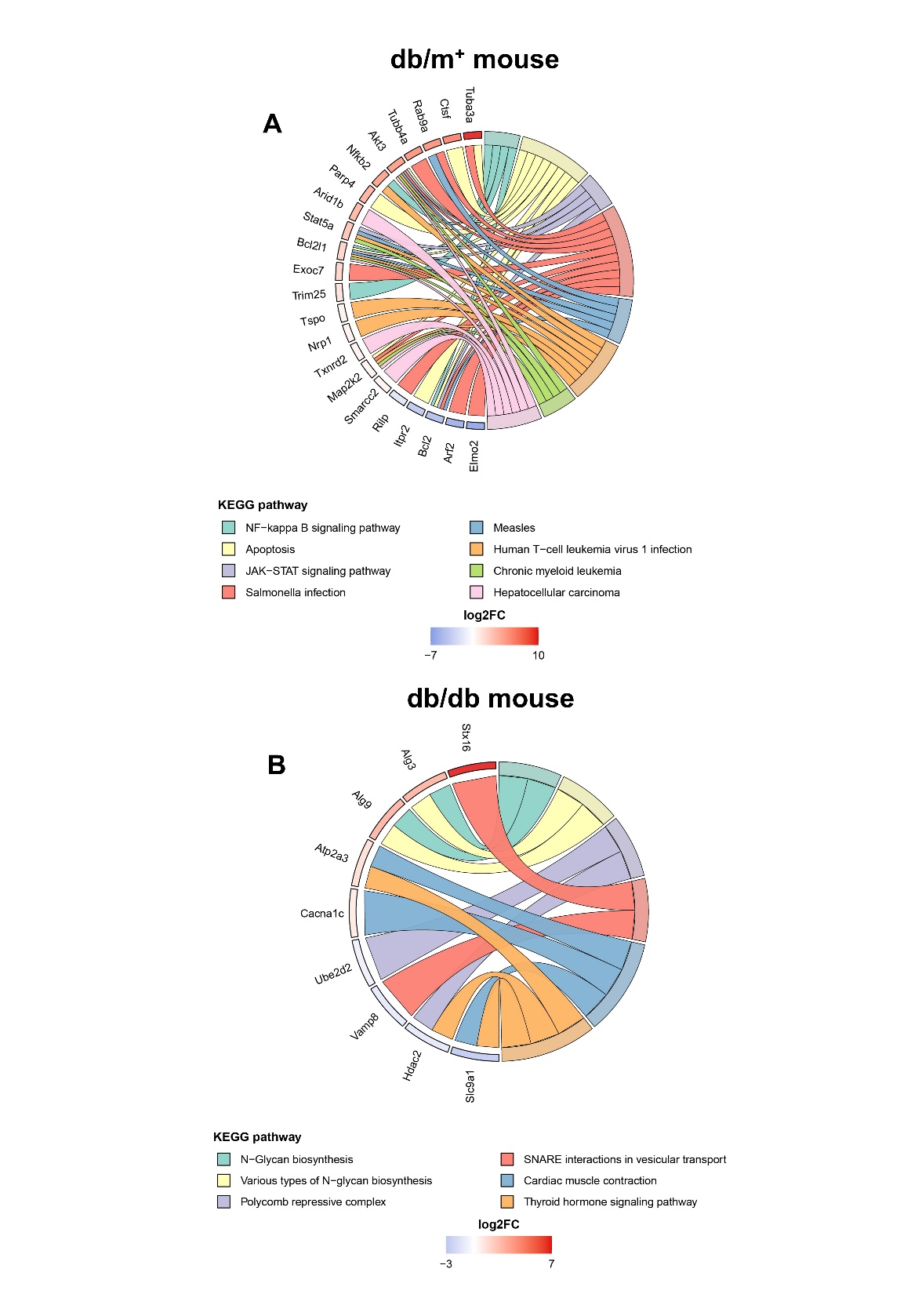


**Figure S19.** Chord diagrams linking significantly enriched KEGG pathways to DEPs, ranked by Log_2_FC, in AA vs TA of (**A**) db/m^+^ mice and (**B**) db/db mice. AA, abdominal aorta; DEP, differentially expressed protein; FC, fold change; KEGG, Kyoto Encyclopedia of Genes and Genomes; TA, descending thoracic aorta.


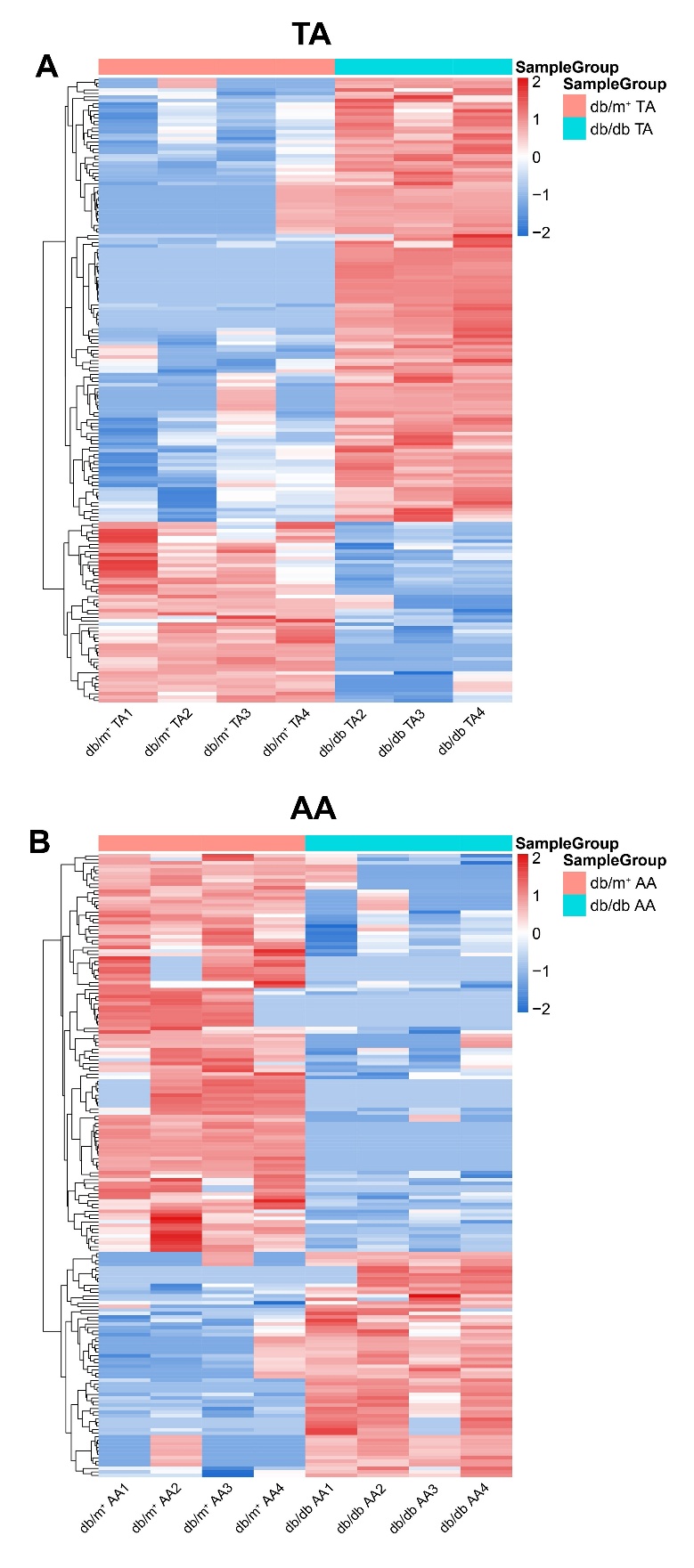


**Figure S20.** Heatmaps showing distinct clustering of (**A**) TA between db/m^+^ and db/db mice, and (**B**) AA between db/m^+^ and db/db mice. *n* = 4 for db/m^+^ TA; *n* = 3 for db/db TA; *n* = 4 for db/m^+^ AA; *n* = 4 for db/db AA. AA, abdominal aorta; TA, descending thoracic aorta.


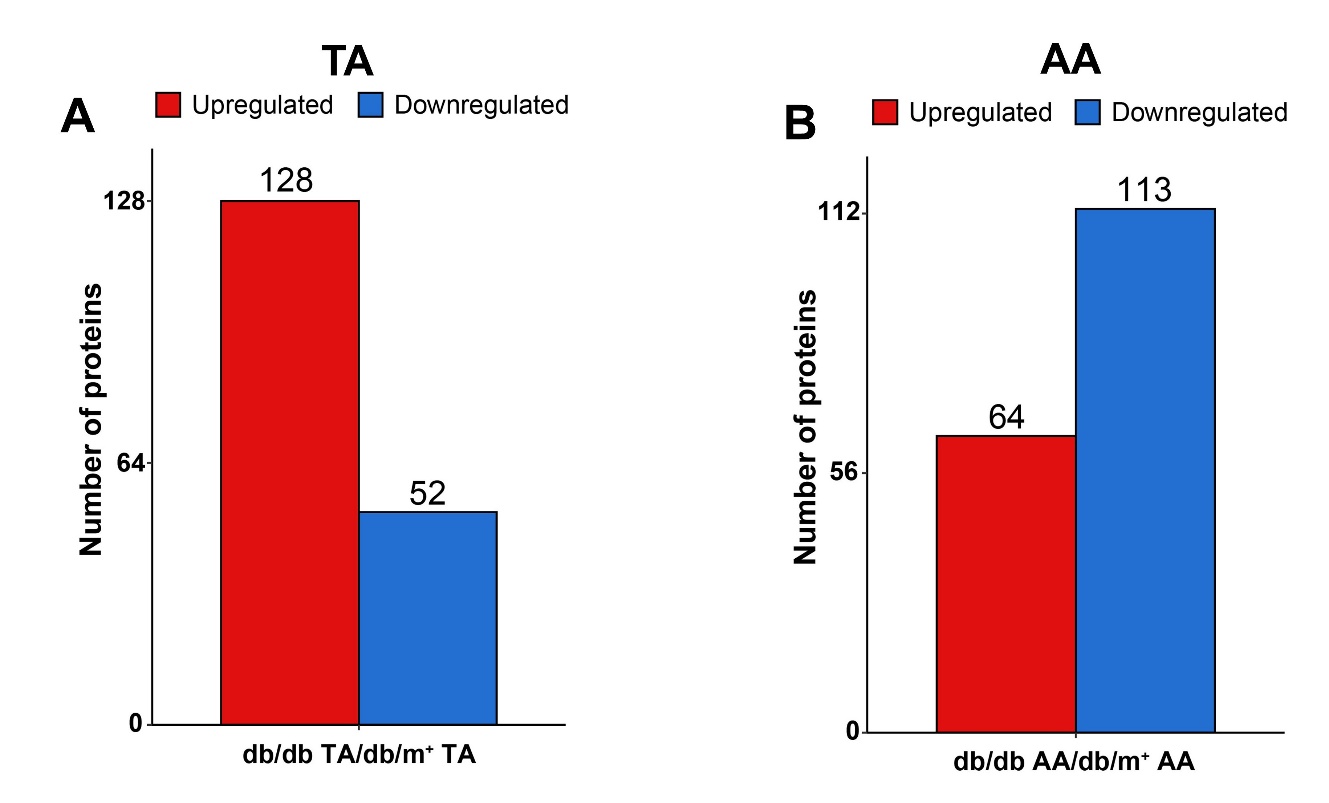


**Figure S21.** Number of upregulated and downregulated DEPs in (**A**) TA between db/m^+^ mice and db/db mice, and (**B**) AA between db/m^+^ mice and db/db mice. AA, abdominal aorta; DEP, differentially expressed protein; TA, descending thoracic aorta.


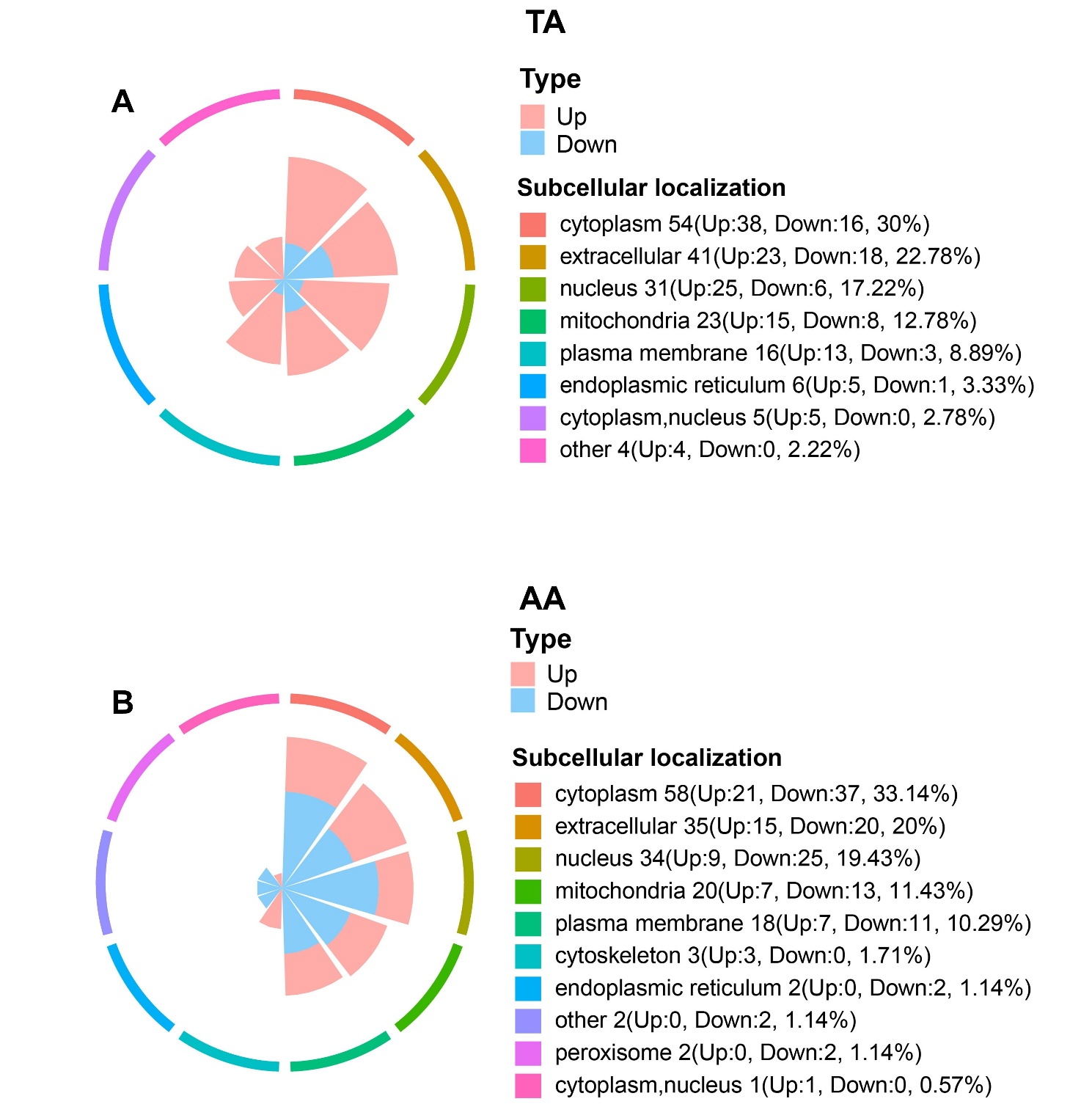


**Figure S22.** Nightingale rose diagram on subcellular localizations of upregulated and downregulated DEPs in (**A**) db/db TA vs db/m^+^ TA, and (**B**) db/db AA vs db/m^+^ AA. AA, abdominal aorta; DEP, differentially expressed protein; TA, descending thoracic aorta.


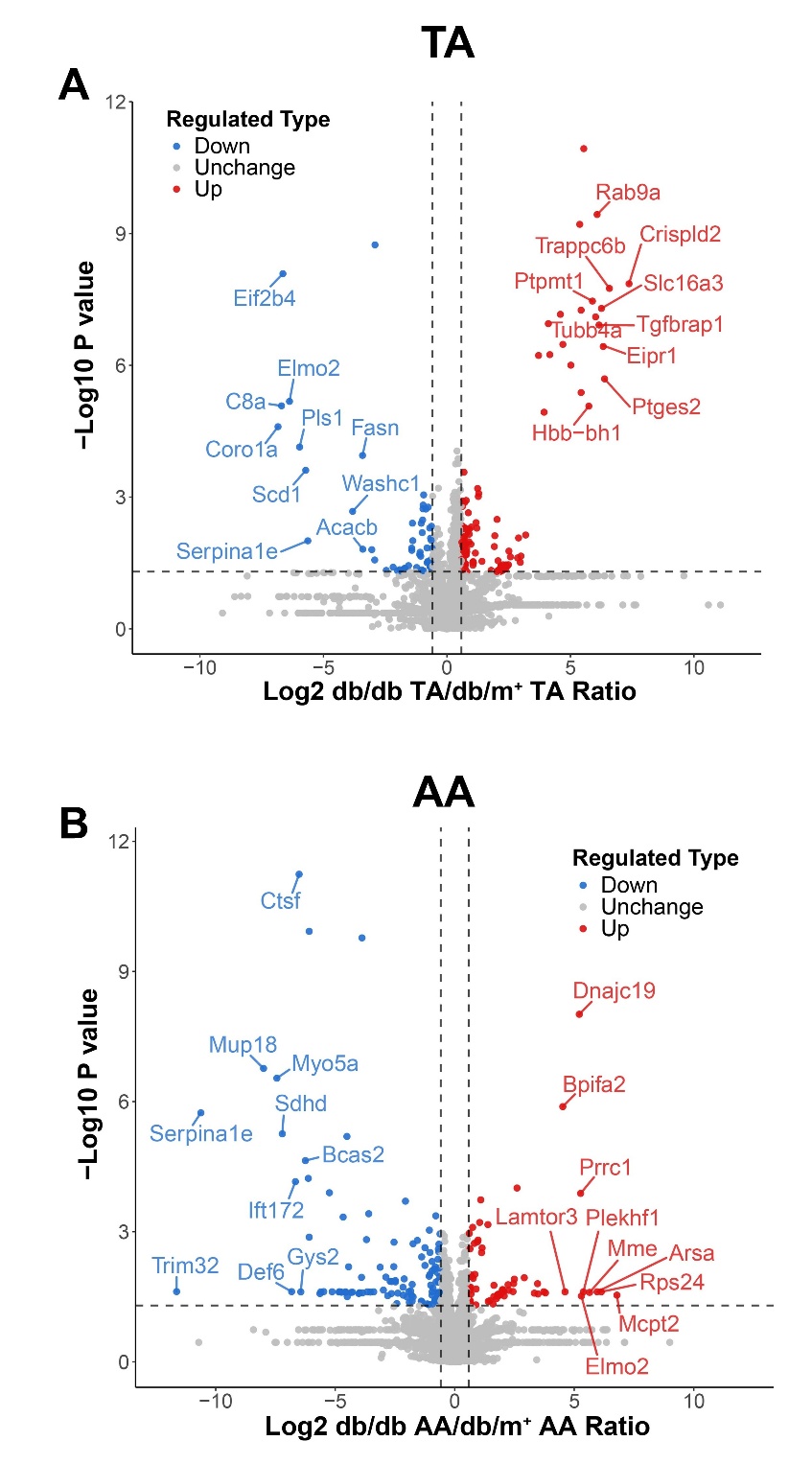


**Figure S23.** Volcano plots on DEPs in (**A**) TA between db/m^+^ and db/db mice, and (**B**) AA between db/m^+^ and db/db mice. Grey dotted lines: vertical at |Log_2_ FC| = 0.6 (~1.5-fold change) and horizontal at -log_10_ *p* = 1.3 (*p* < 0.05). AA, abdominal aorta; DEP, differentially expressed protein; TA, descending thoracic aorta.


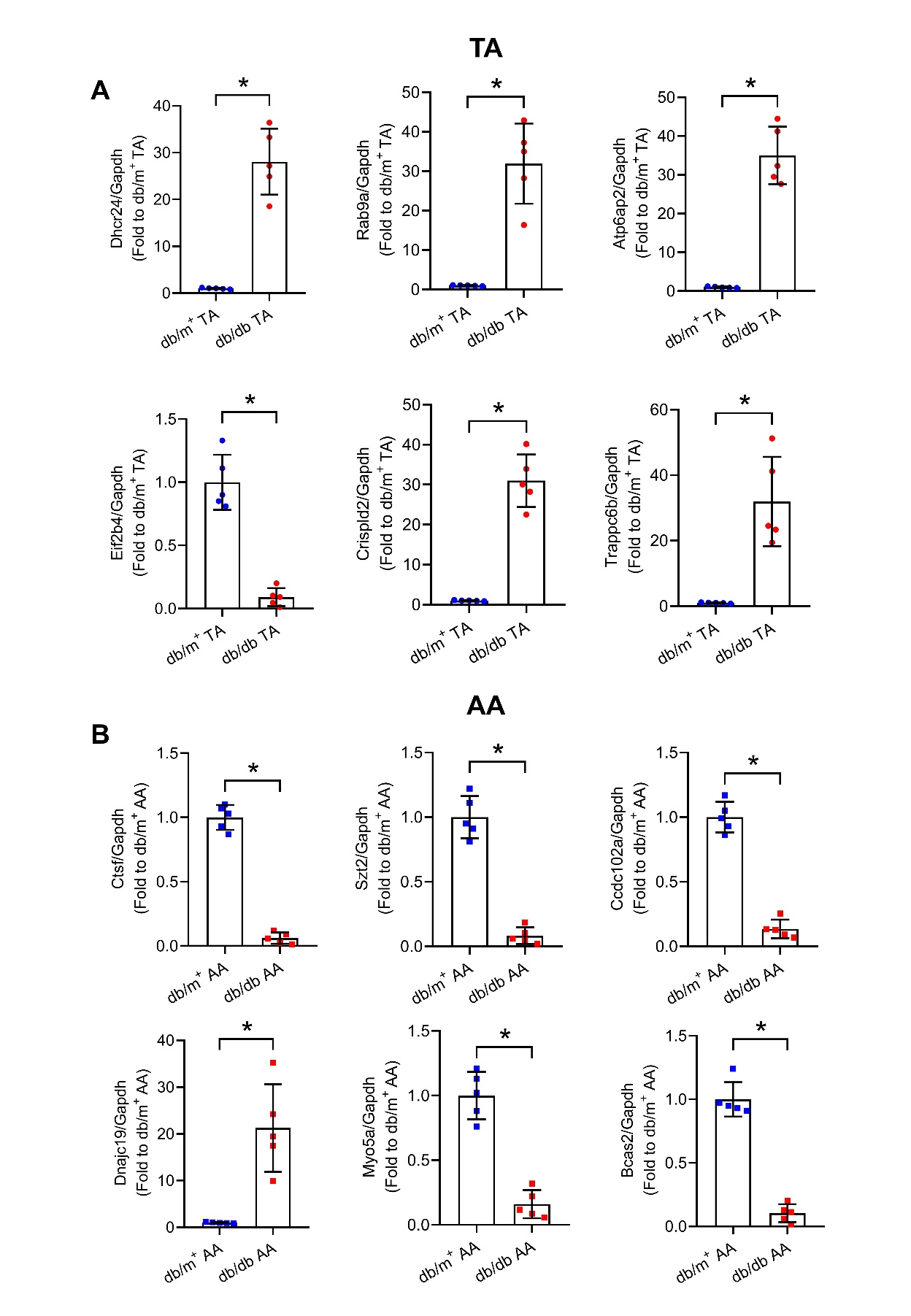


**Figure S24.** RT-PCR on candidate markers in (**A**) TA of db/db vs db/m^+^ mice, and (**B**) AA of db/db vs db/m^+^ mice. *n* = 5 per group. Data are presented as mean ± SD. **p* < 0.05 (unpaired *t*-tests and nonparametric Mann-Whitney tests). AA, abdominal aorta; TA, descending thoracic aorta.


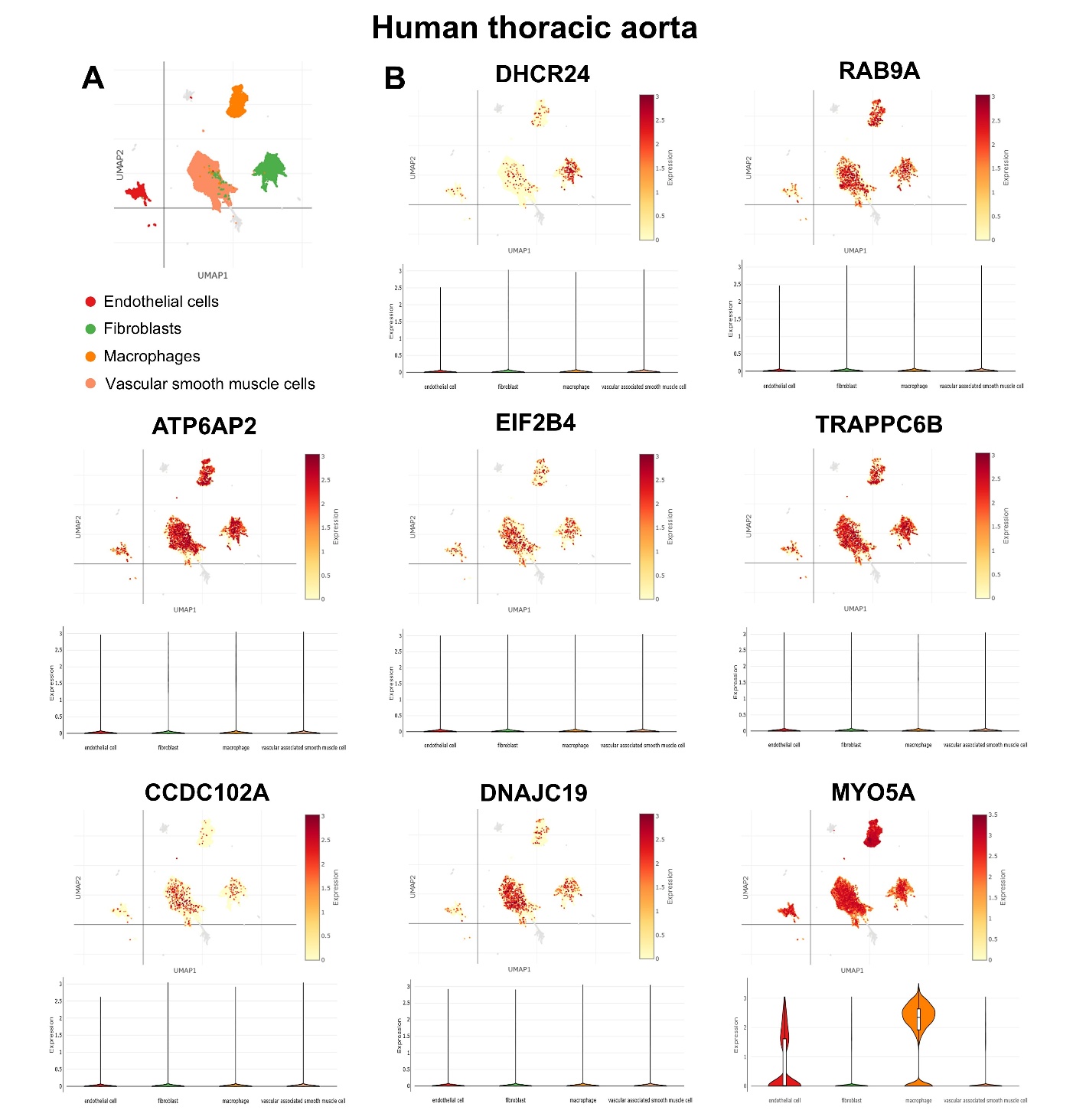


**Figure S25.** Cellular distribution and expression of selected marker genes from mouse TA and AA in human thoracic aorta based on snRNA-seq data. (**A**) UMAP visualization of 50133 individual nuclei from thoracic aortas of 3 individuals. (**B**) UMAP plots showing the distribution and violin plots showing the expression of selected marker genes in different vascular cell types. AA, abdominal aorta; snRNA-seq, single-nucleus RNA sequencing; TA, descending thoracic aorta; UMAP, uniform manifold approximation and projection.


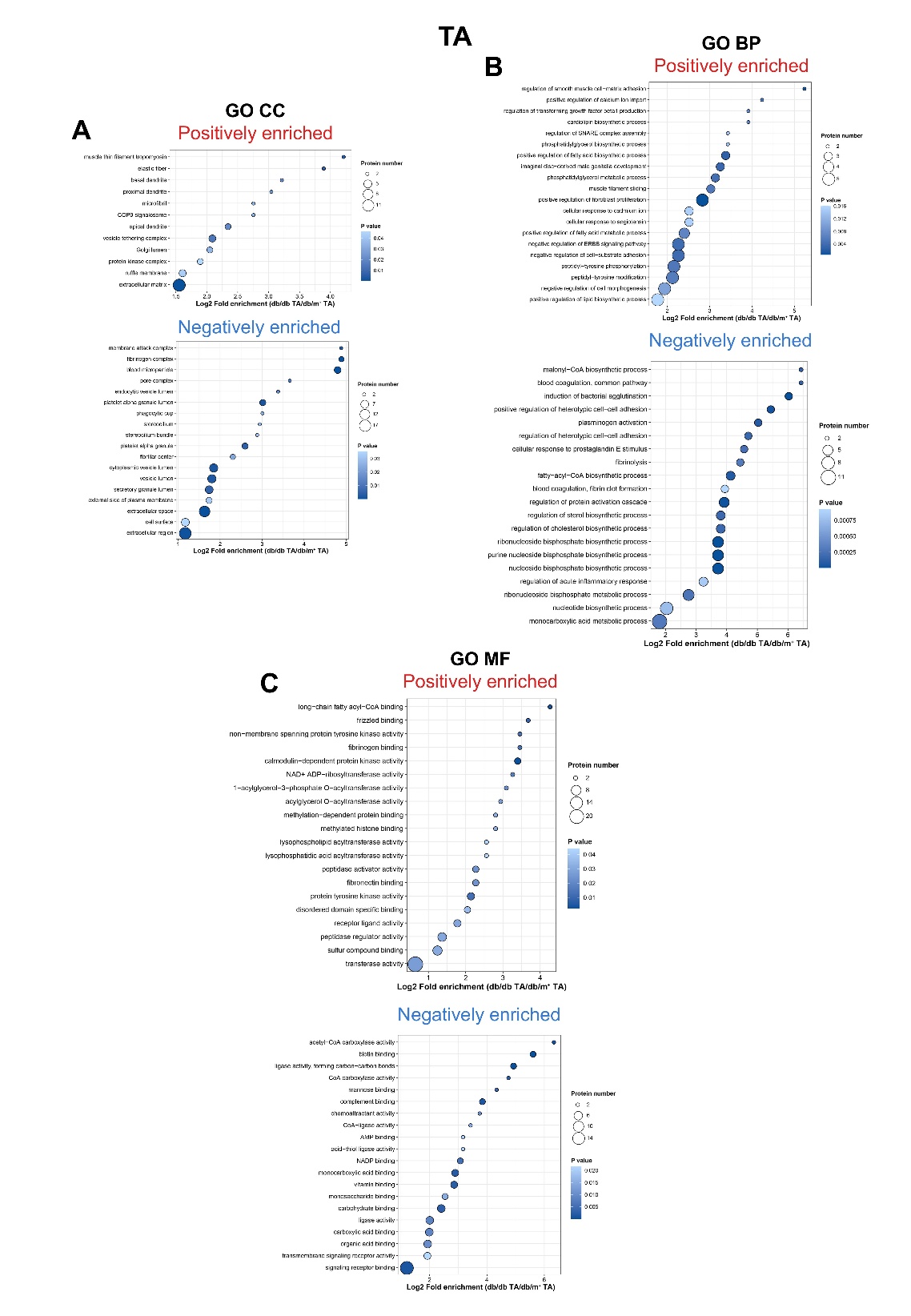


**Figure S26.** Positively and negatively enriched GO terms in db/db TA vs db/m^+^ TA, ranked by log2 fold enrichment. Dot plots display significantly enriched GO terms (FDR < 0.05) across three ontologies, including (**A**) CC, (**B**) BP, and (**C**) MF. BP, biological process; CC, cellular component; FDR, false discovery rate; GO, Gene Ontology; MF, molecular function; TA, descending thoracic aorta.


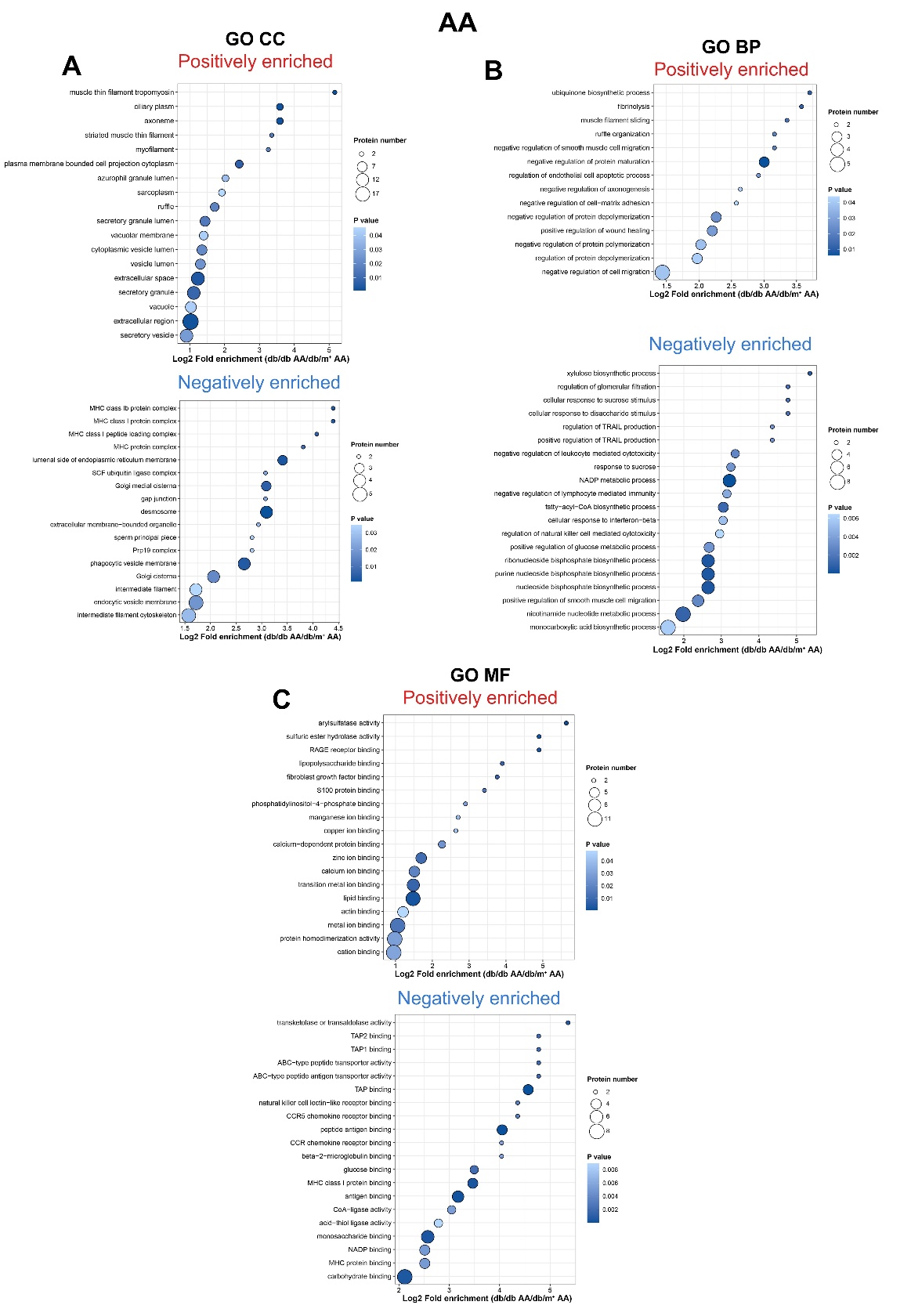


**Figure S27.** Positively and negatively enriched GO terms in db/db AA vs db/m^+^ AA, ranked by log2 fold enrichment. Dot plots display significantly enriched GO terms (FDR < 0.05) across three ontologies, including (**A**) CC, (**B**) BP, and (**C**) MF. AA, abdominal aorta; BP, biological process; CC, cellular component; FDR, false discovery rate; GO, Gene Ontology; MF, molecular function.


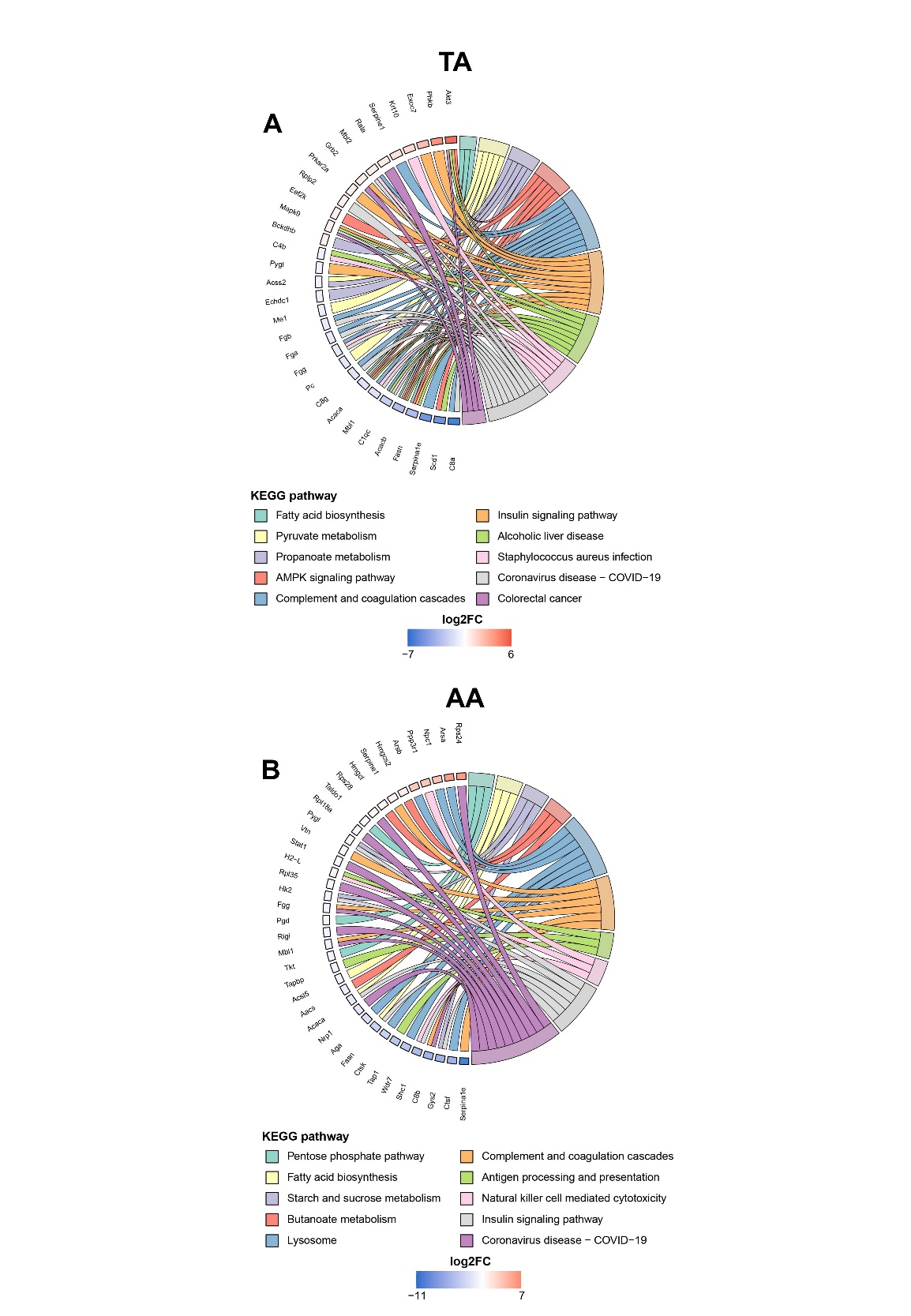


**Figure S28.** Chord diagrams linking significantly enriched KEGG pathways to DEPs, ranked by Log_2_FC, in (**A**) db/db TA vs db/m^+^ TA, and (**B**) db/db AA vs db/m^+^ AA. AA, abdominal aorta; DEP, differentially expressed protein; FC, fold change; KEGG, Kyoto Encyclopedia of Genes and Genomes; TA, descending thoracic aorta.


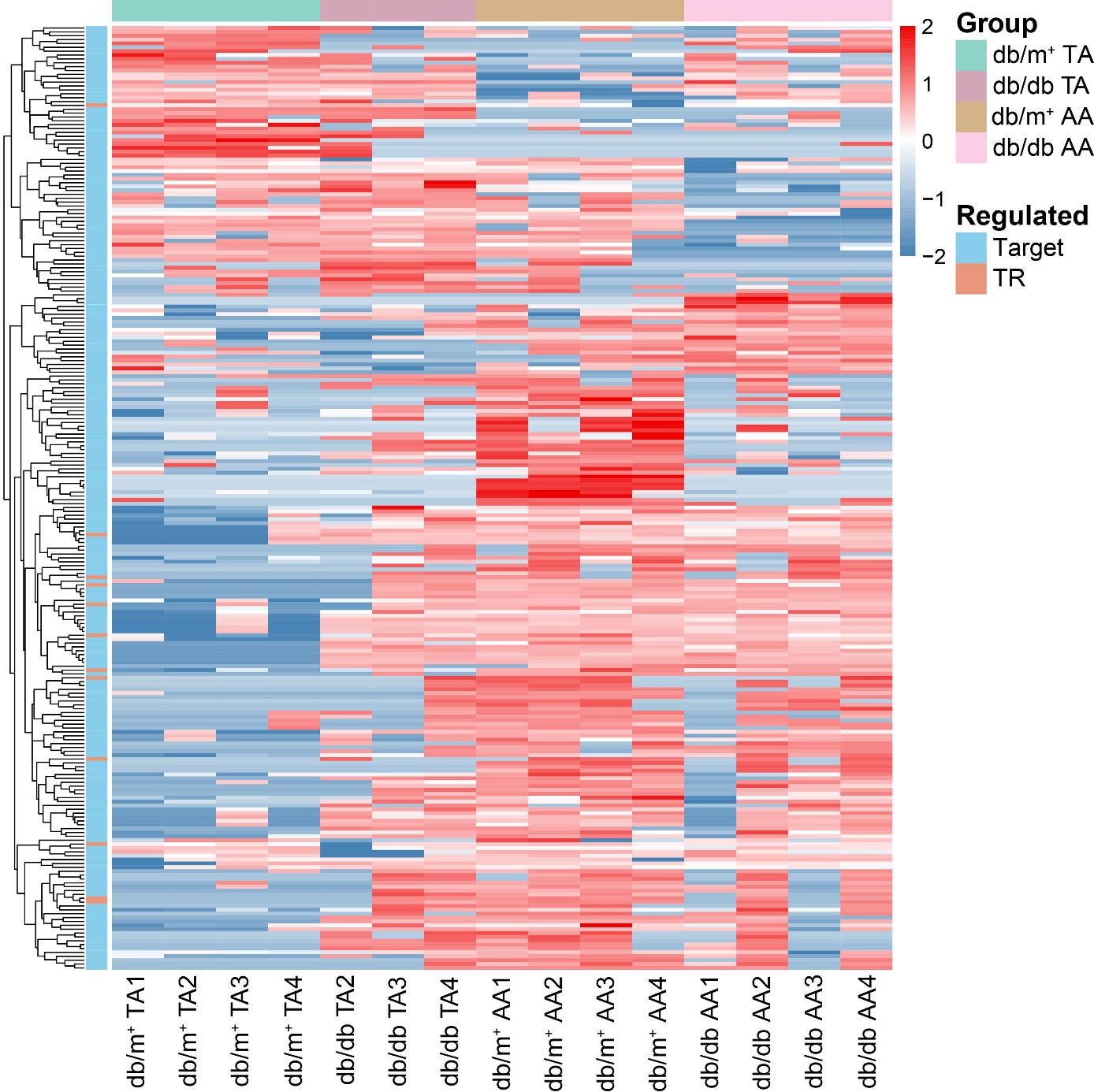


**Figure S29.** Heatmap showing distinct clustering of TRs and TR targets between the four aortic groups. *n* = 4 for db/m^+^ TA; *n* = 3 for db/db TA; *n* = 4 for db/m^+^ AA; *n* = 4 for db/db AA. AA, abdominal aorta; TA, descending thoracic aorta; TR, transcriptional regulator.


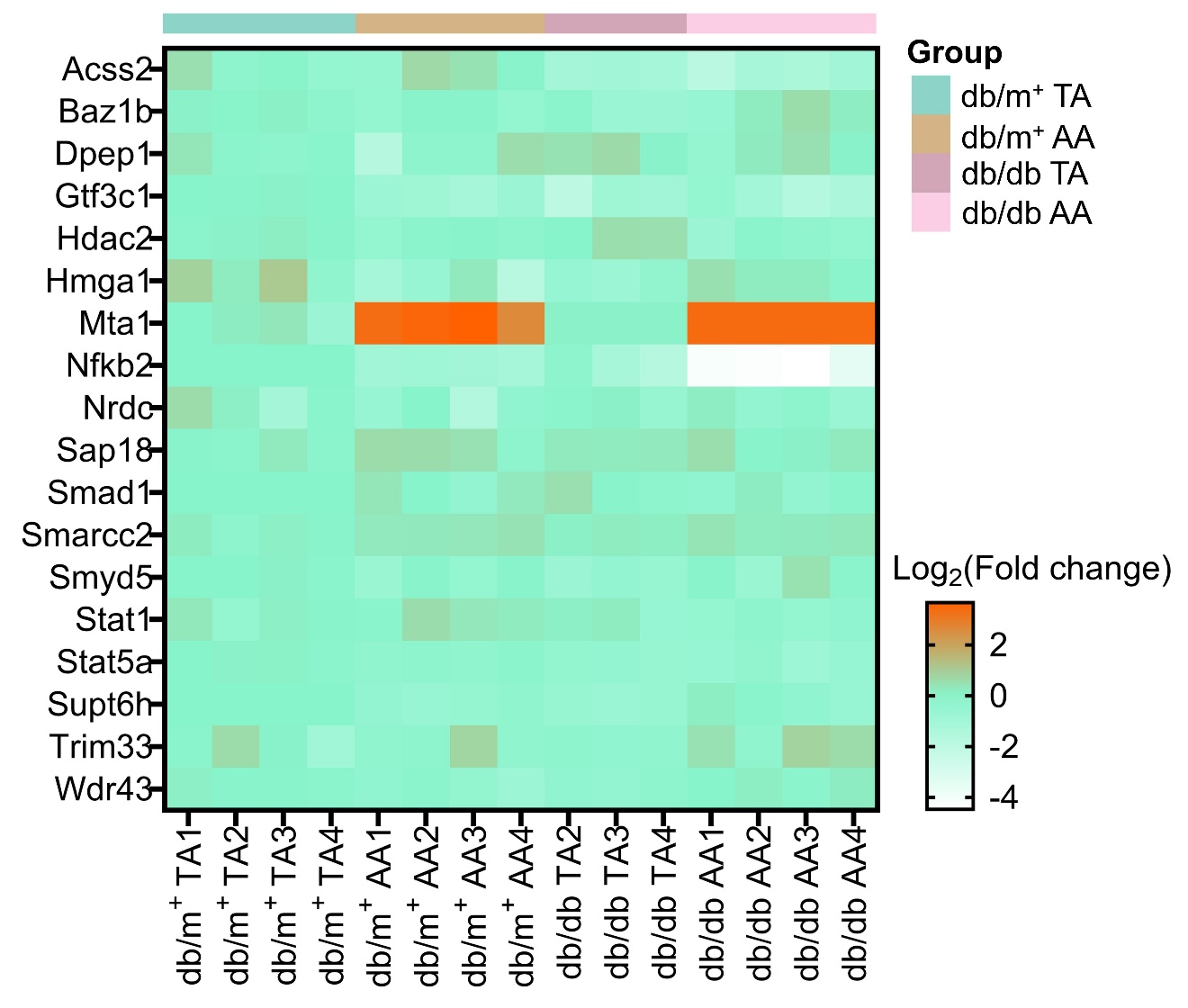


**Figure S30.** Heatmap depicting the relative protein expression of the 18 differentially expressed TRs among the four aortic groups. *n* = 4 for db/m^+^ TA; *n* = 4 for db/m^+^ AA; *n* = 3 for db/db TA; *n* = 4 for db/db AA. AA, abdominal aorta; TA, descending thoracic aorta; TR, transcriptional regulator.


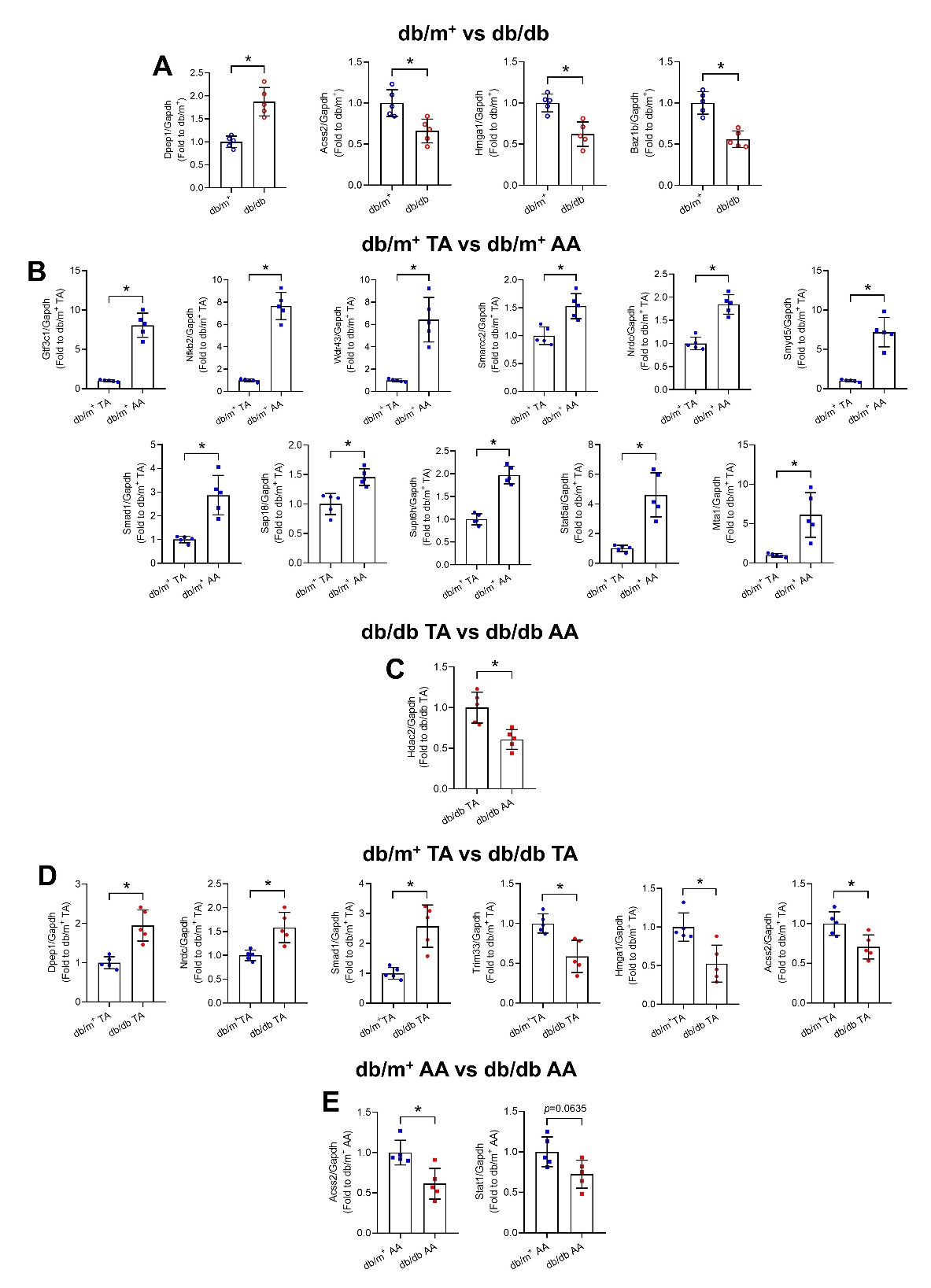


**Figure S31.** RT-PCR on 18 differentially expressed TRs in TA and AA of non-diabetic and diabetic mice among different group comparisons, including (**a**) db/m^+^ vs db/db, (**b**) db/m^+^ TA vs db/m^+^ AA, (**c**) db/db TA vs db/db AA, (**d**) db/m^+^ TA vs db/db TA, and (**e**) db/m^+^ AA vs db/db AA. *n* = 5 per group. Data are presented as mean ± SD. **p* < 0.05 (unpaired *t*-tests and nonparametric Mann-Whitney tests). AA, abdominal aorta; TA, descending thoracic aorta; TR, transcriptional regulator.


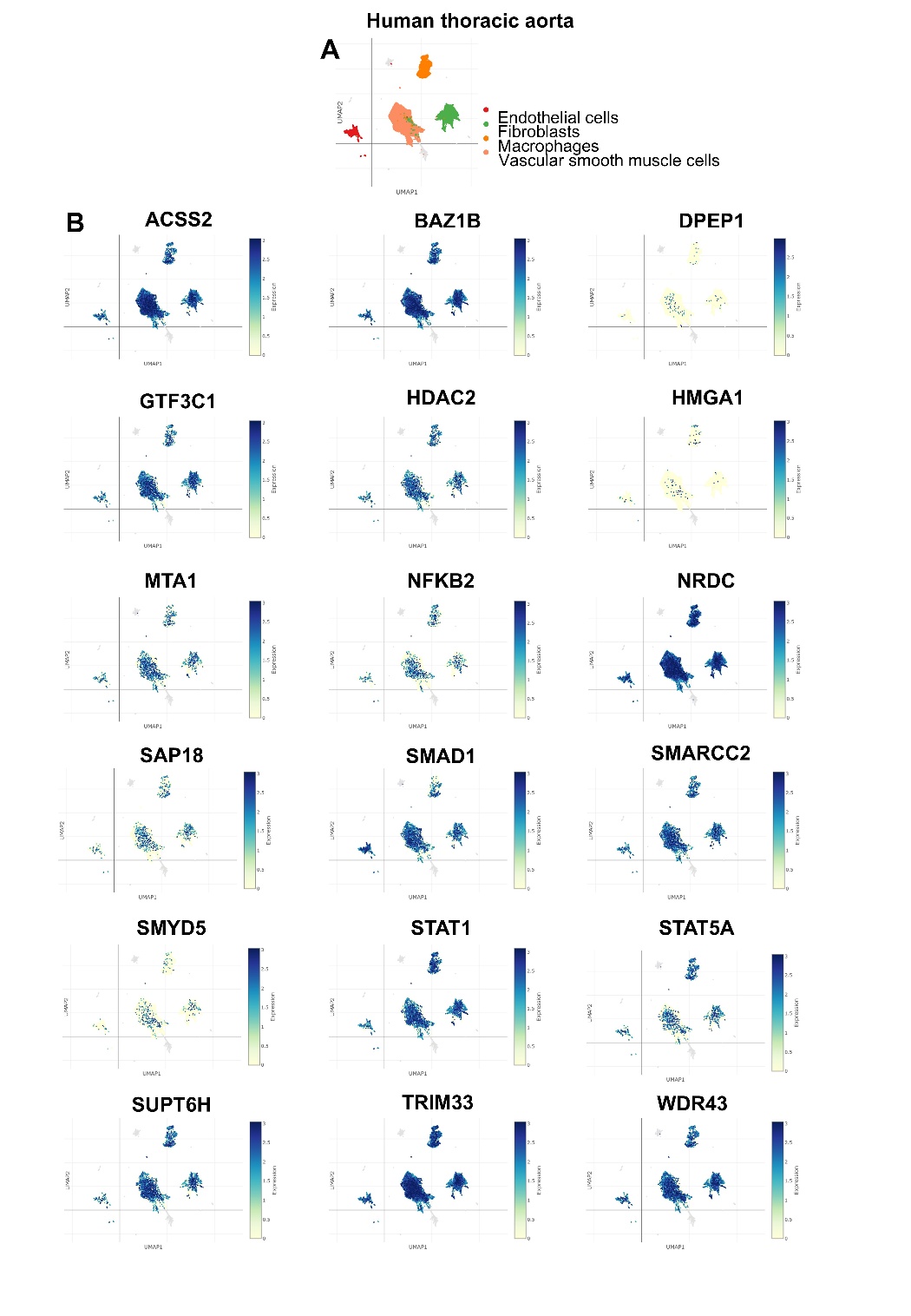


**Figure S32.** Cellular distribution of the 18 differentially expressed TRs from mouse TA and AA in human thoracic aorta based on snRNA-seq data. (**A**) UMAP visualization of 50133 individual nuclei from thoracic aortas of 3 individuals. (**B**) UMAP plots showing the distribution of the 18 TRs in different vascular cell types. AA, abdominal aorta; snRNA-seq, single-nucleus RNA sequencing; TA, descending thoracic aorta; TR, transcriptional regulator; UMAP, uniform manifold approximation and projection.


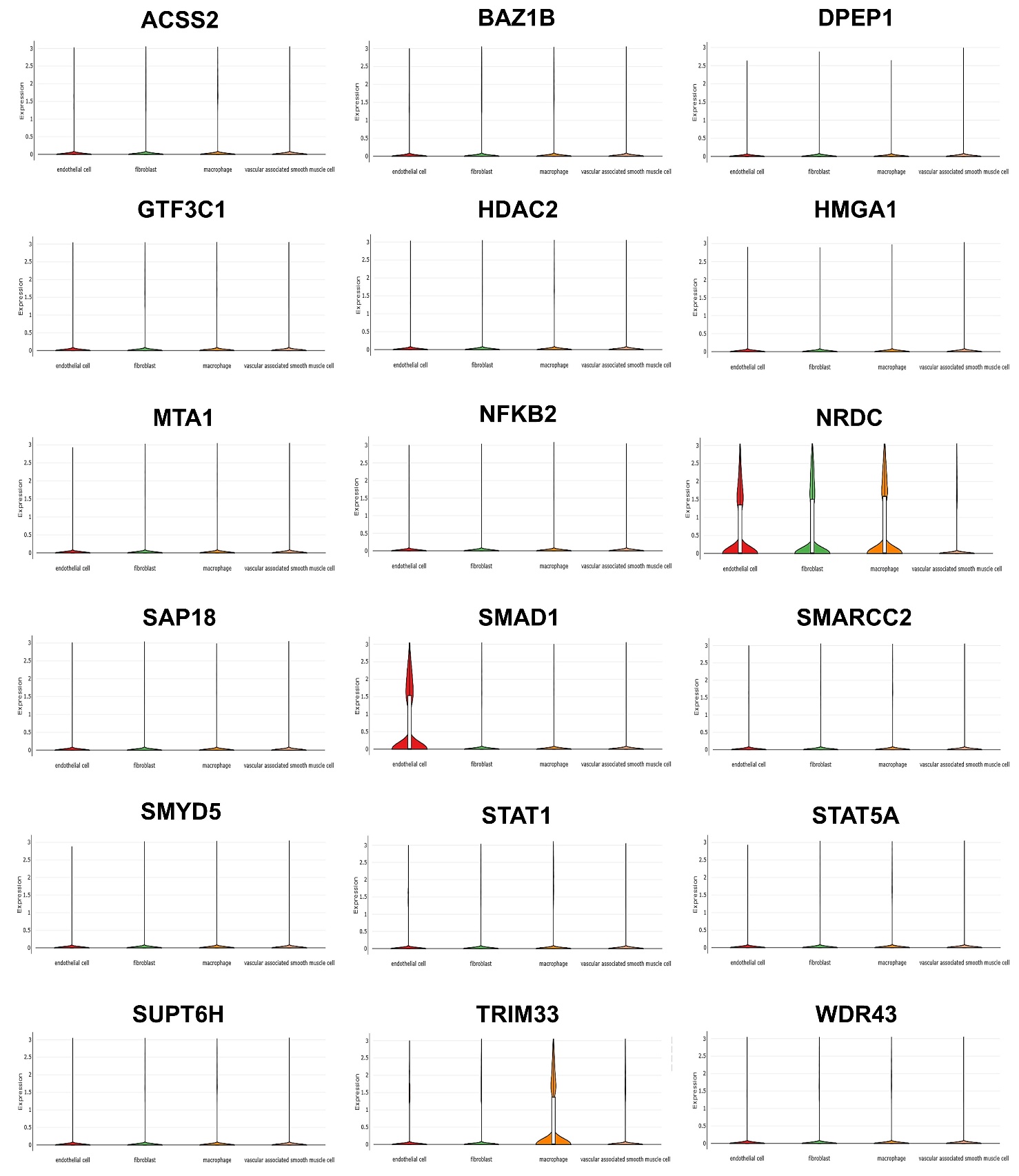


**Figure S33.** Violin plots showing the expression of the 18 TRs in different vascular cell types from mouse TA and AA in human thoracic aorta based on snRNA-seq data. AA, abdominal aorta; snRNA-seq, single-nucleus RNA sequencing; TA, descending thoracic aorta; TR, transcriptional regulator.


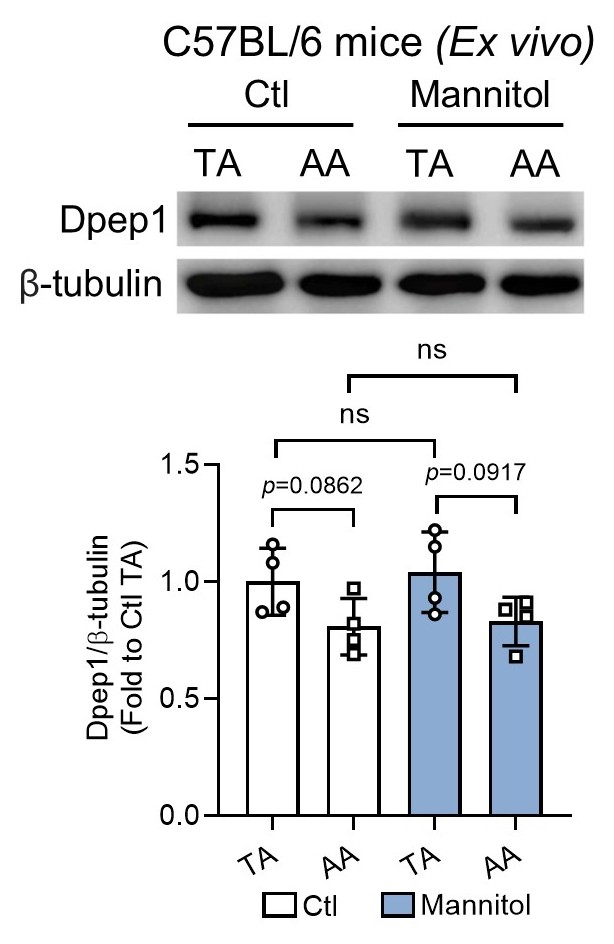


**Figure S34.** Western blotting on Dpep1 expression in TA and AA from C57BL/6 mice following *ex vivo* treatment with mannitol. n = 4 per group. Data are presented as mean ± SD. Brown-Forsythe and Welch ANOVA, and unpaired t with Welch’s correction. AA, abdominal aorta; Dpep1, dipeptidase 1; TA, descending thoracic aorta.

**
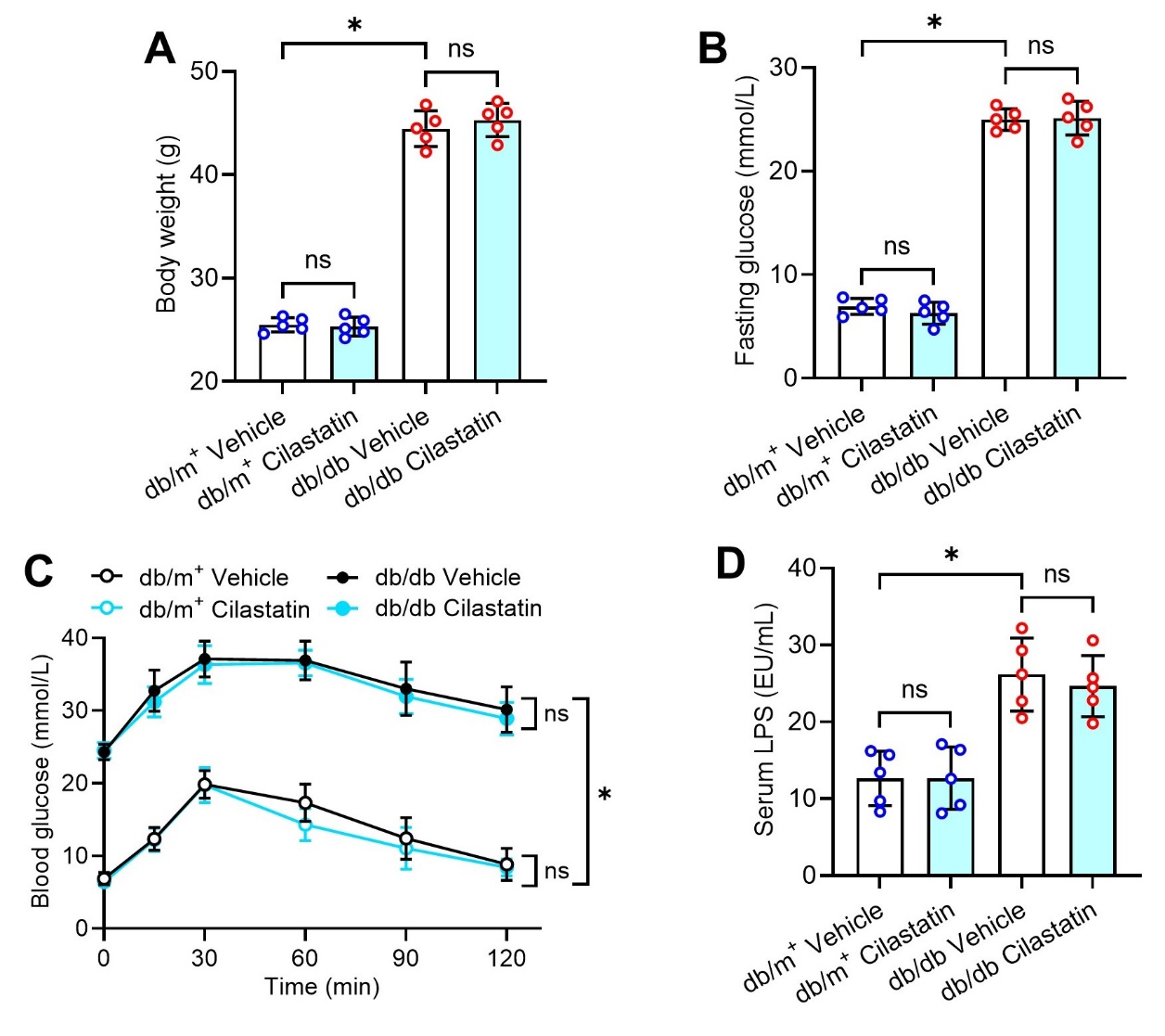
**

**Figure S35.** Effects of 4-week cilastatin treatment on body weights and blood glucose levels of non-diabetic and diabetic mice. (**A**) Body weights, (**B**) fasting glucose levels, (**C**) OGTT, and (**D**) serum LPS levels of db/m^+^ and db/db mice after 4-week cilastatin treatment (*n* = 5 per group). Data are presented as mean ± SD. **p* < 0.05 (Brown-Forsythe and Welch ANOVA, and unpaired t with Welch’s correction). LPS, lipopolysaccharide; OGTT, oral glucose tolerance test.

**
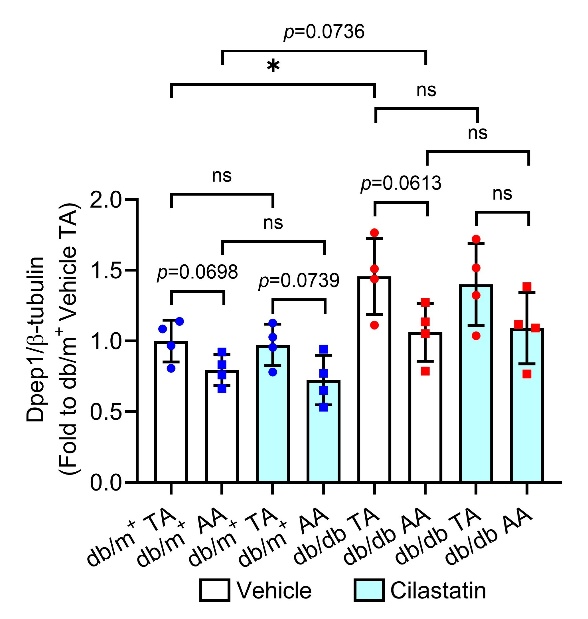
**

**Figure S36.** Quantification on Western blotting results of Dpep1 expression in aortic segments of cilastatin-treated db/m^+^ and db/db mice. *n* = 4 per group. Data are presented as mean ± SD. **p* < 0.05 (Brown-Forsythe and Welch ANOVA, and unpaired t with Welch’s correction). AA, abdominal aorta; Dpep1, dipeptidase 1; TA, descending thoracic aorta.


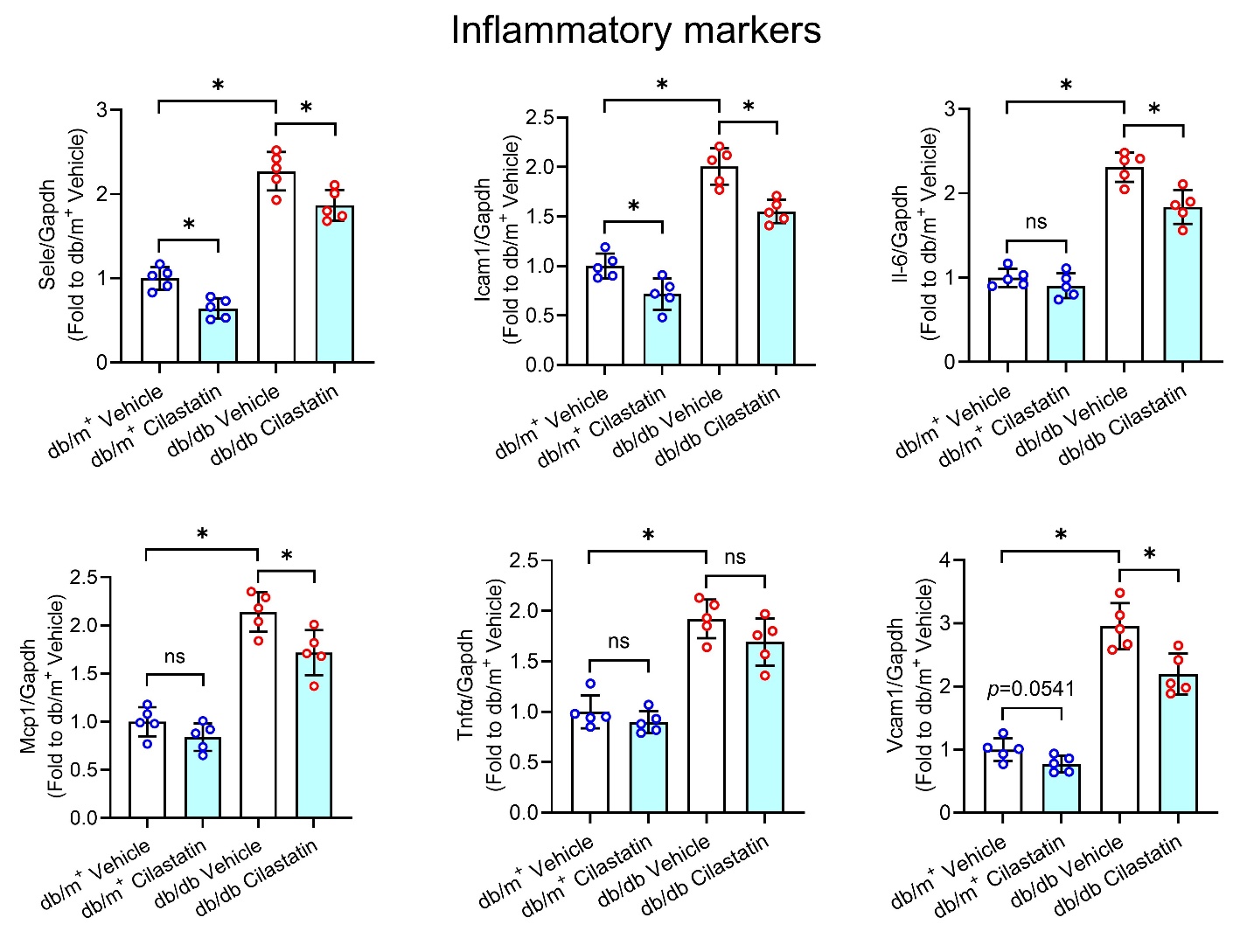


**Figure S37.** RT-PCR on inflammatory markers of mouse aortas after chronic cilastatin treatment. *n* = 5 per group. Data are presented as mean ± SD. **p* < 0.05 (Brown-Forsythe and Welch ANOVA, and unpaired t with Welch’s correction).

**
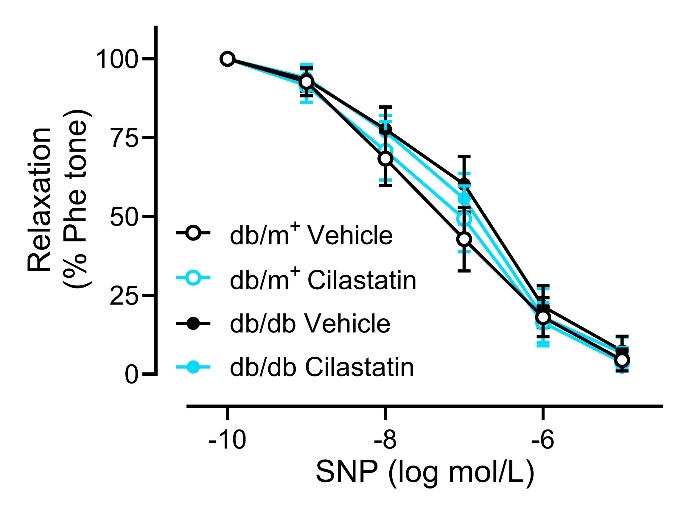
**

**Figure S38.** Endothelium-independent relaxations of diabetic mouse aortas upon chronic cilastatin treatment. *n* = 5 per group. Data are presented as mean ± SD. Brown-Forsythe and Welch ANOVA, and unpaired t with Welch’s correction. Phe, phenylephrine; SNP, sodium nitroprusside.

**
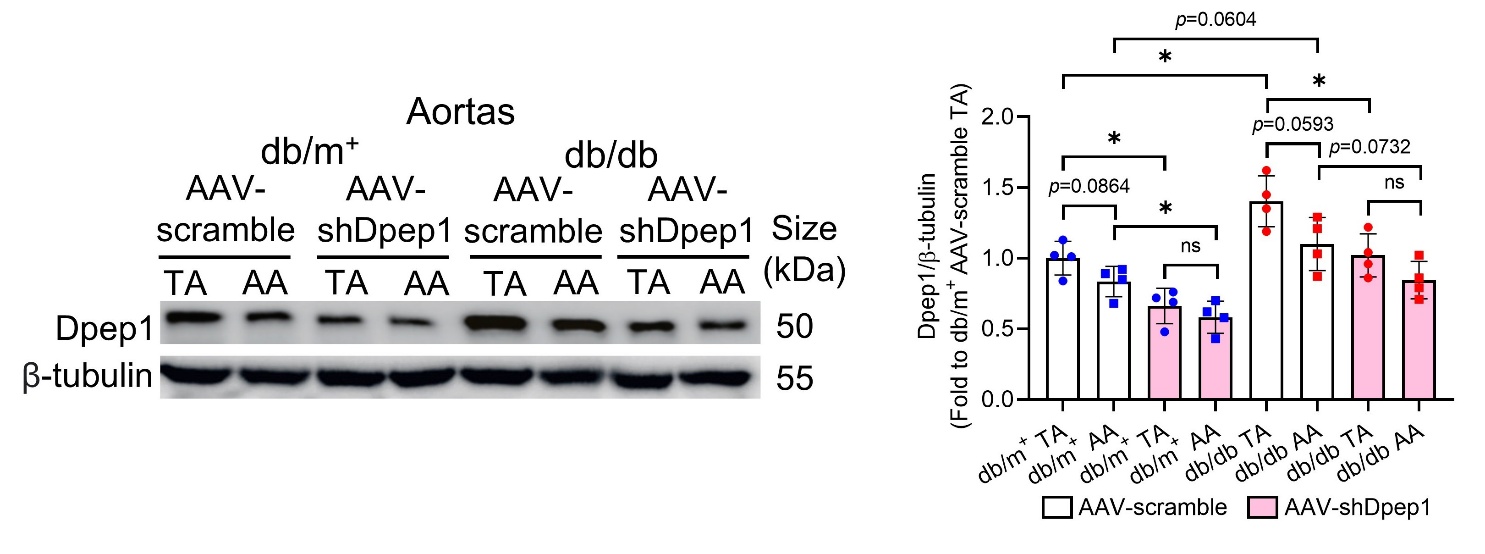
**

**Figure S39.** Western blotting on Dpep1 expression in thoracic and abdominal aortas of non-diabetic and diabetic mice upon injection of AAV-shDpep1. *n* = 4 per group. Data are presented as mean ± SD. *p < 0.05 (Brown-Forsythe and Welch ANOVA, and unpaired t with Welch’s correction). AA, abdominal aorta; AAV, adeno-associated virus; Dpep1, dipeptidase 1; TA, descending thoracic aorta.

**
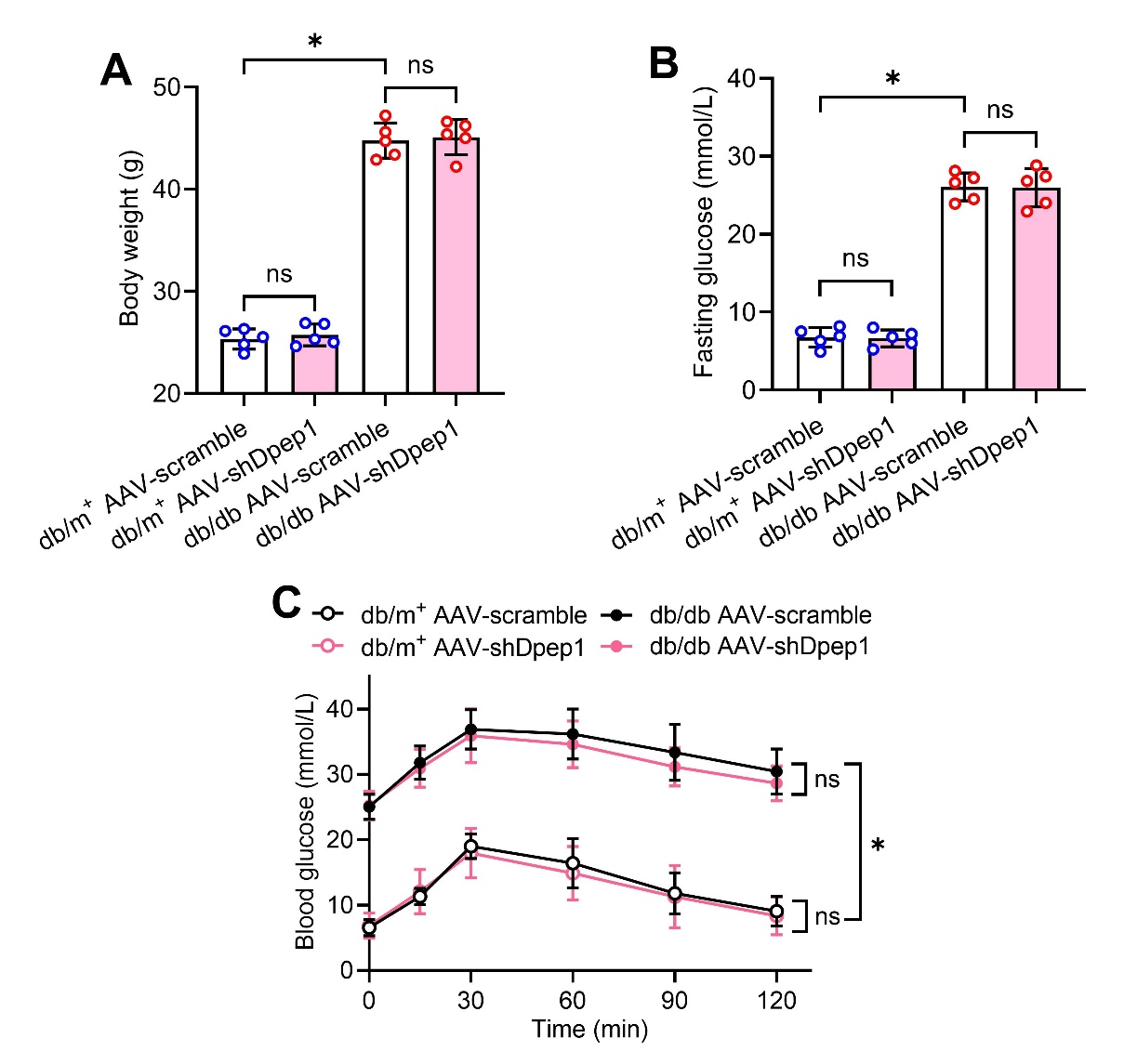
**

**Figure S40.** Effects of AAV-mediated Dpep1 knockdown on body weights and blood glucose levels of non-diabetic and diabetic mice. (**A**) Body weights, (**B**) fasting glucose levels, and (**C**) OGTT of db/m^+^ and db/db mice after injection of AAV-shDpep1 (*n* = 5 per group). Data are presented as mean ± SD. **p* < 0.05 (Brown-Forsythe and Welch ANOVA, and unpaired t with Welch’s correction). AAV, adeno-associated virus; Dpep1, dipeptidase 1; OGTT, oral glucose tolerance test.

**
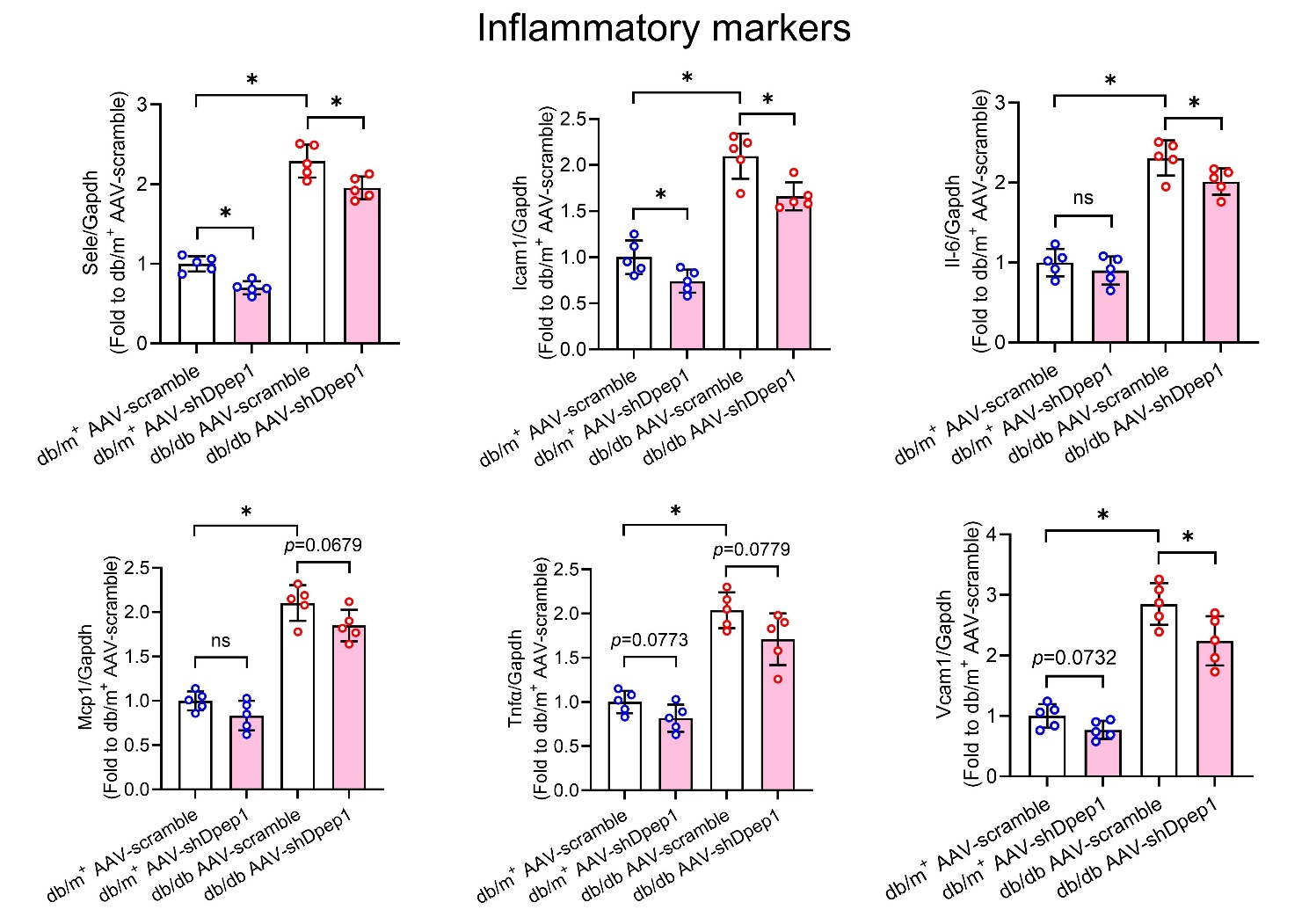
**

**Figure S41.** RT-PCR on inflammatory markers of mouse aortas upon AAV-mediated Dpep1 knockdown. *n* = 5 per group. Data are presented as mean ± SD. **p* < 0.05 (Brown-Forsythe and Welch ANOVA, and unpaired t with Welch’s correction). AAV, adeno-associated virus; Dpep1, dipeptidase 1.

**
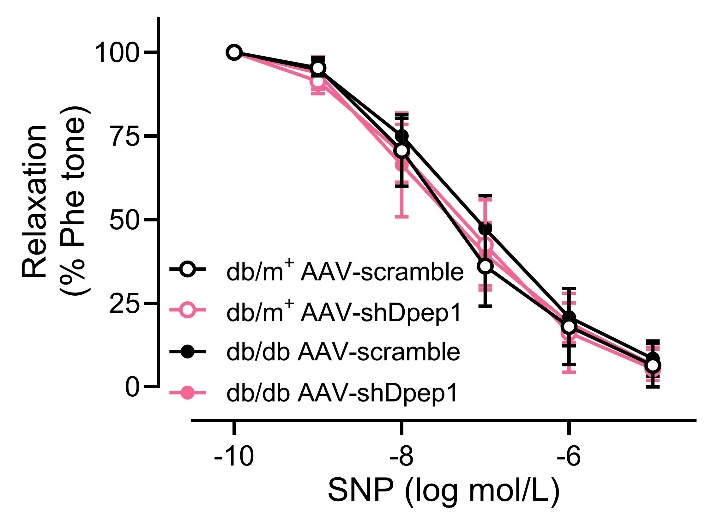
**

**Figure S42.** Endothelium-independent relaxations of diabetic mouse aortas upon AAV-mediated Dpep1 knockdown. *n* = 5 per group. Data are presented as mean ± SD. Brown-Forsythe and Welch ANOVA, and unpaired t with Welch’s correction. AAV, adeno-associated virus; Dpep1, dipeptidase 1; Phe, phenylephrine; SNP, sodium nitroprusside.

**
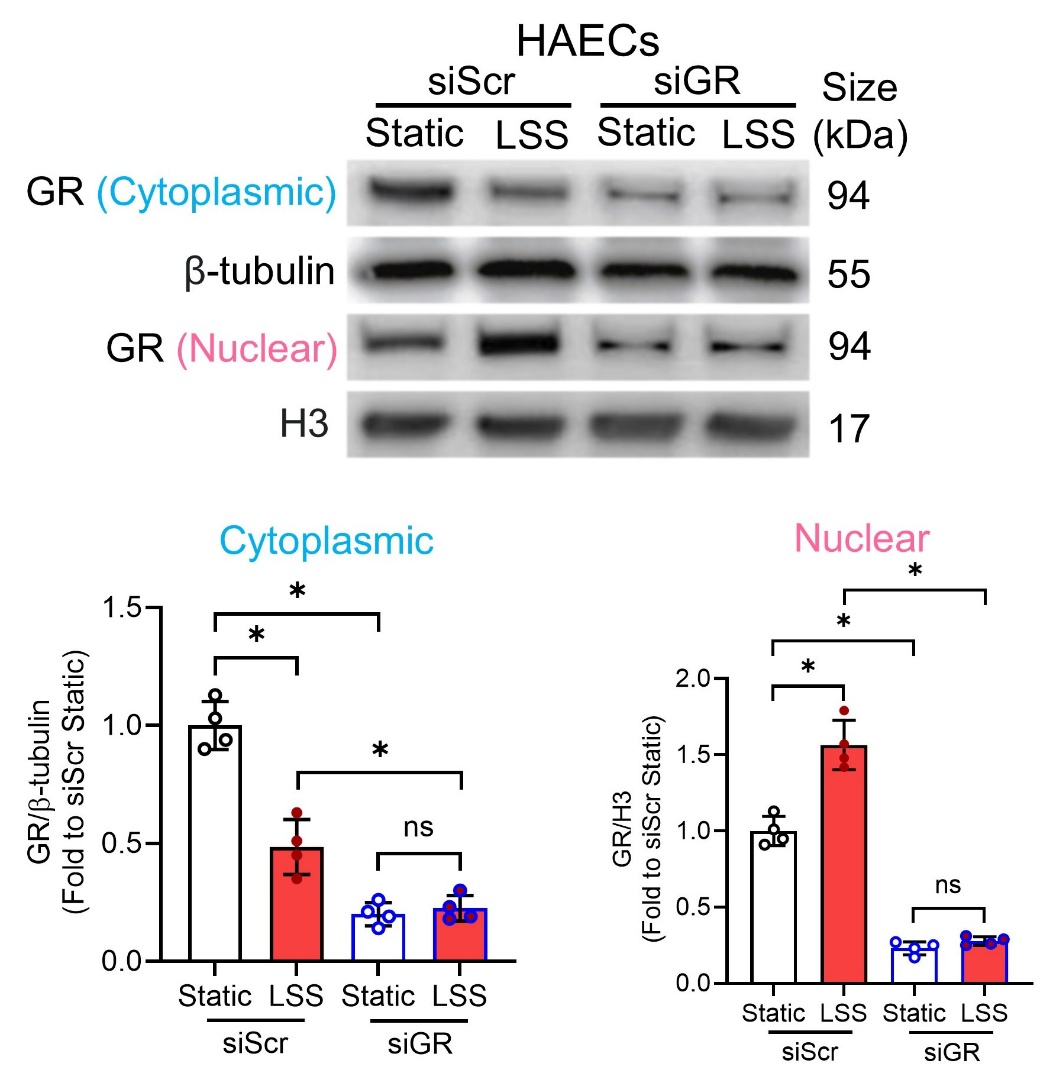
**

**Figure S43.** Western blotting on GR expression in nuclear/cytoplasmic fractions of HAECs upon GR knockdown and laminar flow exposure. *n* = 4 per group. Data are presented as mean ± SD. Brown-Forsythe and Welch ANOVA, and unpaired t with Welch’s correction. GR, glucocorticoid receptor; HAEC, human aortic endothelial cell; LSS, laminar shear stress; siScr, scrambled siRNA.

**
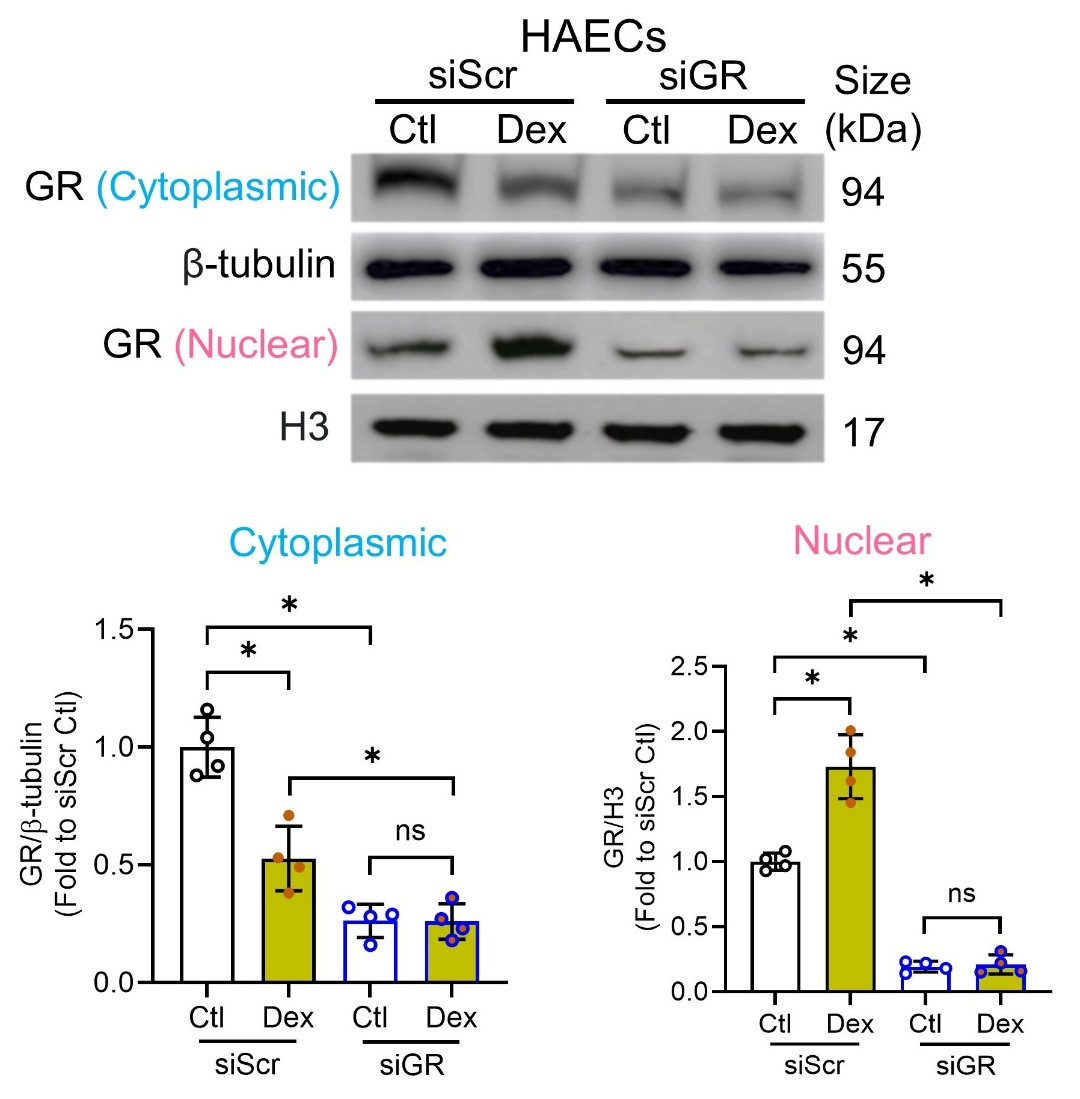
**

**Figure S44.** Western blotting on GR expression in nuclear/cytoplasmic fractions of HAECs upon GR knockdown and dexamethasone treatment. *n* = 4 per group. Data are presented as mean ± SD. Brown-Forsythe and Welch ANOVA, and unpaired t with Welch’s correction. Dex, dexamethasone; GR, glucocorticoid receptor; HAEC, human aortic endothelial cell; siScr, scrambled siRNA.

**
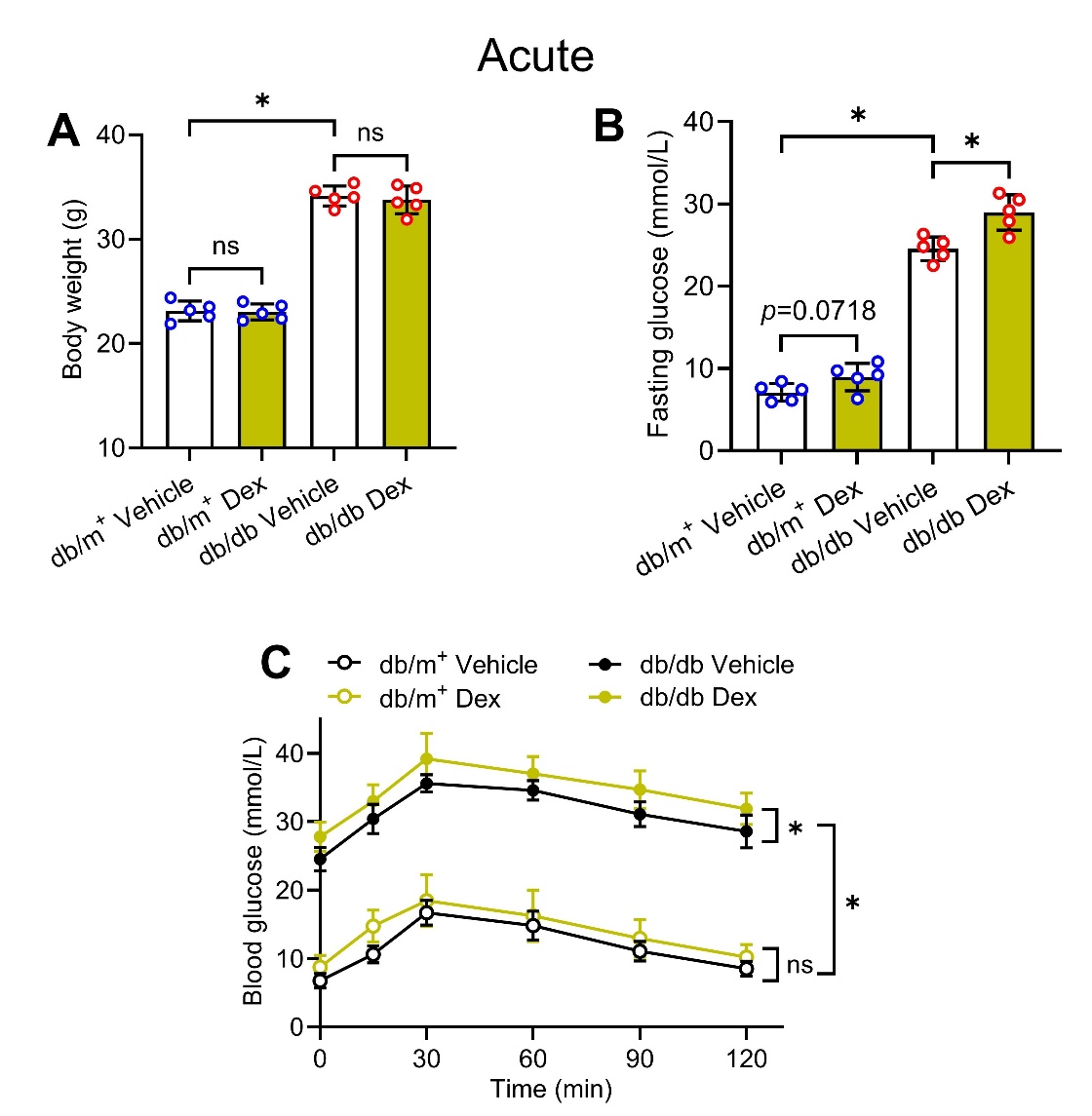
**

**Figure S45.** Effects of acute dexamethasone treatment on body weights and blood glucose levels of non-diabetic and diabetic mice. (**A**)**.** Body weights, (**B**) fasting glucose levels, and (**C**) OGTT of db/m^+^ and db/db mice 24 hr after dexamethasone injection (*n* = 5 per group). Data are presented as mean ± SD. **p* < 0.05 (Brown-Forsythe and Welch ANOVA, and unpaired t with Welch’s correction). Dex, dexamethasone; OGTT, oral glucose tolerance test.

**
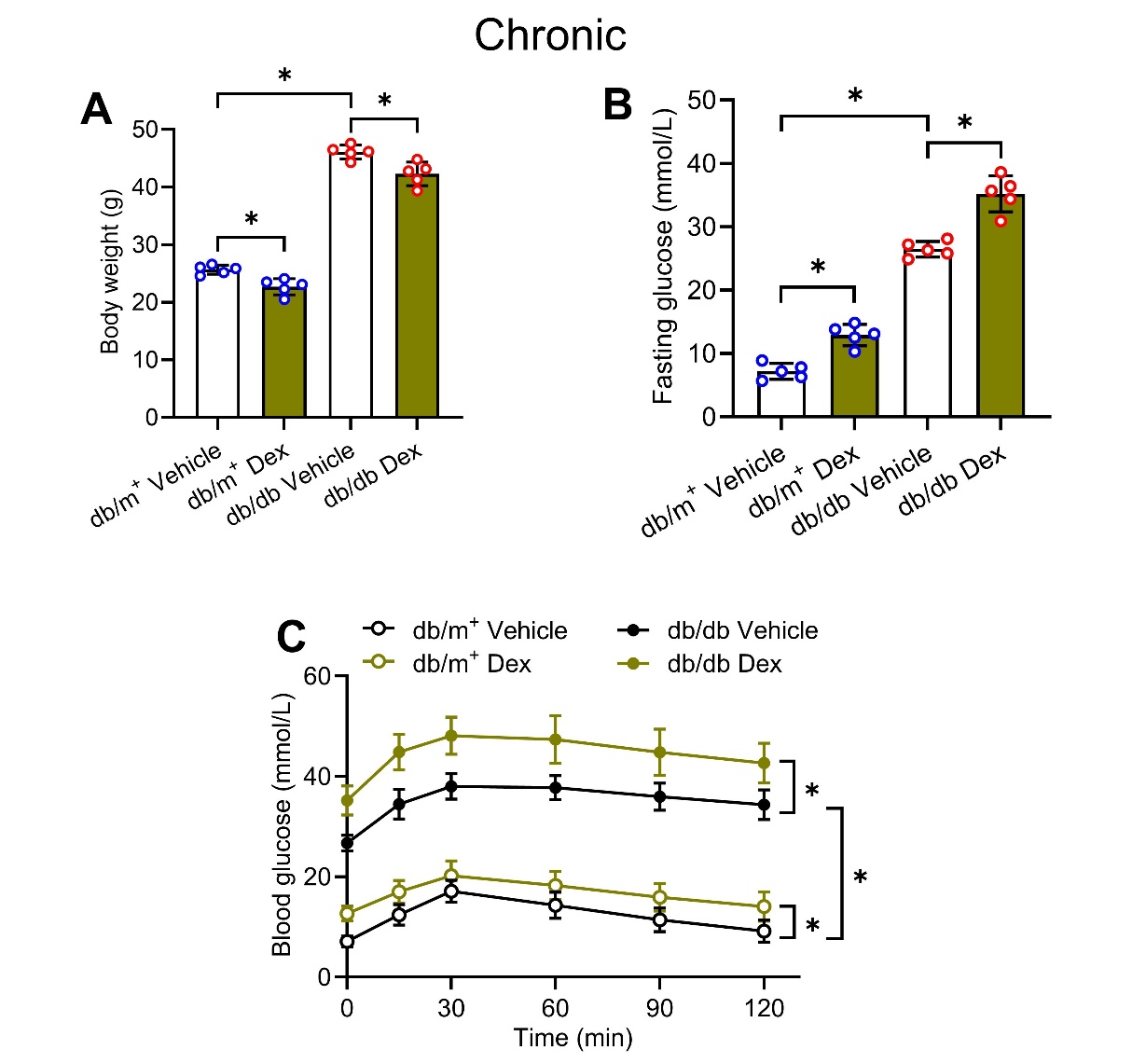
**

**Figure S46.** Effects of chronic dexamethasone treatment on body weights and blood glucose levels of non-diabetic and diabetic mice. (**A**) Body weights, (**B**) fasting glucose levels, and (**C**) OGTT of db/m^+^ and db/db mice after 4-week dexamethasone treatment (*n* = 5 per group). Data are presented as mean ± SD. **p* < 0.05 (Brown-Forsythe and Welch ANOVA, and unpaired t with Welch’s correction). Dex, dexamethasone; OGTT, oral glucose tolerance test.


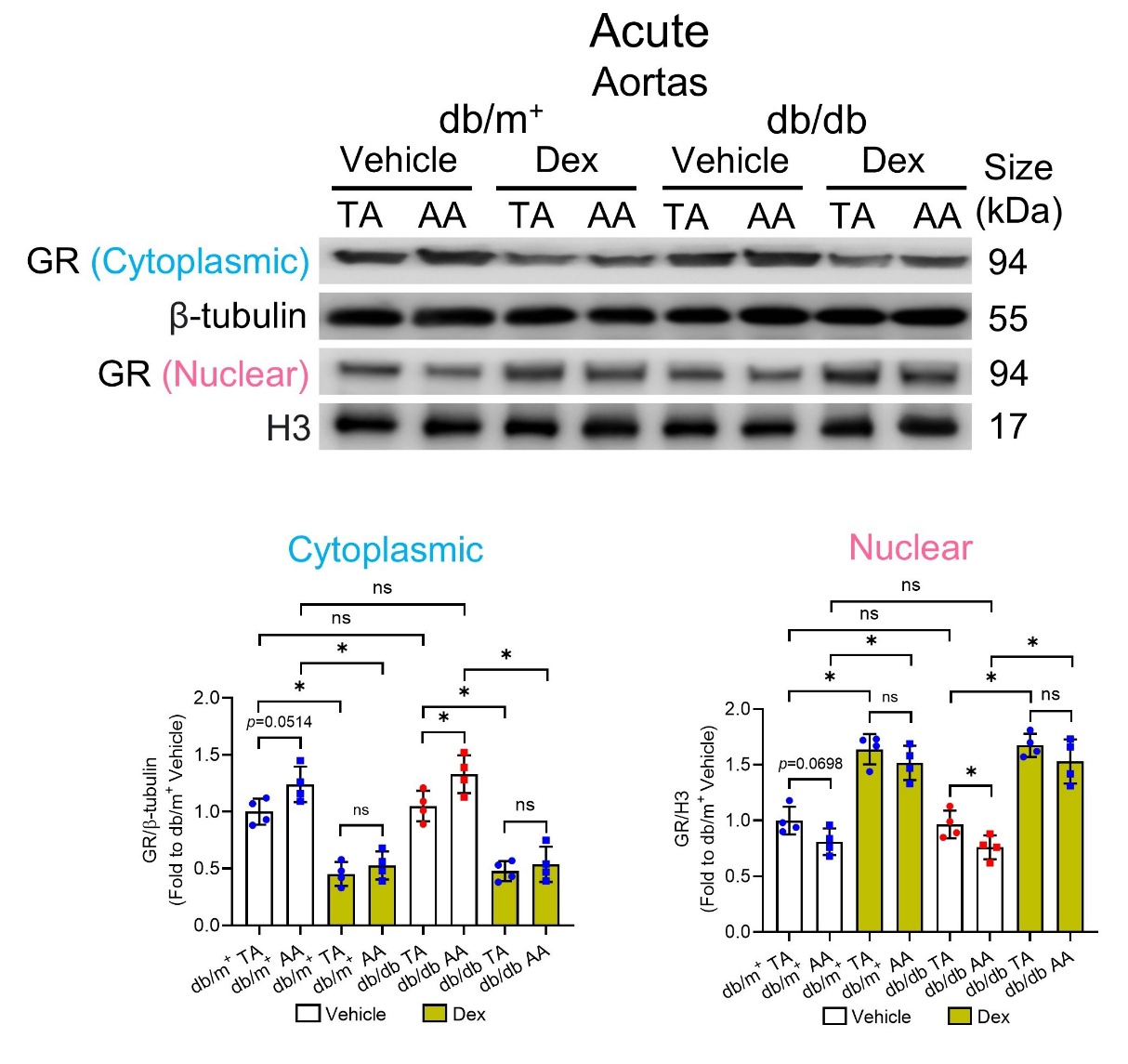


**Figure S47.** Western blotting on GR expression in nuclear/cytoplasmic fractions of non-diabetic and diabetic mouse aortas upon acute dexamethasone treatment. *n* = 4 per group. Data are presented as mean ± SD. Brown-Forsythe and Welch ANOVA, and unpaired t with Welch’s correction. AA, abdominal aorta; Dex, dexamethasone; GR, glucocorticoid receptor; TA, descending thoracic aorta.


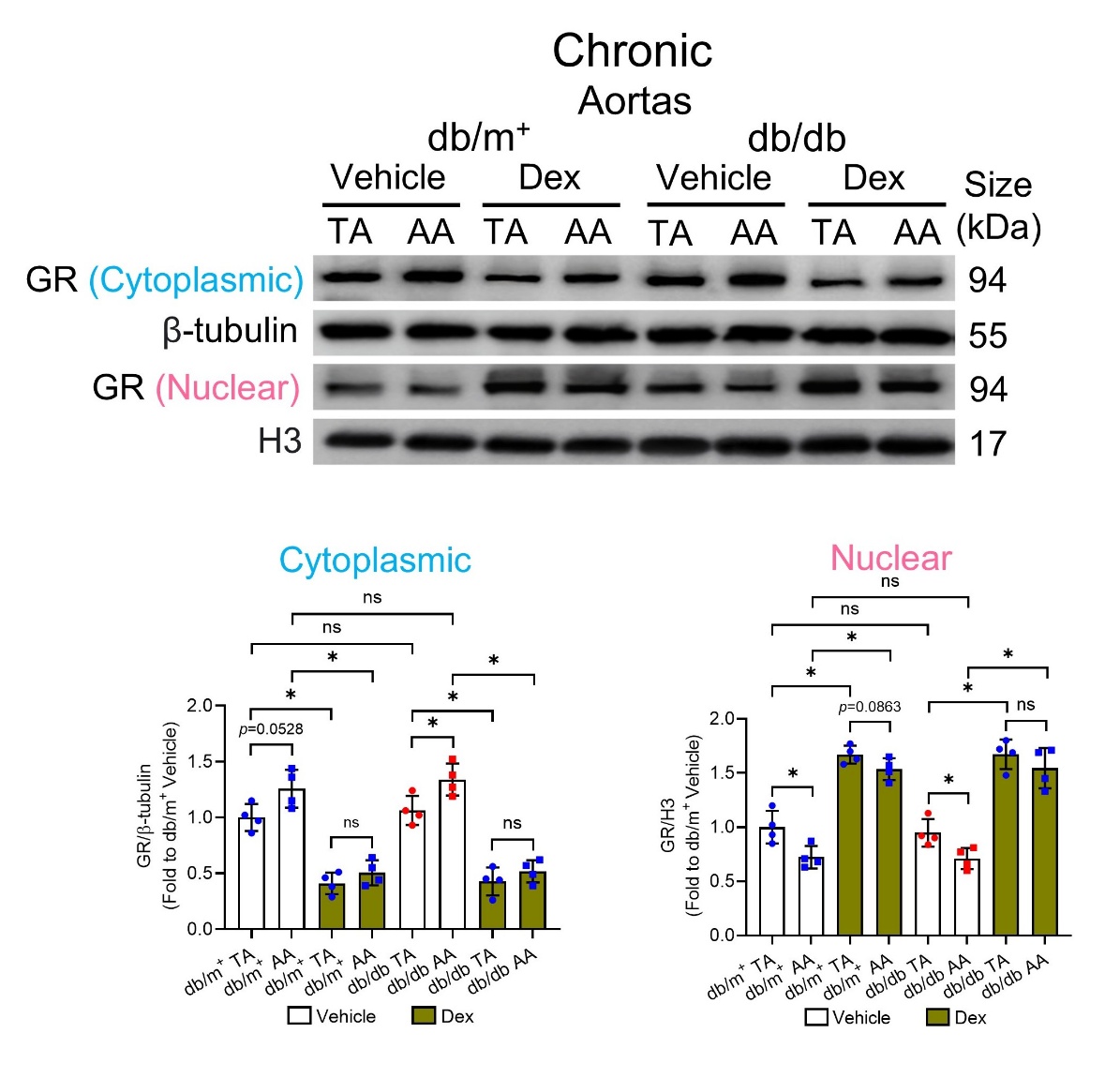


**Figure S48.** Western blotting on GR expression in nuclear/cytoplasmic fractions of non-diabetic and diabetic mouse aortas upon chronic dexamethasone treatment. *n* = 4 per group. Data are presented as mean ± SD. Brown-Forsythe and Welch ANOVA, and unpaired t with Welch’s correction. AA, abdominal aorta; Dex, dexamethasone; GR, glucocorticoid receptor; TA, descending thoracic aorta.


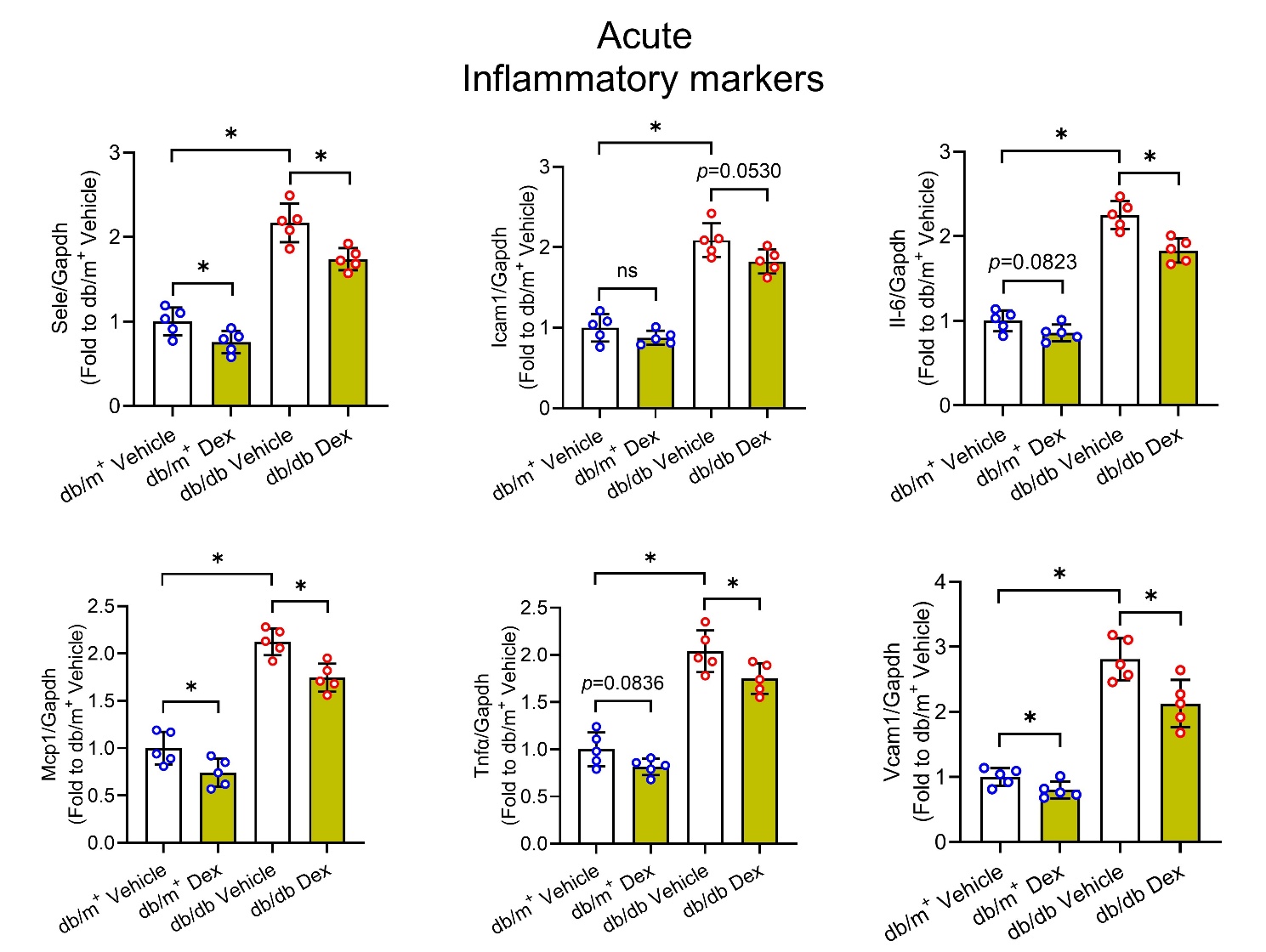


**Figure S49.** RT-PCR on inflammatory markers of mouse aortas upon acute dexamethasone treatment. *n* = 5 per group. Data are presented as mean ± SD. **p* < 0.05 (Brown-Forsythe and Welch ANOVA, and unpaired t with Welch’s correction). Dex, dexamethasone.


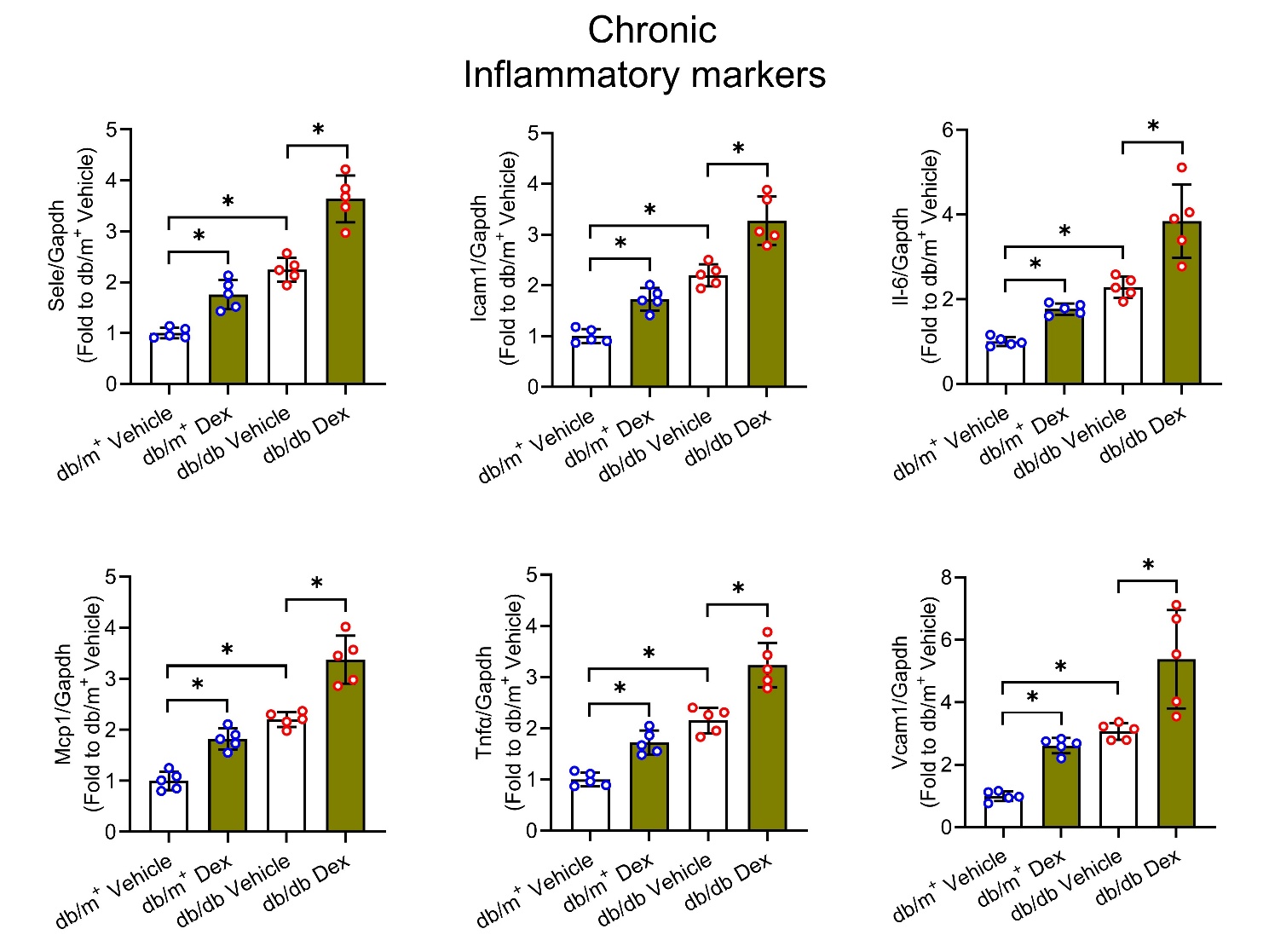


**Figure S50.** RT-PCR on inflammatory markers of mouse aortas upon chronic dexamethasone treatment. *n* = 5 per group. Data are presented as mean ± SD. **p* < 0.05 (Brown-Forsythe and Welch ANOVA, and unpaired t with Welch’s correction). Dex, dexamethasone.


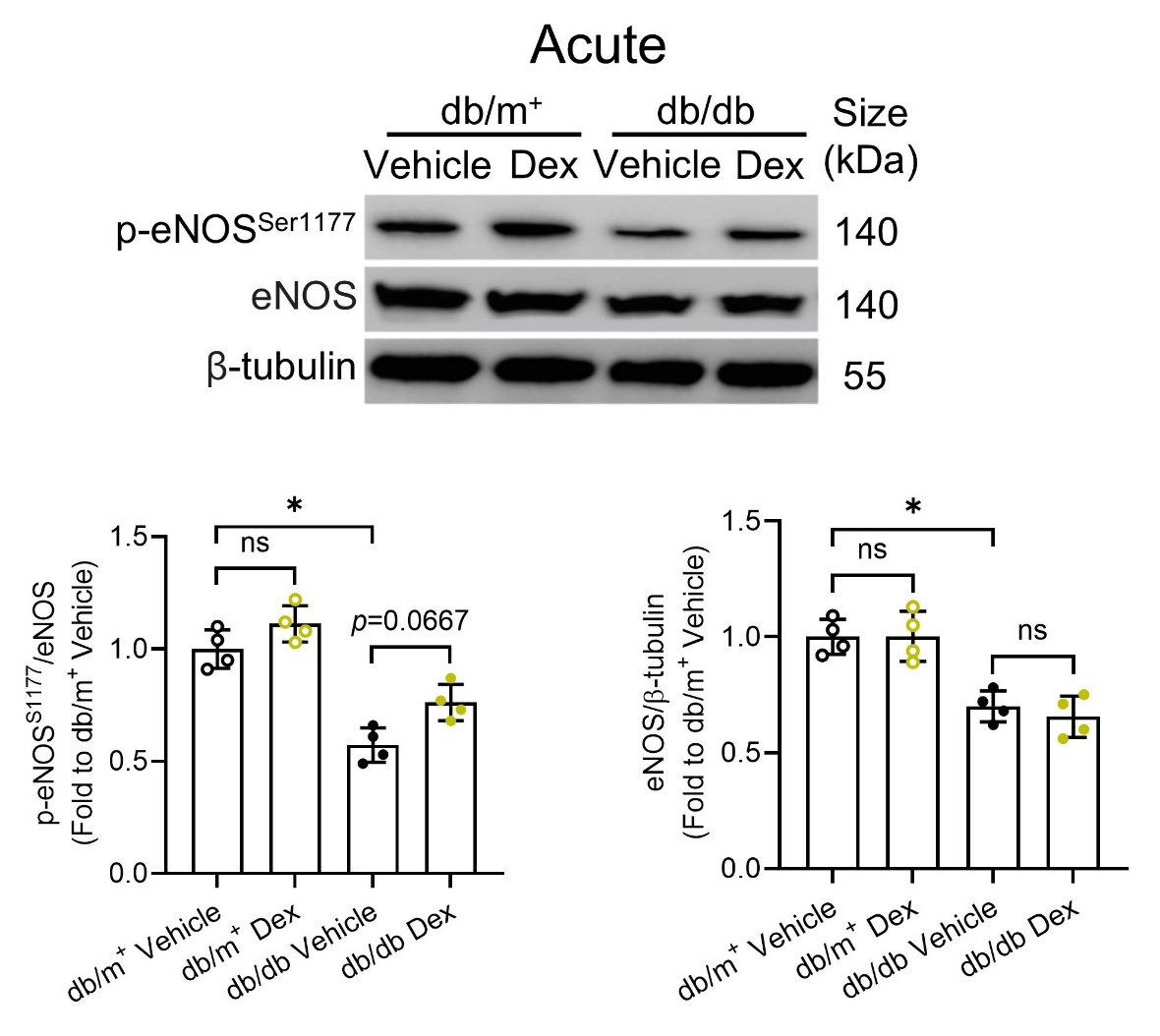


**Figure S51.** Western blotting on phosphorylated eNOS and total eNOS in aortas of non-diabetic and diabetic mice after acute dexamethasone treatment. *n* = 4 per group. Data are presented as mean ± SD. Brown-Forsythe and Welch ANOVA, and unpaired t with Welch’s correction. Dex, dexamethasone; eNOS, endothelial nitric oxide synthase.


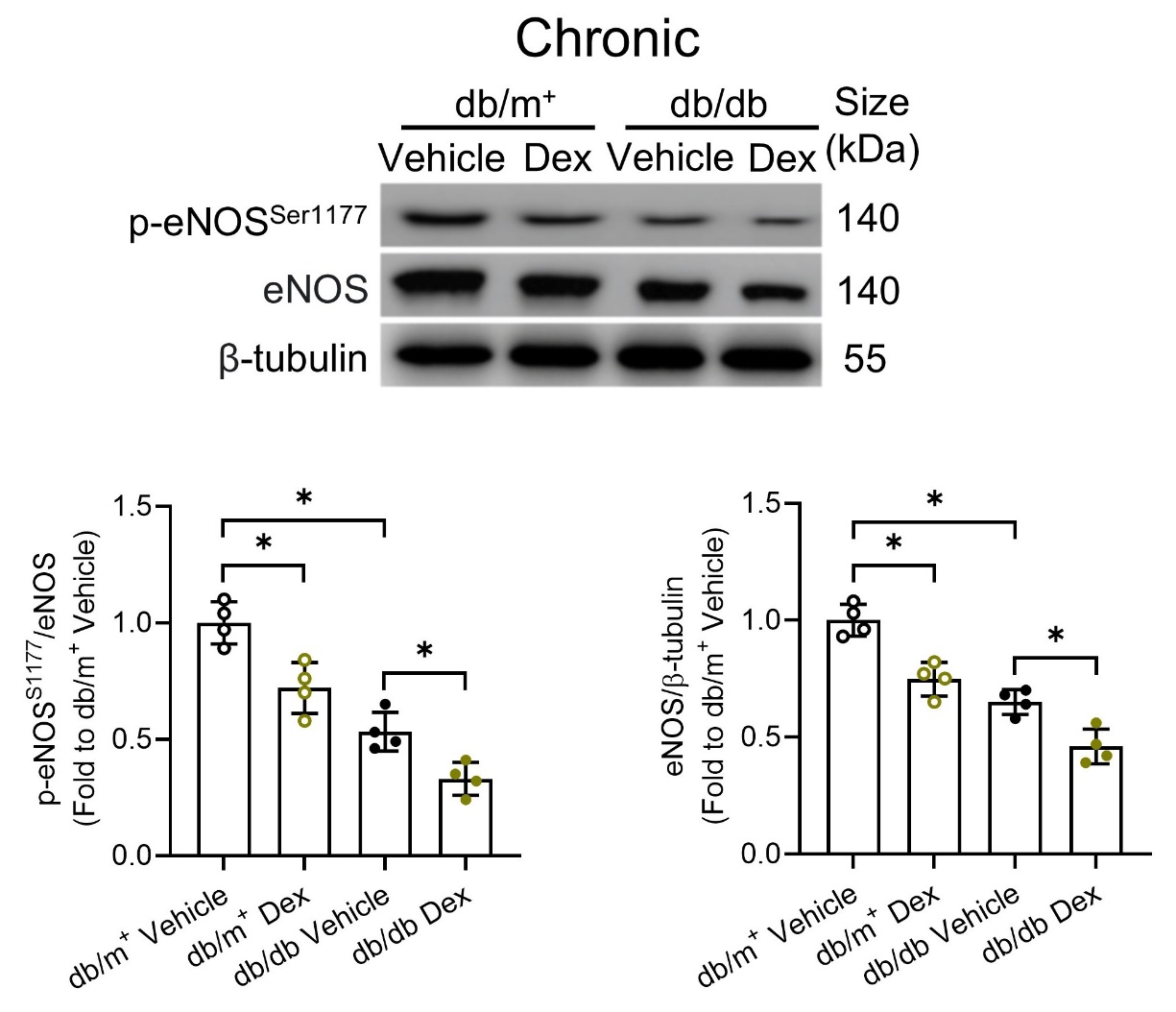


**Figure S52.** Western blotting on phosphorylated eNOS and total eNOS in aortas of non-diabetic and diabetic mice after chronic dexamethasone treatment. *n* = 4 per group. Data are presented as mean ± SD. Brown-Forsythe and Welch ANOVA, and unpaired t with Welch’s correction. Dex, dexamethasone; eNOS, endothelial nitric oxide synthase.

**
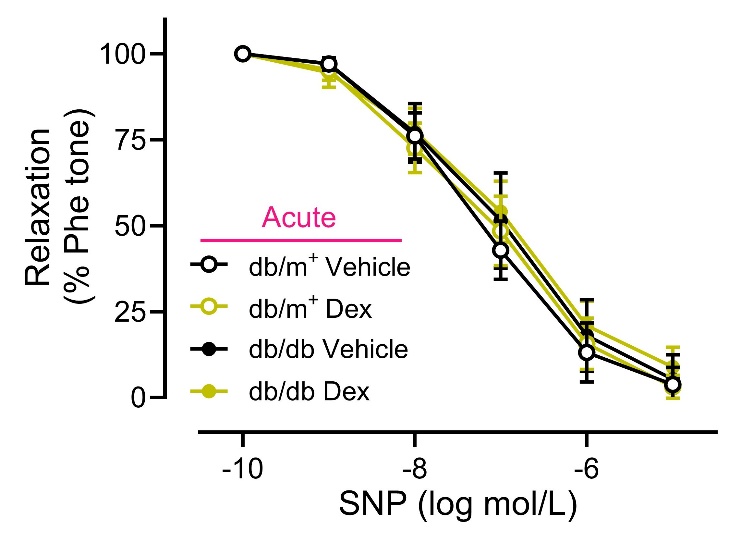
**

**Figure S53.** Endothelium-independent relaxations of diabetic mouse aortas upon acute dexamethasone treatment. *n* = 5 per group. Data are presented as mean ± SD. Brown-Forsythe and Welch ANOVA, and unpaired t with Welch’s correction. Dex dexamethasone; Phe, phenylephrine; SNP, sodium nitroprusside.

**
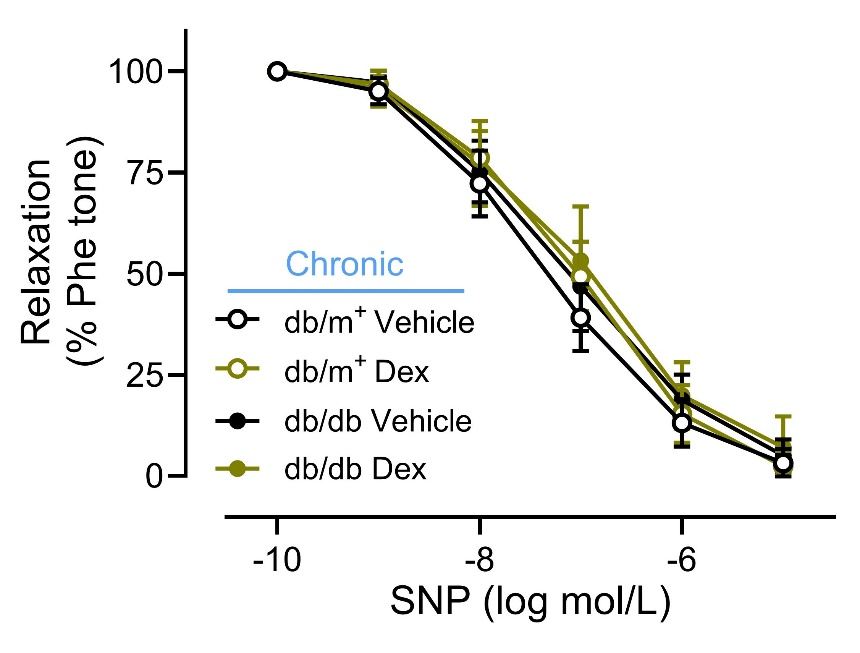
**

**Figure S54.** Endothelium-independent relaxations of diabetic mouse aortas upon chronic dexamethasone treatment. *n* = 5 per group. Data are presented as mean ± SD. Brown-Forsythe and Welch ANOVA, and unpaired t with Welch’s correction. Dex dexamethasone; Phe, phenylephrine; SNP, sodium nitroprusside.

**
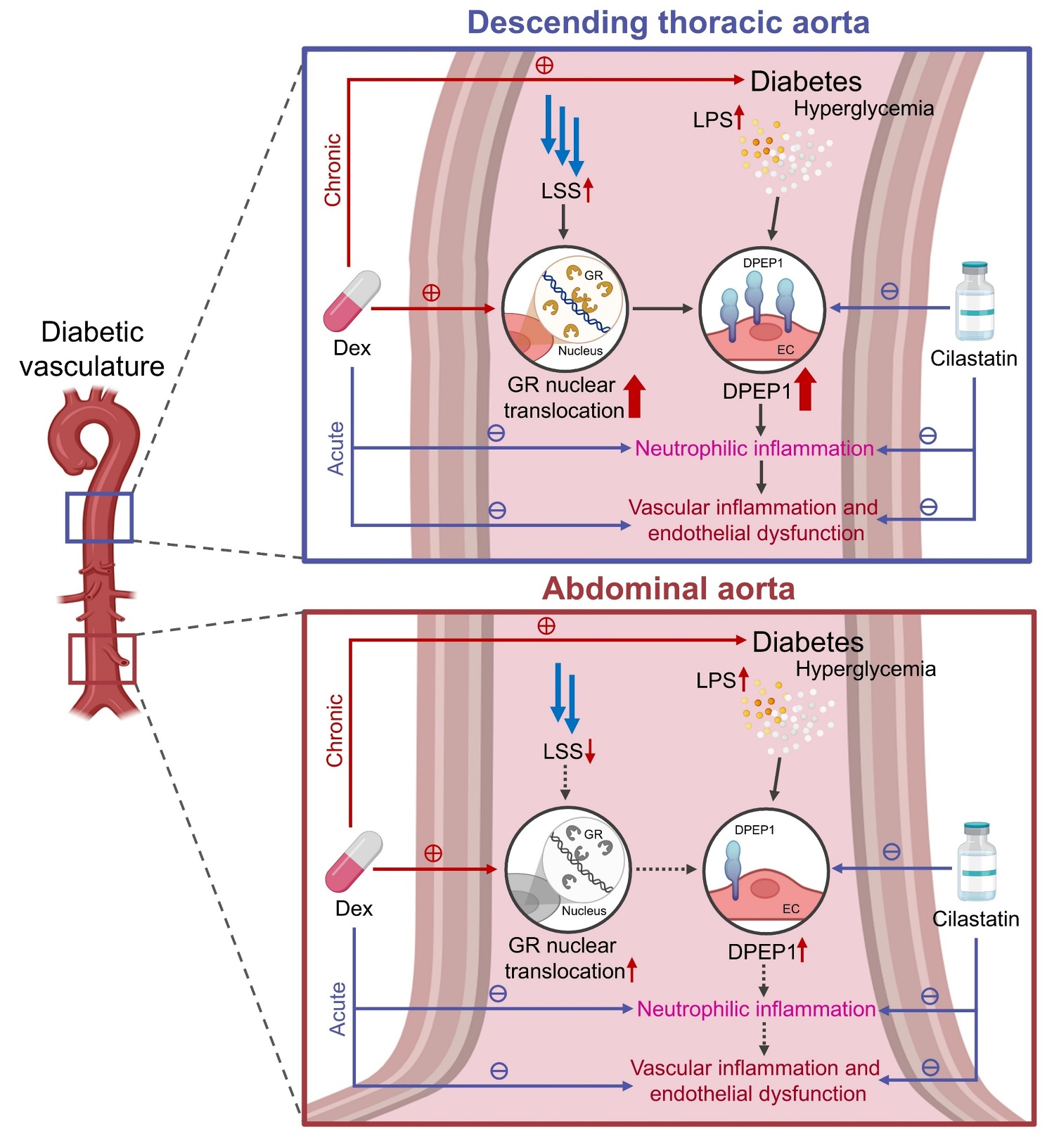
**

**Figure S55.** Spatially differential regulation of the GR/DPEP1 axis by hemodynamic shear stress and diabetic conditions. In diabetic TA, higher LSS magnitude and metabolic stressors co-activate the GR/DPEP1 axis in mediating neutrophilic inflammation and diabetic vasculopathy. In diabetic AA, lower LSS magnitude corresponds to less pronounced GR nuclear translocation and DPEP1 upregulation. Cilastatin treatment ameliorates the features of diabetic vasculopathy through suppressing DPEP1 activity. Though inducing GR nuclear translocation, dexamethasone exerts pleiotropic and time-dependent effects on diabetic vasculature, being acutely suppressive but chronically promotive to diabetic vasculopathy. AA, abdominal aorta; Dex, dexamethasone; DPEP1, dipeptidase 1; EC, endothelial cell; GR, glucocorticoid receptor; LPS, lipopolysaccharide; LSS, laminar shear stress; TA, descending thoracic aorta.

**PART III: Supplementary Tables**

**Table S1.** List of upregulated and downregulated DEPs in db/db aorta vs db/m^+^ aorta.

*Upregulated DEPs*

| **Gene name** | **db/db aorta/db/m^+^ aorta Ratio** | ***p* value** | **Regulated Type** |
| --- | --- | --- | --- |
| Mcpt2 | 76.61431503 | 0.010177641 | Up |
| Samd9l | 66.57075604 | 0.03032593 | Up |
| Napepld | 22.46785182 | 0.031141119 | Up |
| Prrc1 | 22.43616623 | 0.030617087 | Up |
| Sult1a1 | 14.42467041 | 0.030729066 | Up |
| Bpifa2 | 9.996463828 | 5.33795E-06 | Up |
| Prxl2b | 9.580121293 | 0.042633242 | Up |
| Timp2 | 8.263208493 | 0.010500855 | Up |
| Ndufb8 | 7.749284738 | 0.012937456 | Up |
| Cyp1b1 | 6.895357477 | 0.003429919 | Up |
| Dnajc19 | 6.363153004 | 0.014402809 | Up |
| Pld3 | 5.88347938 | 0.016091057 | Up |
| Tbrg4 | 5.763748001 | 0.032556117 | Up |
| Coq5 | 4.871181725 | 0.021366178 | Up |
| Pex19 | 4.645048345 | 0.048597356 | Up |
| Pts | 4.597218155 | 0.000606498 | Up |
| Ppp3r1 | 4.50026626 | 0.010921347 | Up |
| Trappc6b | 4.369570209 | 0.000712579 | Up |
| Map2 | 4.3572123 | 0.001010314 | Up |
| Crlf3 | 4.320865378 | 0.000690061 | Up |
| Tdrd3 | 4.284273947 | 0.009771596 | Up |
| Dhcr24 | 4.23009102 | 0.000724298 | Up |
| Elac2 | 4.098517678 | 0.013357252 | Up |
| Serinc1 | 3.975941745 | 0.00944211 | Up |
| Alg3 | 3.907290342 | 0.034818216 | Up |
| Necap1 | 3.586875997 | 0.013337775 | Up |
| Atg16l1 | 3.250889855 | 0.000867114 | Up |
| Fkbp2 | 3.015734237 | 0.046344834 | Up |
| Srrm4 | 2.749328883 | 0.033838787 | Up |
| Epdr1 | 2.658043252 | 0.022872389 | Up |
| Cnmd | 2.561555382 | 0.043720087 | Up |
| Xpo5 | 2.520786451 | 0.049064753 | Up |
| Rab9a | 2.503139326 | 0.02203208 | Up |
| Cyp2b10 | 2.502115896 | 0.034445643 | Up |
| Alpl | 2.489415636 | 0.031813259 | Up |
| S100a4 | 2.462946769 | 1.22515E-06 | Up |
| Iah1 | 2.362299777 | 0.023439616 | Up |
| Sfxn2 | 2.344476422 | 0.041432841 | Up |
| Cops7b | 2.340989297 | 0.048063703 | Up |
| Pm20d2 | 2.301242619 | 0.039879916 | Up |
| Hmgcs2 | 2.268487358 | 2.51023E-07 | Up |
| Fabp3 | 2.194992693 | 1.44061E-05 | Up |
| Slitrk4 | 2.190661106 | 8.80549E-05 | Up |
| Irgq | 2.18109975 | 0.02441494 | Up |
| Tubb4a | 2.120162851 | 0.030804238 | Up |
| Cbx5 | 2.081132247 | 0.013442169 | Up |
| Akt3 | 2.075794045 | 0.031174855 | Up |
| Serpine1 | 2.061412308 | 4.12506E-05 | Up |
| Slc38a2 | 2.01621164 | 0.042763714 | Up |
| Crispld2 | 2.00245173 | 0.033449711 | Up |
| S100a6 | 1.890134196 | 9.86054E-06 | Up |
| Atp6ap2 | 1.852446176 | 0.036854726 | Up |
| Tpm1 | 1.838078735 | 0.000764464 | Up |
| Dnttip2 | 1.826646685 | 0.001039071 | Up |
| Tpm2 | 1.800381148 | 0.000441901 | Up |
| Wdr13 | 1.737584052 | 0.043488331 | Up |
| Ccn5 | 1.719803986 | 0.000100914 | Up |
| Piezo1 | 1.706370175 | 0.047350616 | Up |
| Sema3c | 1.675955928 | 0.00121263 | Up |
| Atp5pf | 1.662175445 | 0.015661084 | Up |
| Dpep1 | 1.621273868 | 0.020338361 | Up |
| S100a13 | 1.592786447 | 0.004714722 | Up |
| Mfap2 | 1.59181156 | 0.003075729 | Up |
| Tnc | 1.580512052 | 3.48465E-05 | Up |
| Gc | 1.57542949 | 0.048385713 | Up |
| Sod3 | 1.564236341 | 6.92059E-05 | Up |
| Specc1 | 1.562937837 | 0.000340968 | Up |
| Apoa4 | 1.555325887 | 0.038330838 | Up |
| Postn | 1.553951199 | 4.60071E-05 | Up |
| Sparcl1 | 1.548733479 | 0.020890174 | Up |
| Sncg | 1.536602418 | 0.005929762 | Up |
| Sh3bgrl | 1.528944319 | 0.000502382 | Up |
| Sh3bgrl3 | 1.512891431 | 0.005178611 | Up |
| Rplp2 | 1.506484077 | 0.000955506 | Up |
| S100a11 | 1.505923403 | 0.001182588 | Up |
| Phgdh | 1.50229751 | 1.872E-05 | Up |
| Ror1 | 1.500449295 | 0.001956543 | Up |

*Downregulated DEPs*

| **Gene name** | **db/db aorta/db/m+ aorta Ratio** | ***p* value** | **Regulated Type** |
| --- | --- | --- | --- |
| Krt13 | 0.00456581 | 0.037573703 | Down |
| Serpina1e | 0.009789958 | 1.31484E-06 | Down |
| Casp14 | 0.009820968 | 0.034069519 | Down |
| Krt75 | 0.01349052 | 0.011741958 | Down |
| Ctsf | 0.021699613 | 0.03316231 | Down |
| Scd1 | 0.022302684 | 0.003199183 | Down |
| Qrsl1 | 0.027931361 | 0.011674927 | Down |
| Szt2 | 0.028953545 | 0.03319166 | Down |
| Rfc4 | 0.040938489 | 0.033268242 | Down |
| Aspg | 0.05059871 | 0.012236906 | Down |
| Fbxo2 | 0.051494195 | 0.033715896 | Down |
| Mcrip2 | 0.051888902 | 0.038375127 | Down |
| B3gat3 | 0.055031565 | 0.033619829 | Down |
| Mup18 | 0.059638138 | 3.14651E-06 | Down |
| Mrps23 | 0.064014959 | 0.033374411 | Down |
| Eno2 | 0.064602624 | 0.040393734 | Down |
| Myo5a | 0.066614393 | 0.001992199 | Down |
| Iigp1 | 0.066833725 | 0.011966276 | Down |
| Akt1s1 | 0.083986005 | 0.036532604 | Down |
| Fasn | 0.087609538 | 1.70441E-06 | Down |
| Gvin1 | 0.098934531 | 0.010576952 | Down |
| Aloxe3 | 0.115262709 | 0.041971552 | Down |
| Sdhd | 0.117422419 | 0.011734646 | Down |
| Akap8l | 0.120282747 | 0.034659376 | Down |
| Wdsub1 | 0.126162429 | 0.015606261 | Down |
| Mamdc2 | 0.126606907 | 0.01171781 | Down |
| Washc1 | 0.132457699 | 0.037004068 | Down |
| Aga | 0.136980735 | 0.048740688 | Down |
| C8b | 0.167784728 | 0.000284662 | Down |
| Ctsk | 0.174703639 | 0.02640366 | Down |
| Thrsp | 0.174936556 | 0.001506393 | Down |
| C8a | 0.180381502 | 0.007996245 | Down |
| Coro1a | 0.186526728 | 0.018114414 | Down |
| Ifi204 | 0.201382844 | 0.015261429 | Down |
| C1qc | 0.238268532 | 0.002939317 | Down |
| Saa2 | 0.252883599 | 0.009496132 | Down |
| Fam210b | 0.260806625 | 0.044010498 | Down |
| C1qb | 0.263563584 | 0.020475797 | Down |
| Poldip3 | 0.272285057 | 0.042160331 | Down |
| Acaca | 0.326989278 | 0.0001477 | Down |
| Aldh1a7 | 0.333290359 | 0.006901821 | Down |
| Acly | 0.334082283 | 3.36887E-06 | Down |
| Gpd1 | 0.339971231 | 5.40689E-06 | Down |
| Scara5 | 0.346770373 | 0.04188648 | Down |
| C4bpa | 0.360183619 | 0.043062384 | Down |
| Ppp1r14c | 0.380445283 | 0.039518199 | Down |
| Pcyt2 | 0.39432044 | 0.023431619 | Down |
| Bcas2 | 0.418205041 | 0.009669753 | Down |
| Coro7 | 0.426630368 | 0.028813167 | Down |
| Acacb | 0.449265402 | 0.024161831 | Down |
| Baz1b | 0.449680673 | 0.02739897 | Down |
| Mbl1 | 0.450150183 | 0.000300904 | Down |
| Hp | 0.451765138 | 0.046908325 | Down |
| Hmga1 | 0.458300712 | 0.023550842 | Down |
| Pc | 0.461517043 | 0.011828735 | Down |
| Man2b2 | 0.465578678 | 0.012935174 | Down |
| Saa4 | 0.472529407 | 0.004478288 | Down |
| Me1 | 0.478039871 | 2.5511E-05 | Down |
| Aacs | 0.483551322 | 0.000171892 | Down |
| Saa1 | 0.503238066 | 0.010624008 | Down |
| Mgst1 | 0.503962358 | 0.047663576 | Down |
| Arl6ip1 | 0.504902167 | 0.042485516 | Down |
| Acsl5 | 0.508040951 | 0.000523678 | Down |
| Srcin1 | 0.510340723 | 0.043644057 | Down |
| Nrp1 | 0.520500965 | 0.043109504 | Down |
| Fgg | 0.521078882 | 0.000248145 | Down |
| Tst | 0.521816366 | 3.98584E-05 | Down |
| Fgb | 0.544633021 | 0.000560401 | Down |
| Acss2 | 0.547871201 | 0.000546721 | Down |
| Pfn1 | 0.555697034 | 0.010490128 | Down |
| Fga | 0.55846386 | 0.000937024 | Down |
| Mustn1 | 0.564699651 | 0.036183445 | Down |
| Cd163 | 0.56792753 | 0.019492413 | Down |
| Fam98a | 0.569021528 | 0.020183287 | Down |
| Echdc1 | 0.571297737 | 0.0011879 | Down |
| Tkt | 0.584879934 | 0.000116973 | Down |
| Nes | 0.587929503 | 0.002896733 | Down |
| Zbtb20 | 0.588057395 | 0.002100808 | Down |
| Pgd | 0.595953566 | 0.000129122 | Down |
| Aldh4a1 | 0.597329597 | 0.000678032 | Down |
| Hk2 | 0.599503947 | 0.000616884 | Down |
| Scyl2 | 0.606520176 | 0.025048832 | Down |
| Prxl2a | 0.611235865 | 0.014977998 | Down |
| Ntn1 | 0.625143149 | 0.000266743 | Down |
| Pygl | 0.630218636 | 0.002852135 | Down |
| Rpl35 | 0.631918401 | 0.001277841 | Down |
| Vtn | 0.632950536 | 0.000346848 | Down |
| Hmgb1 | 0.633435783 | 0.024025047 | Down |
| Rhoc | 0.63668028 | 0.026865482 | Down |
| Gpd2 | 0.636748947 | 0.03681678 | Down |
| Nup85 | 0.637859898 | 0.001358004 | Down |
| Phospho1 | 0.64016014 | 0.001081033 | Down |
| Crocc | 0.64327091 | 0.00282014 | Down |
| C5 | 0.644560053 | 0.037752447 | Down |
| H2-L | 0.65025793 | 5.76488E-05 | Down |
| Mif | 0.658015839 | 0.016491149 | Down |
| Prpsap1 | 0.665280009 | 0.045083805 | Down |

**Table S2.** List of upregulated and downregulated DEPs in db/m^+^ AA vs db/m^+^ TA.

*Upregulated DEPs*

| **Gene name** | **db/m^+^ AA/db/m^+^ TA Ratio** | ***p* value** | **Regulated Type** |
| --- | --- | --- | --- |
| Hook2 | 3570.766022 | 1.81913E-09 | Up |
| Tuba3a | 988.3906558 | 1.2336E-13 | Up |
| Cand2 | 389.9315574 | 1.97666E-09 | Up |
| Ppil4 | 175.2988245 | 0.024190978 | Up |
| Cox6a1 | 174.1012338 | 8.23932E-06 | Up |
| Crispld2 | 171.9113542 | 1.03412E-10 | Up |
| Ca5b | 142.5285052 | 1.47743E-05 | Up |
| Fkbp2 | 126.3967753 | 0.024556182 | Up |
| Ift172 | 101.2991544 | 7.00459E-05 | Up |
| Hint3 | 100.7485236 | 0.024244916 | Up |
| Ctsf | 91.16754394 | 5.74786E-12 | Up |
| Mrps7 | 90.1974194 | 0.024115837 | Up |
| Tgfbrap1 | 73.89645978 | 8.622E-10 | Up |
| Eipr1 | 73.77321862 | 7.75301E-07 | Up |
| Surf4 | 73.30031388 | 4.73295E-05 | Up |
| Map3k11 | 71.17764746 | 3.31487E-10 | Up |
| Szt2 | 68.07617009 | 1.18837E-10 | Up |
| Mrpl11 | 67.08073992 | 0.025864596 | Up |
| Ptges2 | 67.00202085 | 5.95079E-06 | Up |
| Plbd1 | 66.54461477 | 0.025209292 | Up |
| Hbb-bh1 | 64.66437985 | 4.59284E-06 | Up |
| Ndufa11 | 63.24728534 | 5.91211E-06 | Up |
| Polr2c | 61.25303456 | 0.025384737 | Up |
| Wdfy4 | 57.67697284 | 0.026447627 | Up |
| Mrpl38 | 56.79680486 | 0.024218188 | Up |
| Slc35b1 | 56.07436074 | 0.024233422 | Up |
| Atpaf1 | 55.97348465 | 0.024270315 | Up |
| Rab9a | 54.4675782 | 1.60991E-08 | Up |
| Tubb4a | 52.87578157 | 5.52131E-10 | Up |
| Asns | 51.67351958 | 0.025478356 | Up |
| Fbxl4 | 49.81345938 | 0.026677495 | Up |
| Zfand6 | 47.64078873 | 0.024103845 | Up |
| Atp6ap2 | 46.40299253 | 2.70642E-09 | Up |
| Prim2 | 43.17007715 | 0.026797689 | Up |
| Mtatp6 | 42.32749083 | 4.87254E-05 | Up |
| Gtf3c1 | 42.18285063 | 1.1216E-07 | Up |
| Fbxo2 | 37.83932922 | 0.00012616 | Up |
| Ptpmt1 | 37.78395246 | 0.024366962 | Up |
| Akt3 | 37.66553002 | 6.6635E-10 | Up |
| Mipep | 35.71206632 | 0.024405142 | Up |
| Arhgap18 | 35.29672219 | 0.024373545 | Up |
| Cbx5 | 33.69861943 | 0.024588989 | Up |
| Nfkb2 | 31.87555448 | 1.83224E-05 | Up |
| Abcb6 | 31.53018284 | 2.20072E-09 | Up |
| Ppp2r5b | 31.49489735 | 0.024860257 | Up |
| Wdr37 | 31.13689358 | 9.7561E-07 | Up |
| Rimoc1 | 31.03570678 | 4.85239E-08 | Up |
| Ubqln1 | 30.06364018 | 1.50621E-08 | Up |
| Arhgap5 | 29.12431318 | 0.025421192 | Up |
| Gask1b | 26.60470946 | 1.41818E-08 | Up |
| Wdr43 | 26.16177539 | 1.96095E-05 | Up |
| Gtf2f2 | 25.68785182 | 0.025247094 | Up |
| Oxr1 | 23.75048409 | 0.02500549 | Up |
| Pex11b | 22.25405812 | 0.026049073 | Up |
| Dym | 20.70525462 | 0.024425964 | Up |
| Tbc1d22a | 20.6994648 | 0.024855182 | Up |
| Thoc2 | 20.31888451 | 9.75992E-05 | Up |
| Stx2 | 19.7273602 | 0.025056145 | Up |
| Smyd5 | 18.316609 | 0.024109364 | Up |
| Parp4 | 18.01717797 | 0.024337328 | Up |
| Mta1 | 14.86766192 | 0.024366224 | Up |
| Phkb | 14.84642645 | 5.45661E-06 | Up |
| Ccdc102a | 14.7142066 | 1.68047E-10 | Up |
| Man2a2 | 13.93355907 | 0.001164886 | Up |
| Arid1b | 13.83779222 | 0.024937504 | Up |
| Carnmt1 | 13.76845251 | 0.028702449 | Up |
| Plod2 | 13.50119747 | 2.09889E-06 | Up |
| Atg4b | 13.07734203 | 0.000487686 | Up |
| Prpf38b | 12.11273244 | 0.024036238 | Up |
| Pelo | 11.37683943 | 0.002044767 | Up |
| Saal1 | 11.2966384 | 0.000196958 | Up |
| Vps33b | 10.49489894 | 0.001079588 | Up |
| Znrd2 | 10.48394191 | 0.02429816 | Up |
| Trio | 9.571790749 | 0.028455273 | Up |
| Coq7 | 9.563876555 | 0.030907884 | Up |
| Stim2 | 9.527002867 | 0.024866368 | Up |
| Rrm1 | 8.820535841 | 0.003413153 | Up |
| Ccdc43 | 8.619082503 | 0.010690228 | Up |
| Stat5a | 8.466791385 | 0.024028149 | Up |
| Ddx39a | 8.063632138 | 0.026296599 | Up |
| Dnajc24 | 7.75142727 | 0.013456716 | Up |
| Fads2 | 7.274052916 | 0.025507567 | Up |
| Plbd2 | 7.117318787 | 0.010308436 | Up |
| Hmbs | 6.919509239 | 0.009727998 | Up |
| Tm9sf4 | 6.848599437 | 0.0137737 | Up |
| Msto1 | 6.825882208 | 1.11937E-08 | Up |
| Chmp7 | 6.771234229 | 2.66985E-05 | Up |
| Dap3 | 6.650579944 | 0.022516567 | Up |
| Dhx32 | 6.457834497 | 0.025545359 | Up |
| Bysl | 6.206299669 | 0.026784323 | Up |
| Pdcd11 | 6.033016263 | 0.00035668 | Up |
| Ppip5k1 | 5.486773738 | 0.000417425 | Up |
| Angel2 | 5.451445168 | 0.003440576 | Up |
| Dhcr7 | 5.211165474 | 0.015875063 | Up |
| Ap4s1 | 5.166486411 | 0.002936761 | Up |
| Acsl6 | 5.155981493 | 0.017878605 | Up |
| Bcl2l1 | 5.083493917 | 0.015666216 | Up |
| Caskin2 | 4.90871038 | 0.016806672 | Up |
| Exoc7 | 4.805579772 | 0.018071948 | Up |
| Alg1 | 4.782202096 | 0.016988718 | Up |
| Snrpg | 4.536861125 | 0.022994195 | Up |
| Prrt2 | 4.499557837 | 0.023137037 | Up |
| Itfg1 | 4.354760545 | 0.002245239 | Up |
| Krt7 | 4.327998082 | 0.02991178 | Up |
| Rbm28 | 4.282711019 | 0.019722709 | Up |
| Pgs1 | 4.122924108 | 0.022232206 | Up |
| Ncbp1 | 4.020095037 | 0.015463781 | Up |
| Rab13 | 3.996760323 | 0.026975103 | Up |
| Prr19 | 3.981354943 | 0.024280697 | Up |
| Nup188 | 3.929464587 | 0.00742121 | Up |
| Smad1 | 3.844364247 | 0.023226495 | Up |
| Krt10 | 3.829839371 | 0.014018245 | Up |
| Parl | 3.827371569 | 0.023977062 | Up |
| Rapgef2 | 3.789579063 | 0.020935314 | Up |
| Asah1 | 3.77615689 | 0.025286831 | Up |
| Ranbp9 | 3.732701529 | 0.032874938 | Up |
| Toe1 | 3.663623731 | 0.022705938 | Up |
| Srsf9 | 3.445868 | 0.027497578 | Up |
| Reep3 | 3.227388813 | 0.032741109 | Up |
| Krt77 | 3.213047881 | 0.046213591 | Up |
| Krt1 | 3.135933825 | 0.049360078 | Up |
| Ttc1 | 3.081400472 | 0.031674166 | Up |
| Mtr | 3.08117299 | 0.031669981 | Up |
| Krt76 | 3.069676542 | 0.0254071 | Up |
| Tap1 | 3.052665635 | 0.038343504 | Up |
| Lyz1 | 3.045064069 | 0.037941545 | Up |
| Trim25 | 2.968654278 | 0.033293811 | Up |
| Srcin1 | 2.963437855 | 0.000196237 | Up |
| Supt6h | 2.911792637 | 0.033542264 | Up |
| L1cam | 2.856290038 | 0.037644002 | Up |
| Krt2 | 2.832227355 | 0.018941977 | Up |
| Ppie | 2.714662549 | 0.041465158 | Up |
| Ndrg3 | 2.628909007 | 0.044484664 | Up |
| Metap1 | 2.624740777 | 0.042642189 | Up |
| Mpv17 | 2.479330139 | 0.041414651 | Up |
| Aga | 2.479301871 | 0.04007146 | Up |
| Rab8b | 2.342764106 | 0.043629297 | Up |
| Fcsk | 2.285746552 | 0.000548009 | Up |
| Gbp3 | 2.247429045 | 0.046945056 | Up |
| Gsdma | 2.134695667 | 0.033521786 | Up |
| Flad1 | 2.115541495 | 0.014112941 | Up |
| Sidt2 | 2.095070933 | 0.00078749 | Up |
| Nrdc | 2.086496149 | 0.002195849 | Up |
| Adgre5 | 2.054473701 | 0.031304431 | Up |
| Zc3hc1 | 1.944820777 | 0.015063134 | Up |
| Ppfibp1 | 1.893113032 | 0.023965799 | Up |
| Myo9b | 1.854338623 | 0.018369779 | Up |
| Ptprd | 1.790869635 | 0.007866278 | Up |
| Inf2 | 1.789963205 | 0.002893592 | Up |
| Pcsk5 | 1.780055773 | 0.002605621 | Up |
| Hmgn2 | 1.71279032 | 0.033387107 | Up |
| Acss1 | 1.645143856 | 0.049403192 | Up |
| Cox4i1 | 1.625670309 | 0.015081019 | Up |
| Zw10 | 1.623244044 | 0.032854254 | Up |
| Tspo | 1.621863436 | 0.016221798 | Up |
| Ccdc22 | 1.610492946 | 0.001980172 | Up |
| Skic3 | 1.602452004 | 0.013634243 | Up |
| Dpysl4 | 1.590695132 | 0.021046818 | Up |
| Ube2r2 | 1.588514464 | 0.031754801 | Up |
| Mfap4 | 1.588091855 | 0.034254787 | Up |
| Pcbd2 | 1.58190779 | 0.003636379 | Up |
| Abhd14b | 1.567455001 | 0.007447602 | Up |
| Fam162a | 1.558090567 | 0.026783459 | Up |
| Nrp1 | 1.551697511 | 0.010426052 | Up |
| Txnrd2 | 1.55056322 | 0.028955765 | Up |
| Copz1 | 1.547683371 | 0.003925627 | Up |
| Map2k2 | 1.543129352 | 0.001830957 | Up |
| Prkar2a | 1.540177316 | 0.002364357 | Up |
| Dock5 | 1.531625974 | 0.009186655 | Up |
| Klc2 | 1.528904935 | 0.040487179 | Up |
| Sar1a | 1.527875234 | 0.00093373 | Up |
| Atxn10 | 1.527100395 | 0.000448983 | Up |
| Il17b | 1.525662808 | 0.017161239 | Up |
| Rpl18a | 1.515333648 | 0.006383975 | Up |
| Sdr39u1 | 1.515252081 | 0.047287343 | Up |
| Wfdc1 | 1.514066185 | 0.000386404 | Up |
| Fbxo4 | 1.512626759 | 0.022201529 | Up |
| Smarcc2 | 1.508653273 | 0.001297093 | Up |
| Sap18 | 1.506013378 | 0.041110002 | Up |

*Downregulated DEPs*

| **Gene name** | **db/m^+^ AA/db/m^+^ TA Ratio** | ***p* value** | **Regulated Type** |
| --- | --- | --- | --- |
| Surf6 | 0.005340771 | 5.66866E-10 | Down |
| Elmo2 | 0.012066492 | 4.41778E-07 | Down |
| Vldlr | 0.013584084 | 0.024217605 | Down |
| Arsa | 0.01553951 | 0.024117348 | Down |
| Plekhf1 | 0.015667971 | 1.32461E-07 | Down |
| Mras | 0.015818361 | 2.31546E-08 | Down |
| Ube2v1 | 0.016582349 | 1.48588E-09 | Down |
| Isoc2b | 0.017020652 | 0.024461768 | Down |
| Arf2 | 0.019810594 | 0.000104049 | Down |
| Sfrp1 | 0.022593789 | 4.70428E-05 | Down |
| Idi1 | 0.025954494 | 0.025205259 | Down |
| Cuta | 0.028973403 | 0.024172158 | Down |
| S100a1 | 0.032089849 | 0.027467436 | Down |
| Bcl2 | 0.041959432 | 0.024401508 | Down |
| Sec61g | 0.042704028 | 7.2833E-07 | Down |
| Prss23 | 0.045659442 | 0.024793989 | Down |
| Itpr2 | 0.070169204 | 0.00561151 | Down |
| Washc1 | 0.070926211 | 0.002115983 | Down |
| Pls1 | 0.196001364 | 0.021602653 | Down |
| Wdr18 | 0.200423235 | 0.013703989 | Down |
| Fam210b | 0.200490978 | 0.030375264 | Down |
| Mrps35 | 0.201787939 | 0.024458597 | Down |
| Trmt112 | 0.236470217 | 0.022976891 | Down |
| Tbc1d17 | 0.242239793 | 0.022280639 | Down |
| Rabggtb | 0.249399553 | 0.023046377 | Down |
| Cpa3 | 0.274754066 | 0.030659935 | Down |
| Ppl | 0.27563577 | 0.000245855 | Down |
| Rilp | 0.284756511 | 0.002558924 | Down |
| Rhot2 | 0.294729027 | 0.03105479 | Down |
| Kcnh3 | 0.301131514 | 0.02823377 | Down |
| Mcpt3 | 0.327637085 | 0.033346412 | Down |
| Dennd4c | 0.421163893 | 0.019171187 | Down |
| Rida | 0.450223083 | 0.048134309 | Down |
| Coro7 | 0.477649812 | 0.033780276 | Down |
| Fsip2 | 0.543510183 | 0.037746386 | Down |
| Tmprss13 | 0.615842878 | 0.004712965 | Down |
| Pfas | 0.623241932 | 0.030944262 | Down |
| Mtpn | 0.632848061 | 0.020070562 | Down |
| Nudt2 | 0.65323029 | 0.040896951 | Down |
| Tmod1 | 0.653700037 | 2.17046E-05 | Down |
| Cmpk2 | 0.65919257 | 0.022683801 | Down |

**Table S3.** List of upregulated and downregulated DEPs in db/db AA vs db/db TA.

*Upregulated DEPs*

| **Gene name** | **db/db AA/db/db TA Ratio** | ***p* value** | **Regulated Type** |
| --- | --- | --- | --- |
| Eif2b4 | 130.5100694 | 3.15167E-07 | Up |
| Stx16 | 89.30812257 | 1.05293E-08 | Up |
| Tsfm | 60.80434994 | 3.36684E-06 | Up |
| Vkorc1l1 | 59.53263813 | 8.34987E-08 | Up |
| Parl | 55.29065797 | 6.91748E-07 | Up |
| Prrc1 | 38.51329089 | 0.000130699 | Up |
| Pls1 | 37.13477594 | 0.001519009 | Up |
| Ppt1 | 22.11907375 | 0.000633465 | Up |
| Acacb | 8.228364618 | 0.038200743 | Up |
| F10 | 7.519057256 | 0.013600664 | Up |
| Alg3 | 6.087758099 | 0.000589624 | Up |
| Alg9 | 6.032476106 | 0.02197169 | Up |
| Mtatp6 | 5.3535673 | 0.039662928 | Up |
| Hivep2 | 5.266865881 | 0.033970323 | Up |
| Dnaaf1 | 4.610336976 | 0.046147713 | Up |
| Ankh | 4.596017273 | 0.045113273 | Up |
| Pnpt1 | 4.207588653 | 0.047194289 | Up |
| Eef1e1 | 3.876760468 | 0.048442509 | Up |
| Atp2a3 | 2.096047659 | 0.001659069 | Up |
| Cep170 | 1.733556236 | 0.036412128 | Up |
| Atp6v1g1 | 1.667234759 | 0.012244375 | Up |
| Fabp7 | 1.652852746 | 0.015147163 | Up |
| Cacna1c | 1.6468427 | 0.0124233 | Up |
| Mapre2 | 1.632963325 | 0.001328275 | Up |
| Srrm4 | 1.554683099 | 0.030998752 | Up |
| Hint1 | 1.52040744 | 0.033146344 | Up |

*Downregulated DEPs*

| **Gene name** | **db/db AA/db/db TA Ratio** | ***p* value** | **Regulated Type** |
| --- | --- | --- | --- |
| Def6 | 0.005964673 | 4.88892E-10 | Down |
| Ube2v1 | 0.008922824 | 4.24348E-06 | Down |
| Scaf4 | 0.013561768 | 1.97758E-09 | Down |
| Krt28 | 0.013788869 | 0.000149203 | Down |
| Cpeb3 | 0.014145854 | 7.81154E-08 | Down |
| Bcas2 | 0.015013791 | 6.17618E-10 | Down |
| Wdr7 | 0.018836044 | 1.07421E-06 | Down |
| Commd6 | 0.02708622 | 1.32365E-07 | Down |
| Scarf2 | 0.033457596 | 1.0857E-07 | Down |
| Scara5 | 0.033511102 | 1.63452E-07 | Down |
| Nefl | 0.043227649 | 4.25753E-08 | Down |
| Polr2g | 0.04938229 | 1.57536E-08 | Down |
| Ppp1r14c | 0.060419251 | 0.000163178 | Down |
| Emp2 | 0.064692762 | 1.3814E-08 | Down |
| Gna14 | 0.067025347 | 7.42904E-08 | Down |
| Gnl1 | 0.074319278 | 4.05436E-06 | Down |
| Thada | 0.076436798 | 5.97416E-07 | Down |
| Prodh | 0.107270793 | 0.049204447 | Down |
| Cmtr1 | 0.120233293 | 0.010865337 | Down |
| Pqbp1 | 0.159963099 | 0.026348754 | Down |
| Mrpl21 | 0.169001004 | 0.032731591 | Down |
| Adam9 | 0.174782638 | 0.024769126 | Down |
| Casp7 | 0.177292926 | 0.006268177 | Down |
| Mvk | 0.193574852 | 0.031612288 | Down |
| Slc9a1 | 0.19375938 | 0.039791194 | Down |
| Rars2 | 0.196004596 | 0.039642312 | Down |
| Gripap1 | 0.202289867 | 0.031528722 | Down |
| Strn | 0.207668296 | 0.042549676 | Down |
| Lpgat1 | 0.207869717 | 0.033459757 | Down |
| Pofut1 | 0.208455031 | 0.040229537 | Down |
| Mras | 0.240737327 | 0.049344579 | Down |
| Cog7 | 0.247971911 | 0.042362785 | Down |
| Pid1 | 0.247980902 | 0.048096898 | Down |
| Dsc1 | 0.383951491 | 0.044064421 | Down |
| Olfml2b | 0.42659739 | 0.003171104 | Down |
| Dnm3 | 0.474758222 | 0.004942021 | Down |
| Fkbp8 | 0.477970081 | 0.006631276 | Down |
| Hdac2 | 0.496216287 | 8.21779E-05 | Down |
| Krt77 | 0.496662282 | 0.030110594 | Down |
| Vamp8 | 0.512116374 | 0.024771197 | Down |
| Dpp4 | 0.591337145 | 0.004091604 | Down |
| Ube2d2 | 0.631635942 | 0.016115922 | Down |
| Rtn3 | 0.638190976 | 0.005325192 | Down |
| Gpd1 | 0.643119573 | 0.014362493 | Down |
| Sub1 | 0.654319797 | 0.020836588 | Down |
| Msi2 | 0.659825046 | 0.010620021 | Down |

**Table S4.** List of upregulated and downregulated DEPs in db/db TA vs db/m^+^ TA.

*Upregulated DEPs*

| **Gene name** | **db/db TA/db/m^+^ TA Ratio** | ***p* value** | **Regulated Type** |
| --- | --- | --- | --- |
| Crispld2 | 165.6445369 | 1.39123E-08 | Up |
| Trappc6b | 95.59422414 | 1.77627E-08 | Up |
| Ptges2 | 83.37471312 | 2.03667E-06 | Up |
| Eipr1 | 80.3249145 | 3.73418E-07 | Up |
| Slc16a3 | 76.42314453 | 5.07285E-08 | Up |
| Tgfbrap1 | 71.45871566 | 1.2096E-07 | Up |
| Rab9a | 67.68699496 | 3.69446E-10 | Up |
| Tubb4a | 64.93448174 | 7.91543E-08 | Up |
| Ptpmt1 | 59.3431247 | 3.45704E-08 | Up |
| Hbb-bh1 | 53.60838885 | 8.50894E-06 | Up |
| Dhcr24 | 46.51698635 | 1.16814E-11 | Up |
| Gtf2f2 | 43.20417043 | 5.55869E-08 | Up |
| Akt3 | 43.18781521 | 4.17703E-06 | Up |
| Atp6ap2 | 41.56284644 | 6.18023E-10 | Up |
| Cbx5 | 32.38504957 | 9.951E-07 | Up |
| Elac2 | 25.88540952 | 3.34261E-07 | Up |
| Parp4 | 24.14566407 | 6.92003E-08 | Up |
| Phkb | 17.95381846 | 5.72073E-07 | Up |
| Serinc1 | 17.22278363 | 1.12999E-07 | Up |
| Carnmt1 | 15.29225001 | 1.16996E-05 | Up |
| Thada | 13.08270399 | 5.97416E-07 | Up |
| Gripap1 | 9.106461677 | 0.007311978 | Up |
| Plbd2 | 7.982565046 | 0.022081873 | Up |
| Ccdc43 | 7.838043944 | 0.030705521 | Up |
| Angel2 | 7.462045755 | 0.008523633 | Up |
| Bpifa2 | 7.262817399 | 0.024302459 | Up |
| Xpo5 | 5.939410474 | 0.022949 | Up |
| Cops7b | 5.694254524 | 0.017002295 | Up |
| Exoc7 | 5.612500325 | 0.034683171 | Up |
| Pts | 5.443314569 | 0.0375749 | Up |
| Pgs1 | 5.255976513 | 0.038541483 | Up |
| Alg1 | 5.244413417 | 0.034878257 | Up |
| Map2 | 5.176319252 | 0.043767964 | Up |
| Cnmd | 4.950808032 | 0.040101508 | Up |
| Dnajc24 | 4.917144172 | 0.044581746 | Up |
| Irgq | 4.837327083 | 0.034775456 | Up |
| Prr19 | 4.722872654 | 0.047333397 | Up |
| Ctnnbl1 | 4.715449338 | 0.043261913 | Up |
| Commd6 | 4.600702268 | 0.040101954 | Up |
| Tdrd3 | 4.591333801 | 0.037545285 | Up |
| Lpgat1 | 4.551003693 | 0.036806092 | Up |
| Snrpg | 4.54218346 | 0.047130219 | Up |
| Rbm28 | 4.443507431 | 0.042609066 | Up |
| Prrt2 | 4.433646254 | 0.049944332 | Up |
| Pxdc1 | 4.269589333 | 0.045904249 | Up |
| Atg16l1 | 4.198448733 | 0.028124208 | Up |
| Ap4s1 | 4.080360996 | 0.003223872 | Up |
| Smad1 | 4.078318961 | 0.048672614 | Up |
| Arglu1 | 4.064968453 | 0.049599323 | Up |
| Itfg1 | 3.831358258 | 0.007603295 | Up |
| Mcrip1 | 3.784465785 | 0.01340384 | Up |
| Metap1 | 3.457522128 | 0.046563372 | Up |
| Krt10 | 2.672542813 | 0.029083181 | Up |
| Alpl | 2.441394971 | 0.000826286 | Up |
| Hmgcs2 | 2.413111275 | 0.000972434 | Up |
| Pnpla2 | 2.367219799 | 0.000638129 | Up |
| S100a4 | 2.312384655 | 0.005099666 | Up |
| Fabp3 | 2.268416066 | 0.003820792 | Up |
| Serpine1 | 2.221591852 | 0.018846268 | Up |
| Slitrk4 | 2.105251284 | 0.031437645 | Up |
| Tpm1 | 2.087180668 | 0.029541007 | Up |
| Zc3hc1 | 2.076995134 | 0.035551621 | Up |
| Tpm2 | 2.058193868 | 0.030876221 | Up |
| Ccn5 | 1.972020967 | 0.004837174 | Up |
| Pcsk5 | 1.962672218 | 0.011831166 | Up |
| Skic3 | 1.871337527 | 0.005412687 | Up |
| Cyp2b10 | 1.871167564 | 0.007008234 | Up |
| Mfap2 | 1.854283353 | 0.010452394 | Up |
| Specc1 | 1.822382687 | 0.002278517 | Up |
| Tnc | 1.765764581 | 0.006230216 | Up |
| Cirbp | 1.746379604 | 0.017748541 | Up |
| Adgre5 | 1.746008912 | 0.049897092 | Up |
| Il17b | 1.742487654 | 0.015226938 | Up |
| S100a6 | 1.723385824 | 0.020504233 | Up |
| Plp2 | 1.717946425 | 0.001195129 | Up |
| Dpep1 | 1.712934275 | 0.005877125 | Up |
| Postn | 1.710396189 | 0.006816966 | Up |
| Myo9b | 1.686812528 | 0.033170565 | Up |
| Ptk2 | 1.673738632 | 0.044312388 | Up |
| Fmod | 1.672108376 | 0.005100813 | Up |
| Flad1 | 1.672102425 | 0.011988228 | Up |
| Rala | 1.670872978 | 0.021026222 | Up |
| Nrdc | 1.666635875 | 0.036285439 | Up |
| Art4 | 1.665132655 | 0.010686936 | Up |
| Mfap4 | 1.663165619 | 0.019480149 | Up |
| Fcsk | 1.656100957 | 0.007835622 | Up |
| Mbl2 | 1.644580739 | 0.038766808 | Up |
| Slmap | 1.641451314 | 0.001336587 | Up |
| Vcan | 1.640998873 | 0.031977565 | Up |
| Xrcc5 | 1.633702199 | 0.013699123 | Up |
| Phgdh | 1.622597397 | 0.004948602 | Up |
| Grb2 | 1.613421505 | 0.000271814 | Up |
| Ccdc22 | 1.604730315 | 0.005286932 | Up |
| Ate1 | 1.593244354 | 0.033283986 | Up |
| Acot7 | 1.591729885 | 0.01666224 | Up |
| Copz1 | 1.591513802 | 0.013485222 | Up |
| Ahcyl2 | 1.589051435 | 0.039502209 | Up |
| Vps25 | 1.588973363 | 0.026297733 | Up |
| Ptpn4 | 1.587147383 | 0.014394239 | Up |
| Prkar2a | 1.582955607 | 0.006021597 | Up |
| Emp2 | 1.580294139 | 0.008663223 | Up |
| Fbln2 | 1.577127428 | 0.007161398 | Up |
| Sema3f | 1.57000945 | 0.017947077 | Up |
| Sh3bgr | 1.568777412 | 0.012972941 | Up |
| Rplp2 | 1.563562368 | 0.029396541 | Up |
| Cnn3 | 1.561686834 | 0.029238228 | Up |
| Galnt16 | 1.560539996 | 0.024341843 | Up |
| Eef2k | 1.557323038 | 0.024700512 | Up |
| Rbm3 | 1.555669143 | 0.018403872 | Up |
| Lgals3 | 1.555320049 | 0.024722851 | Up |
| Plgrkt | 1.554128728 | 0.045113358 | Up |
| Rnf123 | 1.552440035 | 0.01535083 | Up |
| Ykt6 | 1.550036148 | 0.004657095 | Up |
| Myl6 | 1.549995309 | 0.001632585 | Up |
| Mapk9 | 1.548403397 | 0.016439617 | Up |
| Bckdhb | 1.548050113 | 0.001237013 | Up |
| Ncam1 | 1.546927786 | 0.004609496 | Up |
| Ror1 | 1.54638133 | 0.018448564 | Up |
| Ptprz1 | 1.543981655 | 0.004557727 | Up |
| Sh3bgrl3 | 1.53736523 | 0.015651036 | Up |
| Pole | 1.536976418 | 0.028075792 | Up |
| Fn1 | 1.534316368 | 0.025862778 | Up |
| Card19 | 1.522335794 | 0.010710132 | Up |
| Zw10 | 1.521595345 | 0.048675869 | Up |
| Olfml3 | 1.516269211 | 0.015760586 | Up |
| Armc1 | 1.511638274 | 0.025918829 | Up |
| Nrbp1 | 1.501209331 | 0.005136717 | Up |

*Downregulated DEPs*

| **Gene name** | **db/db TA/db/m^+^ TA Ratio** | ***p* value** | **Regulated Type** |
| --- | --- | --- | --- |
| Coro1a | 0.008738156 | 2.50041E-05 | Down |
| C8a | 0.009645623 | 8.36753E-06 | Down |
| Eif2b4 | 0.010024066 | 8.21574E-09 | Down |
| Elmo2 | 0.012066492 | 6.63776E-06 | Down |
| Pls1 | 0.016019522 | 7.27103E-05 | Down |
| Scd1 | 0.019014826 | 0.000246005 | Down |
| Serpina1e | 0.020161069 | 0.009898584 | Down |
| Washc1 | 0.070926211 | 0.002115983 | Down |
| Fasn | 0.093657319 | 0.000112995 | Down |
| Acacb | 0.094379139 | 0.015400791 | Down |
| Fam210b | 0.121435578 | 0.015763953 | Down |
| Mup18 | 0.131840376 | 0.027070131 | Down |
| Wdsub1 | 0.132915893 | 1.81435E-09 | Down |
| C1qc | 0.182216584 | 0.046719607 | Down |
| Mrpl23 | 0.221042701 | 0.039531274 | Down |
| Hmga1 | 0.249891408 | 0.047687359 | Down |
| Ndufaf7 | 0.266056412 | 0.044931748 | Down |
| Sfrp1 | 0.279642576 | 0.049008816 | Down |
| Poldip3 | 0.297718118 | 0.043915825 | Down |
| Trim33 | 0.306552439 | 0.041682569 | Down |
| Hp | 0.340277459 | 0.036364504 | Down |
| Mbl1 | 0.371957395 | 0.014537762 | Down |
| Acaca | 0.373808933 | 0.016332453 | Down |
| Acly | 0.379892983 | 0.003937346 | Down |
| C8g | 0.383937224 | 0.009843443 | Down |
| Pc | 0.428541207 | 0.040118596 | Down |
| Prxl2a | 0.470910358 | 0.018069947 | Down |
| Saa4 | 0.479537382 | 0.022237662 | Down |
| Fgg | 0.486170854 | 0.00395534 | Down |
| Nudt2 | 0.49328247 | 0.045368005 | Down |
| Gpd1 | 0.50008687 | 0.006454252 | Down |
| Fga | 0.505576548 | 0.003328951 | Down |
| Tst | 0.511104349 | 0.001853196 | Down |
| Fgb | 0.513423167 | 0.001520991 | Down |
| Saa1 | 0.51673715 | 0.047671131 | Down |
| Ntn1 | 0.520519461 | 0.000891899 | Down |
| Me1 | 0.552994837 | 0.001874988 | Down |
| Echdc1 | 0.572132289 | 0.014398498 | Down |
| Hint1 | 0.57446943 | 0.031220382 | Down |
| Mrc1 | 0.584042104 | 0.029309141 | Down |
| Nup85 | 0.591705997 | 0.001700704 | Down |
| Scara5 | 0.616461273 | 0.005068529 | Down |
| Aacs | 0.626240522 | 0.039741052 | Down |
| Acss2 | 0.626621383 | 0.036880722 | Down |
| Aldh4a1 | 0.63138741 | 0.008650856 | Down |
| Pygl | 0.635310496 | 0.026758294 | Down |
| Pgd | 0.643681425 | 0.009671368 | Down |
| Ddhd2 | 0.648671186 | 0.004622414 | Down |
| Cmpk2 | 0.648803445 | 0.027886636 | Down |
| C4b | 0.658082757 | 0.047585539 | Down |
| Phospho1 | 0.661380248 | 0.014641819 | Down |

**Table S5.** List of upregulated and downregulated DEPs in db/db AA vs db/m^+^ AA.

*Upregulated DEPs*

| **Gene name** | **db/db AA/db/m^+^ AA Ratio** | ***p* value** | **Regulated Type** |
| --- | --- | --- | --- |
| Mcpt2 | 110.2982381 | 0.029022669 | Up |
| Rps24 | 70.13751085 | 0.024276009 | Up |
| Arsa | 62.06849872 | 0.024228849 | Up |
| Mme | 49.92660801 | 0.025049704 | Up |
| Plekhf1 | 41.73432065 | 0.024330791 | Up |
| Elmo2 | 39.38414617 | 0.030361163 | Up |
| Prrc1 | 38.51329089 | 0.000130699 | Up |
| Dnajc19 | 37.02966516 | 9.71316E-09 | Up |
| Lamtor3 | 24.49720017 | 0.024327653 | Up |
| Bpifa2 | 22.94901414 | 1.30579E-06 | Up |
| Scrn3 | 13.89184914 | 0.025711891 | Up |
| Npc1 | 13.22449837 | 0.024218366 | Up |
| Sfxn2 | 11.18471326 | 0.027256864 | Up |
| Ppp3r1 | 11.04927802 | 0.015778999 | Up |
| Arsb | 10.0631893 | 0.026109736 | Up |
| Ankh | 7.516453346 | 0.011504686 | Up |
| Alg3 | 6.087758099 | 9.82985E-05 | Up |
| Necap1 | 5.640967172 | 0.012416991 | Up |
| Crlf3 | 5.519168865 | 0.015638169 | Up |
| Cpa3 | 5.418262218 | 0.023968818 | Up |
| Trarg1 | 4.795976274 | 0.022453009 | Up |
| Rbm17 | 4.698892397 | 0.016670102 | Up |
| Mcpt3 | 4.199974779 | 0.030263465 | Up |
| Epdr1 | 4.160850265 | 0.023750279 | Up |
| Nup37 | 4.012550753 | 0.021605279 | Up |
| Tbc1d17 | 3.990790366 | 0.021669897 | Up |
| Pts | 3.981064419 | 0.023746677 | Up |
| Oxa1l | 3.764456926 | 0.027856283 | Up |
| Map2 | 3.731008399 | 0.02880508 | Up |
| Coq4 | 3.514786267 | 0.027686389 | Up |
| Rabggtb | 3.47669205 | 0.030566108 | Up |
| Uap1 | 3.459031724 | 0.028388489 | Up |
| Coq5 | 3.356057264 | 0.034804479 | Up |
| Dnaaf1 | 3.118185194 | 0.017194714 | Up |
| Spon2 | 3.105068563 | 0.03121108 | Up |
| Pls1 | 3.035087866 | 0.046178644 | Up |
| Tpcn1 | 2.769555959 | 0.038800822 | Up |
| Atg16l1 | 2.643250229 | 0.040403288 | Up |
| S100a4 | 2.617632889 | 0.000684574 | Up |
| Slitrk4 | 2.211388392 | 0.002371773 | Up |
| Fabp3 | 2.178228029 | 0.003037424 | Up |
| Hmgcs2 | 2.126702913 | 0.000184343 | Up |
| S100a6 | 2.060487274 | 0.000612482 | Up |
| Sncg | 1.96551714 | 0.001562206 | Up |
| Serpine1 | 1.936829361 | 0.001708432 | Up |
| Dnttip2 | 1.859095779 | 0.02088938 | Up |
| Hmgcl | 1.846529244 | 0.048646519 | Up |
| Wdr35 | 1.795061795 | 0.009640437 | Up |
| S100a13 | 1.769531586 | 0.00185491 | Up |
| Naprt | 1.709033113 | 0.011877664 | Up |
| Sod3 | 1.681567104 | 0.000792998 | Up |
| Tpm1 | 1.639094039 | 0.01080402 | Up |
| Sh3bgrl | 1.60506076 | 0.027218741 | Up |
| Cgnl1 | 1.601920069 | 0.047019928 | Up |
| Tpm2 | 1.601839079 | 0.0099051 | Up |
| Sema3c | 1.582417752 | 0.01907194 | Up |
| S100a11 | 1.574245945 | 0.03930399 | Up |
| Ccdc88c | 1.571644409 | 0.022409104 | Up |
| Arhgdia | 1.564019815 | 0.04366642 | Up |
| Rps28 | 1.556157264 | 0.002486152 | Up |
| Impa1 | 1.544360548 | 0.040212351 | Up |
| Thbs1 | 1.538718764 | 0.019446252 | Up |
| Ccn5 | 1.525636793 | 0.001091924 | Up |
| Psma5 | 1.506224178 | 0.035371264 | Up |

*Downregulated DEPs*

| **Gene name** | **db/db AA/db/m^+^ AA Ratio** | ***p* value** | **Regulated Type** |
| --- | --- | --- | --- |
| Trim32 | 0.000314467 | 0.024026894 | Down |
| Serpina1e | 0.000635634 | 1.81247E-06 | Down |
| Mup18 | 0.003912071 | 1.71703E-07 | Down |
| Myo5a | 0.005756218 | 2.88821E-07 | Down |
| Sdhd | 0.006737081 | 5.53019E-06 | Down |
| Def6 | 0.008836762 | 0.024086886 | Down |
| Ift172 | 0.009871751 | 7.00459E-05 | Down |
| Ctsf | 0.010968816 | 5.74786E-12 | Down |
| Gys2 | 0.011517268 | 0.024385232 | Down |
| Bcas2 | 0.013160798 | 2.29368E-05 | Down |
| C8b | 0.014335711 | 5.88467E-05 | Down |
| Szt2 | 0.014689428 | 1.18837E-10 | Down |
| Gvin1 | 0.014696261 | 0.001336312 | Down |
| Fbxl4 | 0.020074896 | 0.026677495 | Down |
| Shc1 | 0.020545445 | 0.024475529 | Down |
| Trabd | 0.022404512 | 0.024062592 | Down |
| Fbxo2 | 0.02642753 | 0.00012616 | Down |
| Pof1b | 0.028583255 | 0.024247206 | Down |
| Scara5 | 0.034904881 | 0.024512617 | Down |
| Aspg | 0.03528394 | 0.024191498 | Down |
| Cdc34 | 0.036126294 | 0.024426606 | Down |
| Wdr7 | 0.037574323 | 0.024858437 | Down |
| Agfg2 | 0.039335292 | 0.000458925 | Down |
| Oxr1 | 0.042104405 | 0.02500549 | Down |
| Mindy1 | 0.042182105 | 0.024352579 | Down |
| Tap1 | 0.043898739 | 6.35185E-06 | Down |
| Trmu | 0.044317474 | 0.0313546 | Down |
| Krt73 | 0.046100641 | 0.006443065 | Down |
| Polr2g | 0.04965428 | 0.025099494 | Down |
| Scarf2 | 0.051560759 | 0.024824595 | Down |
| Evpl | 0.061940458 | 0.026550522 | Down |
| Ppp1r14c | 0.062724961 | 0.025992256 | Down |
| Stk17b | 0.063322154 | 0.025524396 | Down |
| Eno2 | 0.067070682 | 0.0112684 | Down |
| Ccdc102a | 0.06796153 | 1.68047E-10 | Down |
| Iigp1 | 0.069091154 | 0.025646448 | Down |
| Ctsk | 0.077359323 | 0.001521453 | Down |
| Gnl1 | 0.079132695 | 0.024317184 | Down |
| Fasn | 0.08263816 | 0.00038443 | Down |
| Gna14 | 0.086503331 | 0.024654681 | Down |
| Znrd2 | 0.09538397 | 0.02429816 | Down |
| Thrsp | 0.126402059 | 0.022180966 | Down |
| Mrpl21 | 0.135817165 | 0.011134806 | Down |
| Emp2 | 0.143391613 | 0.029805018 | Down |
| Mamdc2 | 0.154039089 | 0.013942511 | Down |
| Tbl3 | 0.159423321 | 0.026619145 | Down |
| Aga | 0.161023811 | 0.014097637 | Down |
| Man2a2 | 0.170900562 | 0.006384351 | Down |
| Pdpn | 0.171990311 | 0.001743818 | Down |
| Ppp2r1b | 0.178861735 | 0.013382451 | Down |
| Dhcr7 | 0.179656029 | 0.01433327 | Down |
| Arvcf | 0.186673995 | 0.045468314 | Down |
| Cpd | 0.192036708 | 0.026102659 | Down |
| Ppie | 0.223238451 | 0.019061177 | Down |
| Dsc1 | 0.231058324 | 0.012222581 | Down |
| Gpd1 | 0.239657134 | 0.0001977 | Down |
| Aldh1a7 | 0.251519585 | 0.031998542 | Down |
| Cggbp1 | 0.264316297 | 0.024025866 | Down |
| Nrp1 | 0.268480979 | 0.042391892 | Down |
| Cog7 | 0.278251141 | 0.023115509 | Down |
| Mpv17 | 0.286682394 | 0.02957078 | Down |
| Nccrp1 | 0.290845375 | 0.039851465 | Down |
| Acaca | 0.291266815 | 0.016445397 | Down |
| Acly | 0.298846412 | 0.001891316 | Down |
| Coro7 | 0.299608638 | 0.032030645 | Down |
| Srcin1 | 0.338223187 | 0.00158563 | Down |
| Fcn1 | 0.378410002 | 0.039879884 | Down |
| Aacs | 0.383576886 | 0.003806279 | Down |
| Usp4 | 0.384664206 | 0.04040978 | Down |
| Me1 | 0.421197654 | 0.005990745 | Down |
| Pfn1 | 0.421540291 | 0.002331982 | Down |
| Dnajc24 | 0.451203327 | 0.046115502 | Down |
| Pcyt2 | 0.470491654 | 0.047672786 | Down |
| Nes | 0.476148959 | 0.010619758 | Down |
| Acsl5 | 0.47946899 | 0.000920478 | Down |
| Acss2 | 0.486655939 | 0.009712893 | Down |
| Tapbp | 0.488111233 | 0.003012768 | Down |
| Scyl2 | 0.498190619 | 0.01700897 | Down |
| Tst | 0.51768825 | 0.008006307 | Down |
| Zbtb20 | 0.518406502 | 0.028994674 | Down |
| Tkt | 0.518942739 | 0.004854463 | Down |
| Mbl1 | 0.520149203 | 0.024120727 | Down |
| Fcsk | 0.526059263 | 0.045971537 | Down |
| Rigi | 0.531831409 | 0.01993411 | Down |
| Pgd | 0.553029517 | 0.007235306 | Down |
| Fgg | 0.563134843 | 0.039729618 | Down |
| Aldh4a1 | 0.564347357 | 0.036371681 | Down |
| Hk2 | 0.569141487 | 0.006441716 | Down |
| Rpl35 | 0.574178768 | 0.03799953 | Down |
| H2-L | 0.577624926 | 0.000430201 | Down |
| Crocc | 0.58334651 | 0.014313973 | Down |
| Mt-Cyb | 0.601067807 | 0.037295114 | Down |
| Stat1 | 0.602649669 | 0.025180621 | Down |
| Vtn | 0.603157555 | 0.00874971 | Down |
| Txnrd2 | 0.609478309 | 0.027957362 | Down |
| Pygl | 0.609604217 | 0.031572436 | Down |
| Coq7 | 0.612069904 | 0.032281347 | Down |
| Tspan31 | 0.615057773 | 0.00430539 | Down |
| Gltp | 0.618080286 | 0.026817451 | Down |
| Gsta4 | 0.619070116 | 0.013753841 | Down |
| Map3k20 | 0.622231562 | 0.002511082 | Down |
| S100a16 | 0.623042075 | 0.014580025 | Down |
| Coasy | 0.626397878 | 0.025455387 | Down |
| Rpl18a | 0.630015094 | 0.002897951 | Down |
| Phospho1 | 0.632471787 | 0.039424413 | Down |
| Igfbp6 | 0.632788288 | 0.001941609 | Down |
| Tbl2 | 0.638909594 | 0.025022595 | Down |
| Lmcd1 | 0.646360333 | 0.006859746 | Down |
| Taldo1 | 0.648637101 | 0.010838595 | Down |
| H2-K1 | 0.653054344 | 0.00112346 | Down |
| Gja1 | 0.666541692 | 0.03989397 | Down |

**Table S6.** Antibodies used in this study.

| **Antibody** | **Supplier** | **Cat no.** | **Dilution** |
| --- | --- | --- | --- |
| Anti-Phospho-eNOS (Ser1177) | Cell Signaling Technology | #9571S | 1:1000 |
| Anti-eNOS | BD Transduction Laboratory | #610297 | 1:1000 |
| Anti-Phospho-AMPKα (Thr172) | Cell Signaling Technology | #2535 | 1:1000 |
| Anti-AMPK | Cell Signaling Technology | #2532 | 1:1000 |
| Anti-β-tubulin | Transgen Biotech | HC101 | 1:1000 |
| Anti-DPEP1 | Proteintech | 12222-1-AP | 1:1000 |
| Anti-GR | Cell Signaling Technology | #12041 | 1:1000 |
| Anti-Histone H3 | Cell Signaling Technology | #9715 | 1:1000 |

**Table S7.** RT-PCR primers for detection of mouse genes.

| **Gene name** | **Primer sequences** |
| --- | --- |
| Sele | F: GGACACCACAAATCCCAGTCTG  R: TCGCAGGAGAACTCACAACTGG |
| Icam1 | F: AAACCAGACCCTGGAACTGCAC  R: GCCTGGCATTTCAGAGTCTGCT |
| Il-6 | F: TACCACTTCACAAGTCGGAGGC  R: CTGCAAGTGCATCATCGTTGTTC |
| Mcp1 | F: GCTACAAGAGGATCACCAGCAG  R: GTCTGGACCCATTCCTTCTTGG |
| Tnfα | F: GGTGCCTATGTCTCAGCCTCTT  R: GCCATAGAACTGATGAGAGGGAG |
| Vcam1 | F: GCTATGAGGATGGAAGACTCTGG  R: ACTTGTGCAGCCACCTGAGATC |
| Gapdh | F: CATCACTGCCACCCAGAAGACTG  R: ATGCCAGTGAGCTTCCCGTTCAG |
| HO-1 | F: CACTCTGGAGATGACACCTGAG  R: GTGTTCCTCTGTCAGCATCACC |
| Klf2 | F: CACCTAAAGGCGCATCTGCGTA  R: GTGACCTGTGTGCTTTCGGTAG |
| Nox1 | F: CTCCAGCCTATCTCATCCTGAG  R: AGTGGCAATCACTCCAGTAAGGC |
| Nox2 | F: TGGCGATCTCAGCAAAAGGTGG  R: GTACTGTCCCACCTCCATCTTG |
| Nox4 | F: CGGGATTTGCTACTGCCTCCAT  R: GTGACTCCTCAAATGGGCTTCC |
| Ucp2 | F: TAAAGGTCCGCTTCCAGGCTCA  R: ACGGGCAACATTGGGAGAAGTC |
| Serpina1e | F: GGAGCAAACTCTCAACAAGGAGC  R: GGTGATGCCCAGTGGACTCATG |
| Fasn | F: CACAGTGCTCAAAGGACATGCC  R: CACCAGGTGTAGTGCCTTCCTC |
| Mup18 | F: TGGGGAGAGCATCAATCTTCAG  R: AGAGTCCCTTTTCTTGGCAG |
| Bpifa2 | F: TGAACACAGCGGACCTTGGCAA  R: CCATTGCCGTTGGAAGACAGCT |
| C8b | F: ACTGTCAACGGGAGATGGAGCA  R: GTTGGTGTCCAGGATGTAGTGG |
| Tuba3a | F: CCACTACACCATCGGCAAAGAG  R: CTCCAAAGCTGTGGAAGATGAGG |
| Ctsf | F: CACAGCTCAGTATGGGATCACC  R: TTGGCTGGACTCATCTTCCTGC |
| Crispld2 | F: TGACGAGGTTGGTGGCTATGCA  R: GAAGGCGTTTCCGTTCTGAGGA |
| Szt2 | F: GCGTGGCACAATGAAAATCC  R: TATAAGGCCCAACCCGCTTC |
| Surf6 | F: GCACTGGATTTTCTGCGACAGC  R: CTCCTGCTTTCTCCGCTGTCTT |
| Mras | F: CCACCATTGAAGACTCCTACCTG  R: CGGAGTAGACAATGAGGAAGCC |
| Def6 | F: AAGACTGCCAGCCGCACCTATG  R: CCGCTTCTGTTTCAGGTCCTTG |
| Bcas2 | F: TGGCAGCGAAAGAACATGCAGC  R: GCTGGACAATCGTCCGCTCAAT |
| Scaf4 | F: AGTCGAGATCCAGGTCAGCATC  R: AGATCGAGAGCGAGGTGAGTGT |
| Stx16 | F: GATCCGTCAGATTGTGCAGTCC  R: CTTCGGTCTTGACACAGGACTG |
| Gna14 | F: TGACCAGGTTCTGGCTGAGTGT  R: ACGGAGGAGTTCAGAAACCAGG |
| Alg3 | F: CTGAGCATCAACCTTTTCCTGGC  R: CACCACGGAAGCCAAACTGAGT |
| Dhcr24 | F: CTGGAGAACCACTTCGTGGAAG  R: CTCCACATGCTTGAAGAACCAGG |
| Rab9a | F: GATCTGGAGGTGGACGGACATT  R: GTAAGCAGGCAACAGTCAGAACC |
| Atp6ap2 | F: TCACATTGCGGCAGCTCCGTAA  R: GTAGCACTTGCAGTTCGGAGAG |
| Eif2b4 | F: CAGTGAGGAACTCTCCAGGGAT  R: GCCAGTGACTTCCTTGGTTAGG |
| Trappc6b | F: CGACTACTCATTCAGCTGTCTGC  R: GGCAGGCATTGAAGAGACTTCAG |
| Ccdc102a | F: CCAGAAAGTGCTCCTCAAGGAG  R: GAGCATCTCCTGCTTGGTCTTG |
| Dnajc19 | F: CCTACTGCCAATAAAGGGAAGATC  R: GGTTTCAGACCTTCTTCCACCAG |
| Myo5a | F: TTGTGGAGCAGGCGAAGGAGAT  R: GCCTCTCATCATTCAGGTCAAGC |
| Dpep1 | F: TGTCCTGCGGACACTCTACCAT  R: CATCTCCTCTGTCCACAAGCCA |
| Acss2 | F: AGGTGACCAAGTTCTACACGGC  R: GTTGATGGGTTCACCTACTGTGC |
| Hmga1 | F: AGTGCCAACTCCGAAGAGACCT  R: TTGGTTTCCTCCCTGGAGCTGT |
| Baz1b | F: GTGCTGTCAAGAGCAGAAGGCT  R: CAGCCTTGTGTTTCCGCATTCG |
| Gtf3c1 | F: GCCGAGAAGTTGTGGATGAAGG  R: TGTGAGCCCATTTTCGGAGGTG |
| Nfkb2 | F: TGCTGATGGCACAGGACGAGAA  R: GTTGATGACGCCGAGGTACTGA |
| Wdr43 | F: ACGTTGCCTGACCTAGTAGAGC  R: CTGAGATGAGAACCGACTCCTT |
| Smarcc2 | F: TCTACTGCCTGTCGGCGGAATT  R: GCTCTCAGCATCTACCTGGTAG |
| Nrdc | F: CCAGACCTTCATCTTCCAGCTG  R: CAAGCAGCCTTGTGCGGTATTG |
| Smyd5 | F: CCAAGCCAGATTCTACCACACC  R: CTAGCCGACATTCTGCACTGCA |
| Smad1 | F: CTGAAGCCTCTGGAATGCTGTG  R: CAGAAGGCTGTGCTGAGGATTG |
| Sap18 | F: GTGAAGGAAGTCTACCCAGAAGC  R: CCAGACATGGTGCTGCCAATCT |
| Supt6h | F: GGCGTATCCAAGACCCTCTGAT  R: GTACAAGGCGTTAAGCAGCTCC |
| Stat5a | F: CCTGTTTGAGTCTCAGTTCAGCG  R: TGGCAGTAGCATTGTGGTCCTG |
| Mta1 | F: CTGGTGCTGAAGCAGGTAGTAC  R: CACACTGGATGAACTCTTCACGG |
| Hdac2 | F: GTTTTGTCAGCTCTCCACGGGT  R: CTTGGCATGATGTAGTCCTCCAG |
| Trim33 | F: TGAGACTGGCTCAGAATGCTGC  R: ACAGGAAACGGTCCAGCATGTC |
| Stat1 | F: GCCTCTCATTGTCACCGAAGAAC  R: TGGCTGACGTTGGAGATCACCA |

**Table S8.** Demographic characteristics of the included patients.

| **Variables** | **Mean ± SD** |
| --- | --- |
| No. of patients | 8 |
| No. of diabetic/non-diabetic patients | 4/4 |
| No. of males/females | 5/3 |
| Mean age | 63.75 ± 14.25 |
